# Industrial electrocatalytic C–C coupling reaction of C_1_ liquid molecules for efficient ethanol synthesis

**DOI:** 10.1093/nsr/nwag220

**Published:** 2026-04-10

**Authors:** Jiani Han, Yaodong Yu, G A Bagliuk, Jianping Lai, Lei Wang

**Affiliations:** State Key Laboratory Base of Eco-Chemical Engineering, Ministry of Education, International Science and Technology Cooperation Base of Eco-chemical Engineering and Green Manufacturing, College of Chemistry and Molecular Engineering, Qingdao University of Science and Technology, Qingdao 266042, China; Shandong Engineering Research Center for Marine Environment Corrosion and Safety Protection, College of Environment and Safety Engineering, Qingdao University of Science and Technology, Qingdao 266042, China; State Key Laboratory Base of Eco-Chemical Engineering, Ministry of Education, International Science and Technology Cooperation Base of Eco-chemical Engineering and Green Manufacturing, College of Chemistry and Molecular Engineering, Qingdao University of Science and Technology, Qingdao 266042, China; Frantsevich Institute for Problems of Materials Science, National Academy of Sciences of Ukraine, Kyiv 02000, Ukraine; State Key Laboratory Base of Eco-Chemical Engineering, Ministry of Education, International Science and Technology Cooperation Base of Eco-chemical Engineering and Green Manufacturing, College of Chemistry and Molecular Engineering, Qingdao University of Science and Technology, Qingdao 266042, China; State Key Laboratory Base of Eco-Chemical Engineering, Ministry of Education, International Science and Technology Cooperation Base of Eco-chemical Engineering and Green Manufacturing, College of Chemistry and Molecular Engineering, Qingdao University of Science and Technology, Qingdao 266042, China

**Keywords:** ethanol electrosynthesis, liquid C_1_ molecules, C–C coupling, formic acid reduction, palladium-copper electrocatalyst

## Abstract

Electrochemical conversion of C_1_ feedstocks into ethanol (EtOH) offers a promising pathway for achieving efficient carbon cycling and sustainable fuel production. However, conventional electrocatalytic methods suffer from poor mass transfer and lengthy reaction pathways, hindering the concurrent achievement of high current density and selectivity and thus limiting industrial application. Herein, we propose a novel electrocatalytic C–C coupling pathway for EtOH synthesis from liquid formic acid C_1_ feedstock, achieving high selectivity at high current densities. At 500 mA cm^−2^, the PdCu electrocatalyst achieves an EtOH Faradaic efficiency of 84.68%, with a formation rate of 1970.1 μmol h^−1^ cm^−2^, demonstrating significantly superior overall performance compared to conventional gaseous C_1_ feedstock electrocatalytic systems. Theoretical and *operando* spectroscopic studies elucidate that lattice hydrogen at the *in situ*-formed PdH*_x_* sites on PdH_0.45_Cu/CNT promotes the conversion of electrophilic *HCOO to nucleophilic *C(OH)_2_, while adjacent Cu promotes asymmetric coupling of *HCOO and *C(OH)_2_. The PdCu electrocatalyst demonstrates outstanding industrial potential, with 120 h of stable operation at 800 mA cm^−2^ in an anion exchange membrane electrolyzer, validated by technical-economic analysis. Moreover, this mechanism is equally applicable to the efficient conversion of formaldehyde into EtOH. This research proposes a novel pathway for the industrial production of electro-synthesized EtOH.

## Teaser

This work presents a novel electrocatalytic pathway for ethanol synthesis from liquid formic acid via C–C coupling with high selectivity and current density, demonstrating industrial potential for sustainable fuel synthesis.

## INTRODUCTION

Ethanol (EtOH) is a versatile organic reagent used as an important renewable fuel with high energy density and is widely used in the chemical, pharmaceutical, and food industries [1,2]. Conventionally, petroleum refining and fermentation have been the dominant methods of producing EtOH on an industrial scale [3]. However, conventional industrial processes have several obvious drawbacks, including high energy consumption and environmental pollution during petroleum refining; fermentation processes require complex procedures and expensive enzymes [4]. Notably, the production of EtOH powered by renewable electricity under ambient conditions has emerged as a promising strategy for tackling severe environmental pollution and achieving sustainable development [5]. Compared to conversion pathways via biomass and other C_2_ organic compounds, the C–C coupling route for EtOH production is characterized by high product directionality, high reaction efficiencies, and more attractive value-added conversion of C_1_ feedstocks [6,7]. However, currently available C–C coupling pathways utilize gaseous C_1_ components (for example, CO_2_, CO, and CH_4_) as carbon sources, with the limitation of difficulty in achieving high selectivity at high current densities [6,8–10]. Therefore, there is an urgent necessity to explore alternative electrocatalytic C–C coupling routes for electro-synthesizing EtOH in industrial applications.

**Scheme 1.**
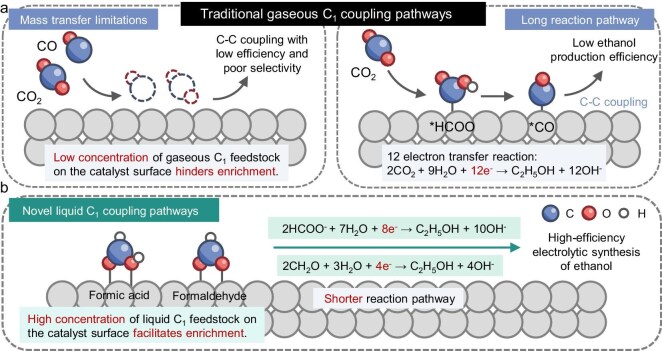
Schematic comparison of electrocatalytic C–C coupling pathways between (a) traditional gaseous C_1_ and (b) novel liquid C_1_ feedstocks.

To better understand the limitations of the current C–C coupling route, we further analyze the specific challenges posed by the utilization of gaseous C_1_ reactants (Scheme 1a). First, the low density of gaseous C_1_ reactants makes it difficult to capture and enrich [11]. The gaseous reactants are in contact with the electrolyte through gas diffusion electrodes, which have long mass transfer paths. As a result, nanobubbles generated by gaseous reactants attach to the electrode surfaces, reducing the exposed active-site area and leading to high overpotentials at high current densities [9,12]. Second, the higher activation energies required to activate the stable C–O and C–H bonds in CO_2_/CO and CH_4_ molecules make it more likely to favor competitive reactions that consume electrons and protons, leading to lower Faraday efficiency (FE) for EtOH at high current densities [13]. Third, from the perspective of electron efficiency, for instance, during the formation process of EtOH, the valence of the C atom in CO_2_ is reduced from +4 to −2, involving a reduction of six electrons per C atom. Consequently, the number of electrons transferred per unit time decreases due to multiple electron transfers with complex reaction paths, leading to slow kinetics and large overpotentials at high current densities. Fourth, the Gibbs free energy (Δ*G*) for the electrocatalytic conversion of CO_2_ to EtOH is ∼861.21 kJ mol^−1^, resulting in a significant thermodynamic barrier that requires substantial additional energy input to drive the reaction forward. Compared to gaseous reactants, liquid C_1_ sources are readily soluble in aqueous solutions and exhibit higher mass transfer efficiency, greater energy density, and a lower Δ*G*, which significantly reduces energy input requirements and enhances process efficiency (Scheme 1b) [14]. Therefore, the utilization of liquid C_1_ reactants for C–C coupling to generate EtOH not only enhances the efficiency of the electrocatalytic process but also represents a promising pathway for advancing the technology toward industrial-scale production.

In this work, we present a promising novel approach for C–C coupling electrosynthesis of EtOH using formic acid (FA) as a feedstock, achieving high selectivity at high current density and demonstrating potential for industrial application. It is observed that the optimum FE_EtOH_ reaches 84.68% at a current density of 500 mA cm^−2^ on PdCu/CNT. The corresponding formation rate is up to 1970.1 μmol h^−1^ cm^−2^, demonstrating significantly superior performance compared to conventional electrocatalytic C–C coupling systems using gaseous C_1_ feedstocks. Theoretical and *in situ* spectroscopic studies indicate that lattice hydrogen at the *in situ*-generated PdH*_x_* site in PdH_0.45_Cu/CNT enhances the formation of nucleophilic *C(OH)_2_ from electrophilic *HCOO. Subsequently, the adjacent Cu promotes the asymmetric coupling of nucleophilic and electrophilic intermediates. The PdCu electrocatalyst demonstrates outstanding industrial application potential in an anion exchange membrane (AEM) electrolyser, operating stably for 120 h at a high current density of 800 mA cm^−2^. A techno-economic analysis (TEA) reveals the industrial electrolytic EtOH synthesis cost is only $242 ton^−1^ (48.4% of the lowest market price), proving economic viability. This mechanism can also be applied to other electrophilic molecules such as formaldehyde, offering broad application prospects.

## RESULTS AND DISCUSSION

**Figure 1.**
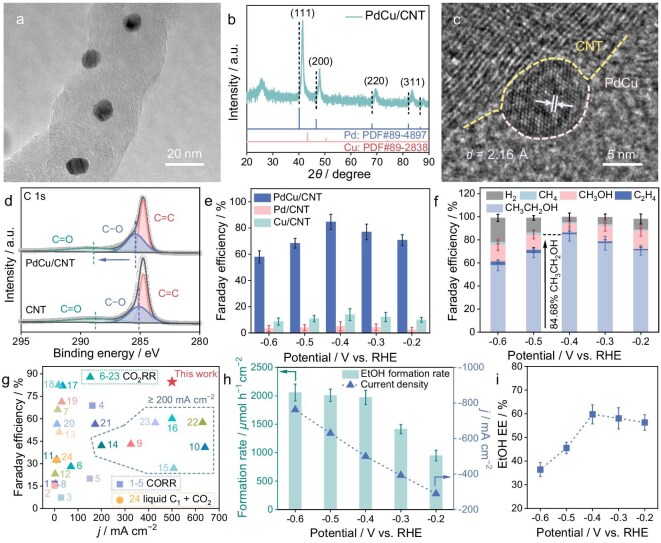
Characterization and electrocatalytic performance. (a) TEM image, (b) XRD pattern, (c) HR-TEM image of the as-prepared PdCu/CNT catalyst. (d) C 1s XPS spectra for PdCu/CNT and CNT. (e) FEs of EtOH production over various electrodes at different applied potentials with 2-h electrolysis. Error bars represent the standard deviation in three independent measurements. (f) FEs and product distributions at different potentials on PdCu/CNT. (g) Comparison of current densities and FEs on PdCu/CNT with related studies to generate EtOH from C_1_ feedstocks (Table S2 for more details). (h) EtOH formation rates and current densities over PdCu/CNT at various applied potentials. (i) Half-cell EEs of EtOH over PdCu/CNT.

To establish an efficient liquid C_1_ molecular electrocatalytic C–C coupling system, it is essential to define the structural foundation of the core catalyst. We fabricated the carbon nanotubes (CNTs) supported PdCu-based catalyst by a solvent-free microwave method and conducted systematic structural characterization (Fig. S1). The transmission electron microscope (TEM) provides insight into the morphology and microstructure of PdCu/CNT, involving particle shape, size, and distribution (Fig. 1a). The nanoparticles are uniformly grown on CNT, which is the skeleton for placing the nanomaterials, and the average size of the nanoparticles is ∼10 nm. Moreover, the nanoparticles loaded on CNT formed a carbon layer coating under microwave thermal radiation, suggesting a strong metal-support interaction (SMSI) between the nanoparticles and the carrier CNT [15]. The TEM images of Pd/CNT and Cu/CNT are shown in Fig. S2a and b. The X-ray diffraction (XRD) patterns of as-synthesized PdCu/CNT, Pd/CNT, CNT, and Cu/CNT are shown in Fig. 1b, Fig. S3a and b. The CNT exhibits a reflection at 2*θ* = 25.7°, representing C (002). The XRD spectrum of Pd/CNT shows four diffraction peaks at 40.1°, 46.6°, 68.1°, and 82.1°, which can be assigned to the (111), (200), (220), and (311) crystalline planes of the Pd, matching the standard patterns of Pd (PDF#89-4897) with a face-centered cubic (fcc) structure. The three diffraction peaks of Cu/CNT correspond to the (111), (200), and (220) planes of the fcc metallic Cu (PDF#89-2838). It is noteworthy that the four typical diffraction peaks of PdCu/CNT are positively shifted to higher angles compared with the standard patterns of Pd, demonstrating that the alloying induces the contraction of crystalline lattices caused by the partial replacement of Pd atoms (179 pm) by introduced Cu atoms (128 pm) with smaller atomic radius sizes [16]. Furthermore, in the XRD spectrum of PdCu/CNT, four distinct diffraction peaks are obtained at 41.5°, 48.0°, 69.4°, and 83.5°, and no other characteristic diffraction peaks of a single composition corresponding to pure Pd or Cu are observed, which further suggests the formation of a single-phase PdCu alloy [17]. Other samples with different Pd/Cu ratios also show single-phase PdCu alloys (Fig. S4). As the Pd content increases, the diffraction peaks are progressively shifted to higher angles [18]. Moreover, the detailed structural properties of PdCu/CNT were further confirmed by high-resolution transmission electron microscopy (HR-TEM). As shown in Fig. 1c, the lattice spacing of PdCu (2.16 Å) is smaller than the standard value of Pd (111) lattice spacing (Fig. S5a), confirming that the introduction of Cu leads to lattice contraction and alloying of PdCu, which is consistent with the XRD results. The HR-TEM image of Cu/CNT is shown in Fig. S5b, with a lattice spacing of 2.09 Å. The relatively high-angle-annular dark-field STEM (HAADF-STEM) and energy dispersive X-ray spectroscopy (EDS) elemental mapping images indicate that Pd and Cu are evenly distributed in nanoparticles on the CNT skeleton with an atomic ratio of 1:1, which is consistent with the results of inductively coupled plasma atomic emission spectroscopy (Fig. S6 and Table S1). The X-ray photoelectron spectroscopy (XPS) was performed to define the elemental composition and chemical state of the PdCu/CNT, Pd/CNT, and Cu/CNT. The C 1s XPS spectrum of PdCu/CNT displays multiple carbon species, comprising C=C (284.8 eV), C–O (285.4 eV), and C=O (289.2 eV) (Fig. 1d) [19]. Moreover, we synthesized alloyed PdCu nanoparticles (PdCu NPs) and physically mixed them with CNTs as a reference sample (Fig. S7a–c). As shown in Fig. 1d and Fig. S7d, XPS analysis reveals that the binding energies of the C–O and C=O functional groups in PdCu/CNT are higher than those in the physical mixture and pure CNT, indicating charge transfer between the metal and CNT in PdCu/CNT, thereby confirming the SMSI effect [20]. In the 3d XPS spectra of Pd in Pd/CNT, the dominant peaks located at 335.8 and 341.2 eV are indexed to the Pd 3p_5/2_ and Pd 3p_3/2_ of metallic Pd; the two peaks present at around 337.7 and 343.2 eV are attributed to Pd^2+^ 3p_5/2_ and Pd^2+^ 3p_3/2_ species, respectively (Fig. S8a) [21]. The Cu 2p spectrum of Cu/CNT reveals that the main peaks at 932.0 and 952.7 eV are contributed by Cu^0^/Cu^+^ 2p_3/2_ and 2p_1/2_, and the core peaks (933.8 and 953.8 eV) and satellite peaks (943.2 and 962.2 eV) of Cu^2+^ (Fig. S8b) [22,23]. The Cu LMM Auger spectra, the most reliable method for distinguishing Cu^0^ from Cu⁺, exhibit a kinetic energy peak at 918.7 eV, confirming the chemical state of Cu^0^ (Fig. S8c) [24]. The Pd 3d spectrum of PdCu/CNT is negatively shifted compared to Pd/CNT, whereas the binding energy for Cu 2p in PdCu/CNT is positively shifted to a higher value in comparison to that in Cu/CNT. These apparent shifts occur since the work function (Φ) of Pd (5.12 eV) is larger than that of Cu (4.65 eV), and there is a partial transfer of electrons from Cu to Pd in the PdCu alloy in PdCu/CNT [25]. Furthermore, the Pd 3d and Cu 2p XPS spectra of the PdCu NPs-CNT physical mixture and PdCu/CNT provide additional strong support for the SMSI effect (Fig. S9). The negative shift in Pd 3d binding energy and positive shift in Cu 2p binding energy observed in PdCu/CNT are attributed to the SMSI effect, facilitating directed electron transfer from CNTs to metal particles [26,27]. In contrast, physical mixtures lack a well-defined metal-support interface and thus exhibit no such characteristic shift, providing strong evidence for the SMSI effect in PdCu/CNT. The solvent-free microwave method was successfully scaled up 50-fold to produce PdCu/CNT, yielding ∼1.58 g of product with an efficiency of 81.9% (Fig. S10a) [15]. Figure S10b and c reveals a uniform distribution of nanoparticles on CNT, with a 1:1 Pd/Cu ratio on the nanoparticles. XRD and XPS analyses confirm the formation of the PdCu alloy (Fig. S11a–c).

After completing the structural characterization of the catalyst, further validation of the electrocatalytic performance is necessary to establish the relationship between structural properties and catalytic activity. The electrocatalytic performances of the PdCu/CNT, Pd/CNT, and Cu/CNT were evaluated under ambient conditions. All the potentials were calibrated to the reversible hydrogen electrode (RHE) according to the Nernst equation in this work. Hydrophilic carbon paper is utilized as its excellent wettability provides an efficient mass transport pathway for the liquid-phase FA reduction reaction (FRR), thereby significantly enhancing current density [28]. The electrochemical characterization of catalysts was conducted by linear scanning voltammetry. Compared to the other three catalysts, the current density of PdCu/CNT is obviously increased, and the corresponding onset potentials are positively shifted in the order of CNT, Cu/CNT, Pd/CNT, and PdCu/CNT (Fig. S12a). This indicates that PdCu/CNT exhibits excellent FRR activity. Over the PdCu/CNT electrode, the current density in the electrolyte that contained 1.0 M KOH + 1.0 M FA is significantly higher than that in the electrolyte containing only 1.0 M KOH, which is attributed to the reduction effect of FA (Fig. S12b). Furthermore, electrochemical impedance spectroscopy was evaluated on these four electrodes. Nyquist plot analysis indicates that FA, as a reaction substrate, effectively optimizes the charge transfer process at the electrode–electrolyte interface, enhancing the interfacial electron transfer efficiency (Fig. S13a). As shown in Fig. S13b, the PdCu/CNT electrode exhibits significantly lower impedance than other electrodes, confirming its minimal charge transfer resistance and thus ensuring faster electron transfer rates during the reaction. The electrochemical performance of the synthesized catalysts was further evaluated by cyclic voltammetry (CV), and the CV curves of Pd/CNT, Cu/CNT, and PdCu/CNT were tested at different scan rates (Fig. S14a–c). Subsequently, the capacitance of the double layer (C_dl_) was calculated from the CV curve as shown in Fig. S15a. The C_dl_ of PdCu/CNT is 9.80 mF cm^−2^, which is larger than that of Pd/CNT (8.77 mF cm^−2^) and the Cu/CNT (7.84 mF cm^−2^). Since the electrochemically active surface area (ECSA) was determined by the measurement of the C_dl_, the ECSA of the PdCu/CNT electrode (245 cm^2^) is also larger than that of Pd/CNT (219 cm^2^) and Cu/CNT (196 cm^2^), suggesting that more active sites of PdCu/CNT are exposed to the reaction (Fig. S15b).

Preliminary electrochemical investigations have demonstrated the catalytic advantages of PdCu/CNT, but the identification and quantification of reaction products are crucial for confirming the efficient generation of EtOH. Therefore, the liquid and gaseous products of the selective electroreduction of FA to EtOH were identified and quantified by ^1^H nuclear magnetic resonance (NMR) spectroscopy and gas chromatography (GC), respectively. The calibration curves for product identification using GC and ^1^H NMR are shown in Figs S16–S18. The concentration of products generated is calculated based on the calibration curves. Analysis of the fresh electrolyte prepared with commercial FA confirms the absence of detectable impurities (Fig. S19). Figure S20 compares ¹H NMR spectra obtained at different potentials, while Fig. S21 presents a comparison of NMR spectra before and after the reaction. Based on the NMR results, the amount of EtOH generated when the potential is greater than −0.4 V vs. RHE shows only a slight increase, but the FE shows a decreasing trend due to the large increase in current density. Consequently, the FEs of the products were determined only in the applied potential range from −0.6 to −0.2 V vs. RHE. The FEs of EtOH on PdCu/CNT, Pd/CNT, and Cu/CNT electrodes were calculated, with the PdCu/CNT electrode exhibiting the highest FE_EtOH_ (Fig. 1e). Figure 1f shows the FEs and distributions of products for H_2_, ethane (C_2_H_6_), methane (CH_4_), methanol (CH_3_OH), and EtOH on PdCu/CNT, with a total FE of ∼100%. At the applied potential of −0.4 V vs. RHE and a current density of 500 mA cm^−2^, the highest FE of 84.68% is achieved for EtOH on PdCu/CNT. Remarkably, in contrast to the electrochemical C_1_ feedstock coupling pathways reported in the literature, only our FA self-coupling pathway for EtOH generation simultaneously achieves high FE and low potential at high current density (Fig. 1g, Fig. S22, and Table S2). Relatedly, the EtOH formation rate on PdCu/CNT is up to 1970.1 μmol h^−1^ cm^−2^ at −0.4 V vs. RHE, which is 8.2 and 21.0 times higher than that on Cu/CNT (190.4 μmol h^−1^ cm^−2^) and Pd/CNT (93.8 μmol h^−1^ cm^−2^), respectively (Fig. 1h and Fig. S23a). Furthermore, large-scale-produced PdCu/CNT exhibits similarly high catalytic activity (Fig. S23b). As shown in Fig. 1i, the half-cell energy efficiency (EE) of EtOH, namely the ratio of the chemical energy stored in the main product EtOH to the electrical energy input to the reaction, was estimated. The EE of EtOH is 59.75% at −0.4 V vs. RHE on PdCu/CNT, demonstrating that most of the electrical energy is utilized for the generation of EtOH under this condition. As control experiments, the FEs and formation rates of various electrodes with different Pd/Cu ratios are calculated (Fig. S24a and b). Pd-enriched catalysts promote over-hydrogenation and unwanted HER. While Cu-enriched catalysts favor methanol formation, the decrease in Pd sites that provide H leads to insufficient post-coupling hydrogenation. The optimal Pd_*x*_Cu/CNT, with a Pd/Cu ratio of 1:1, maximizes the EtOH generation. As shown in Fig. S25, the effect of alkali metal cations in the MOH (M = Li^+^, Na^+^, K^+^, and Cs^+^) electrolyte at −0.4 V vs. RHE on the FRR is further explored. The FEs and the formation rates of EtOH follow a sequence of Li^+^ < Na^+^ < Cs^+^ < K^+^. This can be attributed to the structure of the interfacial water, which is affected by the value of *n* in the hydrated cation M^+^(H_2_O)*_n_* (where *n* = 22, 13, 7, and 6 for Li^+^, Na^+^, K^+^, and Cs^+^, respectively) [29–31]. High *n* cations form a dense water layer, increasing water order and hindering reactant diffusion, while low *n* cations form hydrophobic interfaces, promoting intermediate adsorption [32]. Although the low *n* value of Cs^+^ should theoretically facilitate coupling, the stronger electrode interaction leads to accumulation on the catalyst surface, blocking active sites, and inhibiting kinetics [33]. Therefore, KOH is an ideal electrolyte for FRR. Furthermore, the catalytic efficiency of FRR exhibits significant dependence on electrolyte pH, with optimal performance achieved in a 1.0 M KOH + 1.0 M FA electrolyte system (Fig. S26). This phenomenon is attributed to the suppression of water migration toward the catalyst under high pH conditions, which limits the possibility of H species access to the adsorbed intermediates [30]. Conversely, lowering the pH promotes competitive HER, thereby inhibiting the activity of FRR [34]. Furthermore, ten sequential electrolysis batches of FRR on PdCu/CNT exhibit similar current profiles at −0.4 V vs. RHE, while FE and formation rate maintain around 84.06% and 1960.1 μmol h^−1^ cm^−2^, respectively, during 10 successive cycles (Fig. S27a and b). PdCu/CNT exhibits high activity towards FRR during long-term electrolysis, and the EtOH obtained from the 20 h stability test was quantitatively analyzed by ^1^H NMR spectroscopy (Fig. S27c–e). However, the slight decay primarily stems from mass transfer limitations within the H-cell, leading to blockage of catalytic sites and intrinsic activity reduction, rather than the loss of active sites (Fig. S28) [35]. EDS elemental mapping confirms that PdCu/CNT still maintains a homogeneous distribution of Pd and Cu (Fig. S29). The minor decay and structural integrity post-stability testing are attributed to the following factors. First, the stability of the catalyst is guaranteed by the intrinsic stability of the PdCu alloy, which stems from the strong electronic interaction between Pd and Cu [36,37]. Second, the SMSI between the nanoparticles and the carrier CNT prevents nanoparticle agglomeration, anchors particles to inhibit migration, and facilitates electron transfer [15,38]. CNT conductivity enables rapid charge distribution to avoid localized potential extremes [39]. In contrast, unsupported PdCu NPs readily agglomerate, resulting in a noticeable reduction in effective active surface and consequently decreased catalytic performance (Fig. S30).

**Figure 2.**
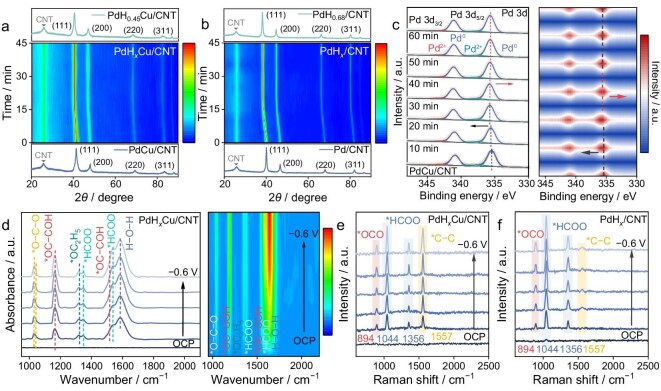
*In situ* spectroscopic studies. *In situ* electrochemical XRD patterns and corresponding two-dimensional spectra of (a) PdH*_x_*Cu/CNT and (b) PdH*_x_*/CNT. (c) Time-dependent quasi-*in situ* XPS spectra and the corresponding equi-contour plots for Pd 3d of PdH*_x_*Cu/CNT. (d) *In situ* FTIR spectra obtained during FRR at different potentials, along with the corresponding two-dimensional spectra of PdH*_x_*Cu/CNT. *In situ* Raman spectra under different potentials on (e) PdH*_x_*Cu/CNT and (f) PdH*_x_*/CNT.

Product analysis confirmed the highly efficient generation of EtOH, yet the structural evolution of the catalyst that facilitated this outcome during the catalytic process remains to be investigated using *in situ* electrochemical techniques. We have observed that the hydrogen storage capability of Pd enables dynamic hydrogen transfer to adsorbed intermediates, a critical step in modulating the nucleophilicity for subsequent coupling [40]. Therefore, it is reasonable to hypothesize that the reversible phase transition between Pd and Pd-hydride (PdH*_x_*) affects the selectivity and activity of Pd-based catalysts. *In situ* electrochemical XRD experiments were performed to investigate the formation process of PdH*_x_*. As shown by the negative peak shift in Fig. 2a and b, Figs S31 and S32, it is confirmed that the hydrogen enters the Pd lattice, resulting in lattice expansion of Pd, which indicates the interaction between hydrogen and Pd [41]. With the electrolysis time extension, the PdH*_x_* phase progressively develops, exhibiting a characteristic Pd diffraction pattern. The lattice parameters calculated from the positions of the (111) peaks are presented in Table S3, while the lattice parameter of PdCu/CNT follows Vegard’s law for Pd–Cu alloys with a 1:1 ratio. After about 25 min of reaction, the lattice parameter is calculated to be 3.86 Å, corresponding to a H/Pd molar ratio of ∼0.45 in PdH*_x_*Cu/CNT (Fig. 2a). As shown in Fig. 2b, the negative shift of the Pd/CNT diffraction peak is more noticeable, with a lattice parameter of 4.07 Å, corresponding to an H/Pd ratio of about 0.68 for PdH*_x_*/CNT [42]. Based on the HR-TEM and XRD results, the PdCu model was constructed by introducing Cu atoms into the Pd (111) crystal plane, and the Pd model was constructed using the Pd (111) facet (Fig. S33). Moreover, according to the *in situ* electrochemical XRD results, the PdH_0.45_Cu (111) and PdH_0.68_ (111) models are shown in Fig. S34. The quasi-*in situ* XPS measurements were conducted to further monitor real-time changes in chemical states and electronic interactions during the FRR process. Initially, the Pd 3d peak shifts to higher binding energies, confirming the Pd and H interaction and the formation of PdH*_x_* (Fig. 2c) [43]. This is consistent with the results shown by *in situ* electrochemical XRD, indicating the formation of the PdH*_x_* phase within ∼25 min. Concurrently, the Cu 2p peak exhibits a negative shift (Fig. S35), indicating electron transfer between Cu and Pd [44,45]. During the 30-min reaction phase, both the Pd 3d and Cu 2p peaks exhibit a slight shift toward lower binding energy, which is attributed to the reduction of existing M^2+^ to M^0^ [46]. In the subsequent stage, the binding energies of Pd 3d and Cu 2p remain essentially stable.

Following the elucidation of the PdH*_x_* phase mechanism, the specific reaction pathway for C–C coupling requires further validation. The isotopic label experiments combined with mass spectrometry (MS) were performed to investigate the C–C coupling pathway and validate intermediate steps. Different substrates were utilized in the comparative experiments, and the EtOH in the resulting electrolyte was analyzed [47]. As shown in Fig. S36, the signals of EtOH appear at *m/z* = 46, 47, and 48, corresponding to ^12^C–^12^C, ^13^C–^12^C, and ^13^C–^13^C, respectively. Characteristic fragments of EtOH include ^12^CH_3_^12^CH_2_OH (*m/z* = 31), ^13^CH_3_^12^CH_2_OH and ^12^CH_3_^13^CH_2_OH (*m/z* = 32), as well as ^13^CH_3_^13^CH_2_OH (*m/z* = 33) (Table S4) [48]. When ^13^C-FA and ^12^C-methanol are used as substrates, the EtOH signal distribution is consistent with that of the ^13^C-FA group, confirming that methanol (a minor by-product) does not participate in the C–C coupling reaction process. Additionally, similar results are obtained with other feedstocks (^13^C-FA + ^12^C-ethylene glycol and ^13^C-FA + ^12^C-oxalic acid), suggesting the proposed asymmetric C–C coupling mechanism and verifying the key coupling intermediates. Based on MS results, the coupling and hydrogenation steps in the FA coupling process, shown in Fig. S37, are proposed.

Isotope labeling has identified the overall pathway of C–C coupling, while the identification of key intermediates during the reaction is crucial for elucidating the mechanism. The *in situ* Fourier transform infrared (FTIR) spectroscopy measurements were conducted to further investigate the possible intermediates and the reaction mechanism of FRR (Fig. 2d, Figs S38 and S39). Based on the electrochemical experiments of FRR described above, a potential range of −0.2 to −0.6 V vs. RHE was selected. According to the relevant reports, the *in situ* FTIR peaks that appeared are assigned to the corresponding intermediates (Table S5). The peak observed at about 1639 cm^−1^ can be indexed to the H–O–H bending vibration of H_2_O. Peaks at ∼1378 and ∼1585 cm^−1^ are assigned to C–O symmetric and asymmetric stretching of *HCOO, respectively, which are related to the formate adsorption on the catalysts. Notably, the peaks observed at ca. 1035 cm^−1^ and ca. 1047 cm^−1^ can be ascribed to the symmetric and asymmetric OCO stretching vibrations of *C(OH)_2_, which is a key intermediate in the subsequent C–C bond formation process. The intensities of *HCOO and *C(OH)_2_ peaks increase with applied potential and are significantly stronger on Pd-containing catalysts (PdH*_x_*Cu/CNT and PdH*_x_*/CNT) than on Cu/CNT [49]. This indicates that the presence of Pd enhances the adsorption of formate and the formation of the key intermediate *C(OH)_2_. The OCO peaks on PdH*_x_*Cu/CNT can be observed from −0.2 to −0.6 V vs. RHE, suggesting *C(OH)_2_ intermediate is stabilized over a wider range of potentials. Moreover, peaks indicative of C-C bonded species (*OCCOH and *OC_2_H_5_) are observed at ∼1182, ∼1565, and ∼1349 cm^−1^, with intensities in the order of PdH*_x_*Cu/CNT > Cu/CNT > PdH*_x_*/CNT, confirming the dominant role of Cu in promoting C–C coupling [2]. The weak signals of these C–C species peaks on PdH*_x_*/CNT can be attributed to the rapid conversion of the *C(OH)_2_ intermediates to methanol, which is due to the excessive adsorption of the intermediate and the absence of C–C coupling sites. The corresponding side-reaction pathway b for methanol production on Pd/CNT is shown in Fig. S37. No obvious absorption peaks were observed in the characteristic stretching vibration region of *CO (∼2000–2100 cm^−1^) on three catalysts, proving the absence of *CO intermediates.

Furthermore, to guarantee the reliability of the results, *in situ* Raman spectroscopy measurements were performed to support the results obtained from *in situ* FTIR (Fig. 2e and f, and Fig. S40, and peak assignment in Table S6). The appearance of the Raman peaks around 1044, 1356, and 894 cm^−1^ corresponds to the *HCOO and *C(OH)_2_ intermediates. The intensities of these two peaks on PdH*_x_*/CNT and PdH*_x_*Cu/CNT are higher than those on Cu/CNT, which is consistent with the *in situ* FTIR results. The Raman peaks of C–C stretching are observed on Cu/CNT and PdH*_x_*Cu/CNT, but are not obvious on PdH*_x_*/CNT, indicating that Cu promotes the C–C coupling [49]. When compared at the same potential, the signal of the C–C stretching peak on PdH*_x_*Cu/CNT is higher than that on Cu/CNT, suggesting that the formation of EtOH via electrochemical C–C coupling is more favorable on PdH*_x_*Cu/CNT. The proposed reaction process is further elucidated by combining *in situ* FTIR and *in situ* Raman results, confirming that Pd sites promote the adsorption and activation of formate, while Cu sites guarantee the C–C coupling process [50].

**Figure 3.**
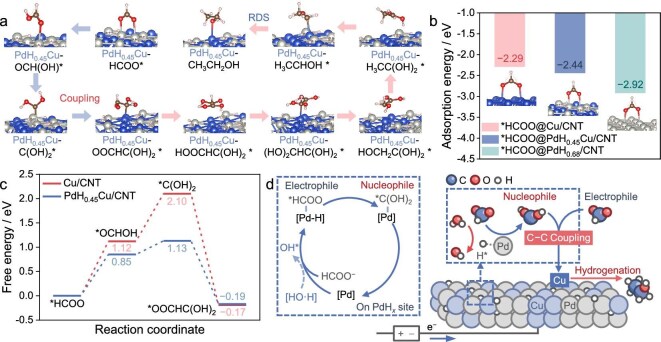
Mechanism investigations. (a) Optimized structures of all reaction intermediates involved in the pathways of FRR on PdH_0.45_Cu. (b) Free energy diagrams for *HCOO adsorption on Cu/CNT, PdH_0.45_Cu/CNT, and PdH_0.68_/CNT. (c) Free energy diagram for asymmetric coupling pathways on PdH_0.45_Cu/CNT and Cu/CNT. (d) Schematic illustration of the proposed C–C coupling mechanism via nucleophilic-electrophilic intermediates for EtOH formation on PdH_0.45_Cu/CNT.

*In situ* spectroscopy experiments captured the intermediates and reaction characteristics. To obtain mechanistic insights into the reaction, density functional theory (DFT) calculations were subsequently conducted for the formation of EtOH from the self-coupling of FA. The Cu model was constructed using the Cu (111) facet (Fig. S41). The optimized structures of all reaction intermediates involved in the pathways on PdH_0.45_Cu are shown in Fig. 3a. Initially, FA molecules form formate in an alkaline environment and are adsorbed on the surfaces of the three catalysts, with stable adsorption states (Fig. 3b). The adsorption strength of *HCOO on PdH_0.45_Cu/CNT is regulated by the introduced Cu, falling between Cu/CNT and PdH_0.68_/CNT, thereby avoiding excessive adsorption and facilitating adsorption compared to Cu/CNT. According to Gibbs free energy calculations, the PdH*_x_* site enhances the conversion of adsorbed *HCOO to *C(OH)_2_, with the energy barrier decreasing as the H/Pd molar ratio increases (Fig. S42). The related optimal structures of *HCOO activation to *C(OH)_2_ on Cu/CNT and PdH_0.68_/CNT are depicted in Fig. S43. As shown in Fig. S44, the O-monodentate-adsorbed *OCHOH intermediate is more readily converted to *C(OH)_2_ with a C-bound configuration [51]. Moreover, the side-reaction pathways for methanol production were also investigated (Fig. S45a). The values of Δ*G*_(*OCHOH-to-*CH2OH)_ on all three catalysts are significantly higher than those of Δ*G*_(*OCHOH-to-*C(OH)2)_, indicating that pathway a for methanol production is unfavorable (Fig. S45b). As shown in Figs S45c and S46, PdH_0.45_Cu/CNT and Cu/CNT are more favorable for the C–C coupling process. The intense adsorption of *C(OH)_2_ on PdH_0.68_/CNT favors methanol formation (side-reaction pathway b), which is in accordance with the *in situ* experimental results (Fig. S47) [49]. Therefore, the subsequent coupling steps of the DFT calculations focus on Cu/CNT and PdH_0.45_Cu/CNT (Fig. 3c).

Following the elucidation of the reaction thermodynamic pathway by DFT, further investigation is required into the electrophilic and nucleophilic properties during the transformation of key intermediates, as well as the kinetic driving mechanism of this step. It is noteworthy that the reduction of adsorbed *HCOO to *C(OH)_2_ intermediates in the presence of the catalyst, according to calculations of the Fukui function, is more favorable for subsequent coupling reactions with *HCOO. The Fukui functions *f*  ^−^, *f*  ^+^, and *f*  ^0^ are used to describe electrophilic, nucleophilic, and radical reactions, respectively [52]. It has been found that the larger the *f* value of an atom, the more it tends to be the active site for the corresponding type of reaction [53]. Specifically, positions with higher *f*  ^+^ values are more inclined to undergo nucleophilic reactions, showing higher electrophilicity; while positions with higher *f*  ^−^ values are more prone to donate electrons and tend to participate in electrophilic reactions, exhibiting nucleophilicity [54]. The corresponding sequence numbers and Fukui function values for each atom in the two structures are shown in Fig. S48, and Tables S7 and S8. The combination of the above results suggests that C1 in structure 1 is nucleophilic and C1 in structure 2 is electrophilic. Consequently, the C1 in structure 1 is more likely to react electrophilically with adsorbed *HCOO than the C1 in structure 2. Furthermore, the condensed dual descriptor (CDD) was employed to enable more intuitive prediction of reaction sites. Reaction sites with a positive value of CDD are susceptible to attack by nucleophiles, and conversely, reaction sites with a negative value are prone to attack by electrophiles [55]. The CDD value of C1 in structure 1 is negative (−0.0442), further indicating that this structure is more vulnerable to electrophilic reactions. Hydrogen/deuterium (H/D) kinetic isotope effect (KIE) experiments further reveal the promoting effect of hydrogen diffusion in converting *HCOO to *C(OH)_2_. As shown in Fig. S49, based on the catalytic current density of the KOH + H_2_O and KOD + D_2_O electrolyte systems, PdH_0.45_Cu/CNT and PdH_0.68_/CNT are calculated to exhibit the high KIE value, accelerating proton transfer and enabling the electrophilic *HCOO to nucleophilic *C(OH)_2_ conversion process with excellent kinetic performance [56]. This is attributed to lattice hydrogen migration of the PdH*_x_*site, which facilitates the conversion of electrophilic intermediates to nucleophilic intermediates. (Fig. 3d) [40,57]. As shown in Fig. S50, the C–C coupling step is more favorable at the Cu, where the generated electrophilic *HCOO is coupled to nucleophilic *C(OH)_2_ [49]. To evaluate the effect of PdCu alloying on catalyst performance, mixed Pd/Cu catalysts and PdCu intermetallic compound (IMC) were synthesized for comparison. TEM, HAADF-STEM, and EDS elemental mapping images show distinct morphologies and Pd/Cu distribution for PdCu catalysts (Fig. S51a–c). XRD patterns indicate the separate Pd and Cu phases within Pd/Cu mixture catalysts, and confirm the formation of the PdCu IMC (Fig. S51d) [58]. PdCu mixture catalysts and PdCu IMC exhibit significantly lower EtOH FEs and activity than PdCu/CNT within the applied potential range, which proves that the alloy has a decisive influence on activity (Fig. S52a and b). Furthermore, XRD analysis indicates that no alloying occurs between Pd and Cu in the PdCu mixture catalysts during the electrocatalytic process (Fig. S52c). At the PdCu/CNT alloy interface, PdH*_x_* sites preferentially adsorb and activate formate, while Cu sites effectively facilitate the subsequent coupling step [59]. Subsequently, through multiple hydrogenation steps, EtOH is finally generated (Fig. S53).

Moreover, the electrolytic reduction of FA involves multiple hydrogenation steps. As HER is a potential competitive reaction, it is necessary to investigate the competitive relationship between HER and FRR. As shown in Fig. S54a, PdCu/CNT exhibits a reaction order significantly below 1, suggesting concentration-insensitive kinetics attributable to high *HCOO coverage and stably adsorbed *HCOO [60,61]. The reaction rate order of PdCu/CNT is the lowest, indicating the weakest concentration dependence (Fig. S54b–d) [62]. Notably, Pd/CNT has a stronger *HCOO adsorption capacity, but the coverage is lower than that of PdCu/CNT. This can be attributed to excessive adsorption on Pd/CNT, hindering the desorption of intermediates and reducing the availability of effective adsorption sites [63]. The competition between FRR and HER was further explored through DFT calculations (Fig. S55a and b). Compared with PdH_0.45_Cu/CNT and Cu/CNT, H_2_ is more readily formed on PdH_0.68_/CNT [64]. For the Heyrovsky step, the PdH_0.45_Cu/CNT is most favorable for *H generation with the lowest energy barrier (−0.54 eV), while exhibiting the highest energy barrier for H_2_ generation [65]. Competitive adsorption measurements confirmed that *HCOO preferentially occupies active sites on PdH_0.45_Cu/CNT compared to *H, which is consistent with the reaction rate order (Fig. S55c) [60]. Furthermore, Bode plot analysis was conducted to characterize the electrocatalytic reaction kinetics and electron transfer efficiency, thus further elucidating the competitive relationship between HER and FRR. Compared to Pd/CNT and Cu/CNT, the phase angle of PdCu/CNT shifts distinctively toward lower frequencies, indicating that it significantly suppresses the Heyrovsky step of the HER, thereby effectively inhibiting H_2_ production (Fig. S56a) [66]. As shown in Fig. S56b, with the increase in negative potential, the phase angle of PdCu/CNT continuously shifts toward lower frequencies with a notable decrease in frequency. This effect significantly inhibits the Heyrovsky process, making *H more prone to be captured by *HCOO and participate in the subsequent FRR hydrogenation steps [67]. Based on these results, it can be concluded that PdH_0.45_Cu/CNT not only enhances the *H retention but also effectively inhibits the competitive HER [68,69]. The retained *H can serve as proton reservoirs to facilitate the hydrogenation process for FRR. A reasonable explanation for the high *HCOO coverage and the preference for FA to EtOH conversion compared to HER is consistent with the high FE_EtOH_ in the experiment.

**Figure 4.**
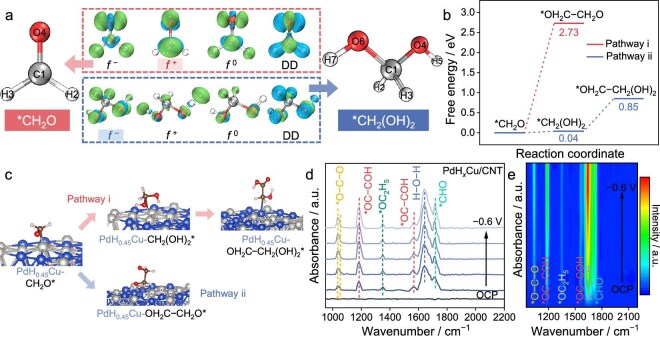
Electrocatalytic formaldehyde self-coupling reaction. (a) Structural diagrams of PdH_0.45_Cu-*CH_2_O and PdH_0.45_Cu-*CH_2_(OH)_2_, along with the corresponding *f*  ^−^, *f*  ^+^, *f*  ^0^, and DD distributions on the Fukui function isosurfaces. The density of the isosurfaces is 0.002 a.u. (b) Free energy diagram for pathways i and ii on PdH_0.45_Cu/CNT. Pathway i is the direct coupling of formaldehyde; pathway ii is the conversion of formaldehyde into a nucleophilic molecule and its coupling with formaldehyde. (c) Optimized structures of the reaction intermediates involved in the pathways i and ii on PdH_0.45_Cu. (d) *In situ* FTIR spectra obtained during the formaldehyde reduction reaction for PdH*_x_*Cu/CNT at different potentials. (e) Equi-contour plot of *in situ* FTIR spectra for PdH*_x_*Cu/CNT.

With the mechanism of the FA system clarified, it is particularly crucial to verify the universality of this catalysis mechanism. Experiments reveal that the mechanism of the PdH_0.45_Cu/CNT promoting electrophile-to-nucleophile conversion to enhance the self-coupling reaction can be utilized for a wide range of electrophilic molecules, such as formaldehyde. In the formaldehyde reduction process, the same catalytic trend as in the FA reduction process can be observed in terms of catalyst ratio changes. PdCu/CNT with a Pd/Cu ratio of 1:1 enhances the self-coupling of formaldehyde with an FE of 81.24% and a formation rate of 1854.1 μmol h^−1^ cm^−2^ at − 0.3 V vs. RHE (Fig. S57a–c). As shown in Fig. S58, adsorbed *CH_2_O is readily converted to nucleophilic *CH_2_(OH)_2_, rather than *C(OH)_2_. Fukui function analysis confirms the electrophilic and nucleophilic properties of the *CH_2_O and *CH_2_(OH)_2_, respectively (Fig. 4a, Tables S9 and S10). Notably, the difference in intermediate formation processes (formate is converted to *C(OH)_2_ via hydrogenation, while *CH_2_(OH)_2_ is formed by the addition of H and OH to formaldehyde) can be attributed to the distinct electrophilicity of the substrates and the differing stabilities of the resulting intermediates (Fig. S59). The adsorbed formaldehyde, exhibiting stronger electrophilicity than formate, is more readily nucleophilically attacked by hydroxyl species generated during the Pd–H formation process, while the Pd–H site synergistically promotes proton transfer to form *CH_2_(OH)_2_ (Fig. 3d) [70]. In contrast, hydroxyl species attack formate energetically unfavorably, as the proposed *C(OH)_3_ intermediate is highly unstable, demonstrating that only the Pd-H site contributes to the electrophilic-to-nucleophilic conversion of the intermediate. DFT calculations indicate that the formation of C–C bonds between the electrophilic *CH_2_O and the nucleophilic *CH_2_(OH)_2_ is favored over the self-coupling of formaldehyde (Fig. 4b and c). To confirm the involvement of formaldehyde and verify important intermediates, *in situ* FTIR spectroscopy was performed (Fig. 4d and e). The peak observed at ∼1715 cm^−1^ can be attributed to the vibration of the *CHO from adsorbed formaldehyde, and the intensity of this peak increases as the applied potential increases (peak assignment in Table S5). The observed symmetric and asymmetric OCO stretching vibration peaks confirm the formation of the key intermediate *CH_2_(OH)_2_. The appearance of *OCCOH and *OC_2_H_5_ peaks indicates the formation of C–C bonds. Moreover, isotope label experiments combined with mass spectrometry analysis further confirm that formaldehyde is the main carbon source and exclude interference from methanol (Fig. S60) [47]. Furthermore, the reaction order significantly below 1 confirms that the intermediates of formaldehyde reduction preferentially occupy active sites and inhibit HER (Fig. S61). Combined with DFT calculations, *H generated from H_2_O dissociation serves as a proton source to participate in hydrogenation rather than promoting HER, which is consistent with the competition mechanism observed in the FA system (Fig. S55).

**Figure 5.**
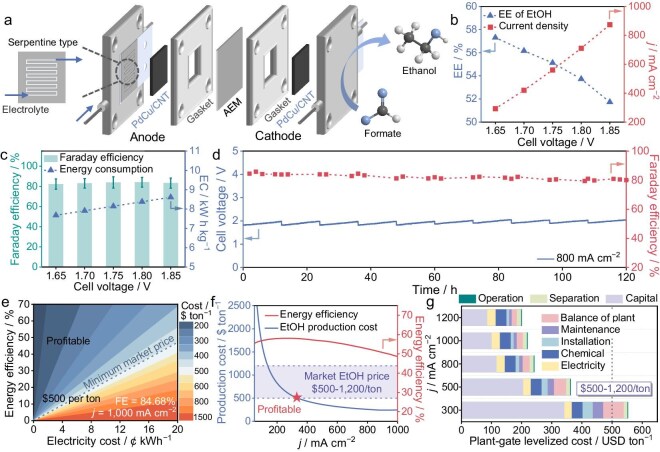
FRR performance in AEM electrolyzer. (a) Schematic illustration of an AEM electrolyzer and serpentine-type flow field. (b) EEs and current densities on PdCu/CNT at different cell voltages. (c) FEs and energy consumption on PdCu/CNT at various cell voltages. (d) Long-term EtOH electrosynthesis at 800 mA cm^−2^ and the corresponding FEs. (e) Plant-gate levelized cost as a function of electricity cost and energy efficiency at 1000 mA cm^−2^. We assume an FE of 84.68% for electrochemical EtOH production. (f) The relationship between electrochemical EtOH production cost and current density, and the corresponding EE at different current densities. (g) TEA of the EtOH electrosynthesis from FA at different current densities. The parameters are based on Table S11.

In view of the outstanding FRR performance of PdCu/CNT, we further assembled an AEM electrolyzer using PdCu/CNT as both anode and cathode catalysts to evaluate the practical application of the catalysts. The cathodic FRR is concurrent with the OER at the anode. The AEM electrolyzer utilizes a serpentine flow field to enhance degassing and mass transfer (Fig. 5a and Fig. S62) [71]. After 12 h of electrolysis, the AEM electrolyzer achieved highly efficient continuous conversion of FA, significantly outperforming the conversion efficiency limitations of traditional H-cell static systems (Fig. S63). With the increase of cell voltage from 1.65 to 1.85 V, the EE of EtOH exhibits a slight downward trend from 57.31% to 51.73%, suggesting that a considerable portion of the electrical energy is efficiently utilized to produce EtOH over a wide voltage window (Fig. 5b). Within the above voltage range, the FEs of EtOH maintain at ∼83.29%, with a high formation rate, while the energy consumption to produce EtOH is only 7.68–8.61 kWh kg^−1^_EtOH_ (Fig. 5c and Fig. S64). As shown in Fig. 5d and Fig. S65a, the PdCu/CNT catalyst realizes a long-term stable operation for 120 h at a current density of 800 mA cm^−2^ with an average FE_EtOH_ and formation rate of 82.26% and 3068.8 μmol h^−1^ cm^−2^, respectively. Additionally, the corresponding single-pass conversion efficiency (SPCE) is also calculated and presented in Fig. S65b. Post-reaction characterization reveals that PdCu/CNT exhibits negligible changes in morphology, elemental distribution, or alloy structure, confirming its excellent stability after long-term operation (Fig. S66). Meanwhile, when argon (Ar) purging is used as a variable to detect collected cathode gas products, its effect on volatile liquid products is negligible (Fig. S67). The cathodic H_2_ source was identified by combining isotope labeling with differential electrochemical mass spectroscopy (DEMS), using a 1.0 M KOD + 1.0 M FA electrolyte in D_2_O (Fig. S68). The DEMS results showed a predominant *m/z* = 4 signal (D_2_). In contrast, *m/z* = 3 (HD) or *m/z* = 2 (H_2_) signals are negligible, confirming H_2_ generation primarily from water reduction rather than FA decomposition. Quantitative analysis of EtOH content at the cathode and anode, as well as possible EtOH anodic oxidation products (acetic acid and CO_2_) in a 120 h stability test, indicates that crossover effects are negligible (<1.5%). Only trace amounts of oxidation by-products are detected (Fig. S69). Moreover, TEA was conducted to assess the commercial viability of the EtOH electrosynthesis strategy. TEA is conducted based on the model shown in Fig. S70 and the parameters shown in Table S11. As shown in Fig. 5e and Fig. S71a, TEA results indicate that the proposed EtOH production system exhibits resistance to fluctuations in electricity prices at industrial current densities. The projected production costs remain well below the current lowest price in the EtOH market ($500 ton^−1^) even under extreme electricity price fluctuations (Fig. S71b). TEA further confirms the profitability of electrocatalytic EtOH production by reduced FA costs (Fig. S72). Industrial by-product FA (for example, from cellulose hydrolysis/petrochemical refining) is priced below market value. Further cost reduction is achievable via novel FA production, such as waste-derived and CO_2_ hydrogenation electrolysis [72]. As shown in Fig. 5f and g, when the by-product FA is used as raw material, the plant-gate EtOH production cost falls below the lowest market price at 363.9 mA cm^−2^, indicating the economic feasibility of this process. Furthermore, electrocatalytic conversion of FA waste into EtOH enables efficient resource utilization and a circular economy, filling the huge market gap for EtOH (global demand exceeding 100 billion gallons per year by 2030) and achieving waste resource utilization [3]. Moreover, the EtOH production cost via the formaldehyde pathway was also evaluated, revealing that it remains significantly below the lowest market price (Fig. S73a). The system is equally capable of withstanding electricity price volatility, demonstrating both the economic viability and industrial application potential of this route (Fig. S73b and c).

## CONCLUSION

This work overcomes the long-standing challenge of conventional gaseous C_1_ feedstocks in simultaneously achieving high current density and selectivity during electrocatalytic C–C coupling for multi-carbon product synthesis, enabling a promising direction for the industrial production of EtOH via electrolysis. The high FE_EtOH_ (84.68%) can be achieved on PdCu/CNT with the EtOH formation rate reaching 1970.1 μmol h^−1^ cm^−2^ at −0.4 V vs. RHE with a current density of 500 mA cm^−2^. Experimental and DFT results suggest that the *in situ*-generated PdH*_x_* site boosts the conversion of intermediates from electrophile to nucleophile due to the effect of active *H. Cu contributes to the formation of C–C bonds between electrophilic *HCOO and nucleophilic *C(OH)_2_, thereby guaranteeing the generation of multi-carbon products. This PdH_0.45_Cu/CNT-promoted C–C coupling mechanism also applies to promoting the coupling of formaldehyde molecules to generate EtOH. In practical application, the PdCu/CNT achieves 120 h of continuous stable operation at industrial-level current densities in an AEM electrolyzer. TEA indicates the EtOH production cost of this system is only 242 USD ton^−1^, far below the minimum market price of 500 USD ton^−1^, demonstrating industrial profitability potential. This study reveals a novel asymmetric HCOO-C(OH)_2_ coupling pathway, providing a breakthrough strategy for the efficient transformation of C_1_ molecules into high-value-added multi-carbon products via C–C coupling. We believe that the technological approach developed in this study can be coupled with efficient electrolytic reduction of CO_2_ to produce FA, substantially reducing EtOH production costs and demonstrating broad prospects for large-scale application.

## Supplementary Material

nwag220_Supplemental_File
