# Supplementary material for: Industrial electrocatalytic C–C coupling reaction of C1 liquid molecules for efficient ethanol synthesis
Source: Natl Sci Rev. 2026 Apr 10;13(12):nwag220. doi: 10.1093/nsr/nwag220 (PMC13321124; doi:10.1093/nsr/nwag220)
Supplement: nwag220_Supplemental_File [file nwag220_supplemental_file.pdf]

Supporting Information

Industrial electrocatalytic C-C coupling reaction of C_1 liquid molecules for efficient ethanol synthesis

Jiani Han [a,b], Yaodong Yu [a], G. A. Bagliuk [c], Jianping Lai [a,*] and Lei Wang [a,*]

[a] State Key Laboratory Base of Eco-Chemical Engineering, Ministry of Education, International Science and Technology Cooperation Base of Eco-chemical Engineering and Green Manufacturing, College of Chemistry and Molecular Engineering, Qingdao University of Science and Technology, Qingdao 266042, P. R. China.

[b] Shandong Engineering Research Center for Marine Environment Corrosion and Safety Protection, College of Environment and Safety Engineering, Qingdao University of Science and Technology, Qingdao 266042, P. R. China.

[c] Frantsevich Institute for Problems of Materials Science, National Academy of Sciences of Ukraine, Kyiv 02000, Ukraine.

Experimental Section

Materials. Copper (II) acetylacetonate ($C_{10}H_{14}CuO_4$, 97%, Aladdin), palladium (II) acetylacetonate ($C_{10}H_{14}O_4Pd$, 99%, Aladdin), formate (CH_2O_2, ≥99%, Aladdin), ethanol absolute (C_2H_6O, 99.5%, Hushi), carboxylic multi-walled carbon nanotubes (>95%, Macklin), potassium hydroxide (KOH, 95%, Macklin), dimethyl sulfoxide (DMSO, 99.9%, Aladdin), deuterium oxide (D_2O, 99.9%, Aladdin), formic acid-^{13}C solution ($H^{13}CO_2H$, 95%, Sigma), formaldehyde-^{13}C solution ($^{13}CH_2O$, 99%, Aladdin), carbon paper (Hesen HCP020N), anion exchange membrane (AEM, X37-50 grade T). The deionized water (18 MΩ/cm) used in all experiments was prepared through ultrapure purification systems (Aqua Solutions).

Synthesis of Pd_xCu/CNT electrocatalysts. All catalysts were synthesized using a solvent-free

microwave method. In the case of PdCu/CNT, for example, $Pd(acac)_2$ (10 mg), $Cu(acac)_2$ (8.6 mg), and CNT (20 mg) were mixed in a mortar and ground evenly. The mixture was put into a 10 ml quartz bottle, and Ar was poured into the bottle to fill it with Ar. Then it was placed in a household microwave oven (Midea, PM2001) and reacted for 30 s with a power of 1 kW. The synthesis is initiated at room temperature and normal atmospheric pressure. The reaction mixture was then washed and centrifuged with acetone. Finally, the product was dried overnight in a 60 ˚C oven to obtain a black powder. A series of Pd_xCu/CNT electrocatalysts were synthesized, with different molar ratios of Pd: Cu (0.8:1, 0.9:1, 1.1:1, and 1.2:1 for $Pd_{0.8}Cu$/CNT, $Pd_{0.9}Cu$/CNT, $Pd_{1.1}Cu$/CNT, and $Pd_{1.2}Cu$/CNT, respectively).

Synthesis of Pd/CNT and Cu/CNT electrocatalyst. Pd/CNT was synthesized in the same steps as mentioned above, with the difference of the absence of the addition of $Cu(acac)_2$. Only $Cu(acac)_2$ (8.6 mg) and CNT (20 mg) were mixed in a mortar and ground evenly for Cu/CNT. The subsequent steps were the same as described in the above method.

Synthesis of PdCu nanoparticles (NPs). Na_2PdCl_4 (0.05 mmol), $Cu(acac)_2$ (0.05 mmol), ribose (15 mg), 1-octadecene (2.5 mL), and oleylamine (2.5 mL) were added to a 35 mL vial. After sealing, the mixture was ultrasonicated for 30 minutes. The resulting homogeneous mixture was then heated in an oil bath from room temperature to 180°C, maintained for 3 h, and cooled to room temperature. The colloidal product was collected by centrifugation and washed three times.

Synthesis of PdCu intermetallic compound (IMC). PdCu NPs and commercial carbon were mixed in 10 mL cyclohexane and sonicated for 1 h to deposit NPs on carbon. The products were separated by centrifugation and washed three times. The obtained PdCu/C was subsequently annealed at 375 °C for 1 h under a 10% H_2/90% Ar atmosphere.

Characterization. Scanning electron microscopy (SEM) images were obtained by Hitachi S-8200. The transmission electron microscope (TEM) and high-resolution TEM (HRTEM) of the catalyst were tested using FEI Tecnai-G2 F30 at an accelerating voltage of 80 KV. The composition and

concentration of catalysts were analyzed by inductively coupled plasma-atomic emission spectroscopy (ICP-AES, Varian 710-ES) on a Prodigy. The samples for ICP-AES were pre-treated in a microwave digestion & extraction system (MARS Xpress). X-ray diffraction (XRD) analysis at a scanning rate of 1° min^{-1} in the 2θ range of 5 to 90° was used to examine the composition of the as-synthesized samples on an X'Pert-Pro MPD diffractometer (Netherlands PANalytical) operating at 40 KV and 40 mA with Cu Kα radiation, and then the results were compared with MDI jade. Electrochemical characterization was carried out on a CHI 760E workstation (Shanghai Chenhua Instrument Corporation, China) and a Gamry electrochemical workstation (Gamry 3000). The Axis Supra spectrometer was used for X-ray photoelectron spectroscopy (XPS) analysis by using a monochromatic Al Kα source (15 mA, 14 kV). All spectra were calibrated using the C 1s main peak (graphitic C=C peak) as an internal standard, with its binding energy referenced to 284.8 eV. The calibrated data were subsequently analyzed using Casa XPS software to determine the elemental valence states of the catalyst. Gas products were analyzed by gas chromatography (GC-7890B, Agilent, USA) equipped with a thermal conductivity detector (TCD) and flame ionization detector (FID). Ar (99.99%) was used as a carrier gas. ^{1}H nuclear magnetic resonance (NMR) spectra were recorded on a BRUKER AVANCE NEO 400 MHz liquid NMR spectrometer in the indicated solvents. The *in situ* Raman spectrum was obtained by the Invia Qontor with He/Ne laser wavelengths of 633 nm. Fourier transform infrared (FTIR) spectrometer (Nicolet IS50, Thermo Fisher Scientific) and ATR were used for infrared spectroscopy.

Electrochemical measurements. Electrochemical measurements were performed in a conventional three-electrode system, with a platinum wire as the counter electrode and a saturated calomel electrode (SCE) as the reference electrode, while the prepared catalysts were used as the working electrode. The electrolyte was saturated with Ar prior to electrochemical measurements in a three-electrode system. All potentials reported in this work are corrected using reversible hydrogen electrodes (RHE), and all electrochemical curves were collected by the workstation and underwent

95% iR compensation. All potentials measured in 1.0 M KOH with or without 1.0 M FA solution were converted to values about RHE according to the equation:

$$E(RHE) = E(SCE) + 0.244\ V + 0.0592 \times pH$$

The catalyst ink for the working electrode was prepared by dispersing 2 mg of the as-prepared sample in a mixed solution (30 μL Nafion (0.5 wt%), 500 μL isopropanol, and 470 μL water), followed by sonication for 30 min to obtain a homogeneous catalyst ink with a concentration of 2 mg mL^{-1}. Then 100 μL of catalyst ink was dropped onto a 1 cm × 1 cm carbon paper (CP). A mass loading of 0.2 mg cm^{-2} was used for the electrochemical study. All experiments were carried out at room temperature (25°C). Linear sweep voltammetry (LSV) was conducted at a scan rate of 5 mV/s. Durability testing was carried out in 1.0 M KOH + 1.0 M FA solution using chronoamperometry. Next, to further assess the stability of the catalyst, LSV was measured after 10,000 cycles of cyclic voltammetry (CV). The electrochemical double-layer capacitance (C_{dl}) measurements were calculated from CV measurements, which were collected in a non-faradaic region with various scan rates ranging from 20 to 100 mV/s at potentials between 0.41 V and 0.51 V vs. RHE in 1.0 M KOH + 1.0 M FA under Ar. The formula for calculating C_{dl} (mF cm^{-2}) is as follows:

$$C_{dl} = (j_a - j_c)/(2 \bullet v) = (j_a + |j_c|)/(2 \bullet v) = \Delta j/(2 \bullet v)$$

In which j_a and j_c is the anodic and cathodic voltammetric current density, respectively, recorded at the middle of the selected potential range, and v is the scan rate. To estimate electrochemically active surface area (ECSA) values of the materials, C_{dl} was measured using a simple cyclic voltammetry method. The potential was selected beyond the possible Faraday area of the material to record the CV at different scan rates of 20 ~ 100 mV/s. The capacitive current density $\Delta j/2$ was linearly correlated with the scan rate, and the C_{dl} was calculated from the slope of these straight lines. The ECSA obtained by means of the double-layer capacitance is calculated as follows:

$$ECSA = \frac{C_{dl} \times S}{C_s}$$

$$C_s = 0.04\, mF\ cm^{-2}$$

where S in the equation is the geometric surface area of the working electrode (1.0 cm^2). Cs is the double-layer capacitance of an ideally flat electrode, with a value of 0.04 mF cm^{-2} (a common reference value in transition metal catalyst ECSA calculations) [1]. Electrochemical impedance spectroscopy (EIS) was performed on the PAR-STAT 2273 test system with a frequency range of 0.1 Hz to 100 kHz in the same solution.

The two-electrode electrolysis is carried out on an H-cell electrochemical workstation with an anion exchange membrane. For the OER/FRR (OER: oxygen evolution reaction, FRR: FA reduction reaction) two-electrode electrolyzer, PdCu/CNT electrocatalysts were employed as the anode and cathode, 1.0 M KOH + 1.0 M FA was used as the cathodic electrolyte, and 1.0 M KOH was used as the anodic electrolyte. Chronoamperometric tests were then conducted at different potentials, and Ar was continuously fed into the anode cell during the experiments.

Product analysis. Gas products were analyzed using gas chromatography (GC-7890B) equipped with a thermal conductivity detector (TCD) and flame ionization detector (FID). Ar (99.99%) was used as a carrier gas. The identification and quantification of glutaric acid were determined from ^{1}H NMR using a calibration curve with dimethyl sulfoxide (DMSO) as an internal standard. Before and after electrolysis 500 μL of electrolyte was acidified by 20 μL of HCl (37%) and then added to 100 μL of D_2O. ^{1}H NMR was recorded using the water suppression method.

Calculation of Faradaic efficiency (FE) and formation rate. The FE for product electrosynthesis was defined as the amount of electric charge used for generating the product divided by the total charge passed through the electrodes during the electrolysis. The FE was calculated on the basis of the following equation:

$$FE\ (\%) = \frac{nF \times N}{Q_{total\ charge\ passed}} \times 100$$

where n is the number of electrons transferred for each product molecule, F is Faraday's constant

(96,485 C mol^{-1}), N is the mole number of products, and Q is the total passed charge.

The formation rate of EtOH was calculated using the following equation:

$$Formation\ rate\ (EtOH) = \frac{C_{EtOH} \times V}{t \times m_{catalyst}}$$

In which C_{EtOH} is the measured concentration of EtOH, V is the volume of the cathodic reaction electrolyte, t is the time for which the potential was applied, and $m_{catalyst}$ is the mass loading of the catalyst.

Calculation of energy efficiencies (EE). The half-cell and full-cell EE are calculated as follows:

$$Half - cell\ EE_{EtOH} = \frac{(1.23 - E_{EtOH}) \times FE_{EtOH}}{(1.23 - E_{app})}$$

$$Full - cell\ EE_{EtOH} = \frac{(1.23 - E_{EtOH}) \times FE_{EtOH}}{E_{cell}}$$

Where E_{EtOH} is the thermodynamic potential (vs. RHE) of EtOH. E_{app} is the applied potential (vs. RHE) in the three-electrode setup. E_{cell} is the cell voltage.

Calculation of the lattice hydrogen atoms.

$$d=\lambda/(2\sin\theta)$$

$$d=\alpha_{\mathrm{pdH}}\sqrt{h^2 + k^2 + l^2}$$

$$\frac{3 \times (\alpha_{\mathrm{pdH}} - \alpha_{\mathrm{pd}})}{x\alpha_{\mathrm{pdH}}} = 0.19$$

Where λ is the X-ray wavelength (0.154 nm), θ is the angle in X-ray diffraction, is the facet of Pd, αis the lattice parameter, x is the number of hydrogen atoms [2].

***In situ* Raman spectroscopy measurements.** *In situ* Raman experiments were performed in 1.0 M KOH + 1.0 M FA electrolytes. The *in situ* electrolytic cell is composed of a working electrode at the bottom, a platinum wire as the counter electrode, and an SCE as the reference electrode. The working

station is the same as the electrochemical test station. The lens used was a confocal Raman microscope with a 60× objective (inVia Qontor, Renishaw). The laser wavelength used for all Raman tests was 532 nm.

Computational method. The study used Vienna Ab-initio Simulation Package (VASP) to perform density functional theory calculations with projector augmented wave. The electron spin-polarization was included in all calculations. The Perdew-Burke-Ernzerhof functional was employed for exchange-correlation effects, while DFT+D3 was used for handling weak interactions. The cut-off energy for the plane-wave basis was 450 eV. K-points were 2*2*1 in the Brillouin zone. 15 Å of layer vacuum was applied at the Z-axis of slab models to avoid the Periodic effect. Energy and maximum stress converged to 10-5 eV and 0.02 eV $Å^{-1}$, respectively.

Calculation of Fukui function. The Fukui function is defined as the variation in the electronic density due to the change in the electron number at fixed external potential:

$$f(r) = \left[\frac{\delta\mu}{\delta\upsilon(r)}\right]_N = \left[\frac{\partial\rho(r)}{\partial N}\right]_{\upsilon(r)}$$

where μ is the chemical potential, N is the number of electrons in the system, and υ(r) represents the attraction potential of the nucleus to the electrons. Since the partial derivatives of the electron density with respect to N are discontinuous when N is an integer, the Fukui function is often approximated using finite differences:

$$f^-(r) = \rho_N(r) - \rho_{N-1}(r) \approx \rho^{HOMO}(r)$$

$$f^+(r) = \rho_{N+1}(r) - \rho_N(r) \approx \rho^{LUMO}(r)$$

$$f^0(r) = [f^-(r) + f^+(r)]/2 \approx [\rho^{HOMO}(r) + \rho^{LUMO}(r)]/2$$

In the derivation of the above equation, the approximate method of frozen orbits is used. Where $\rho_N(r)$, $\rho_{N-1}(r)$ and $\rho_{N+1}(r)$ represents the electron densities of the system in the pristine state (N electrons), ionized off one electron state (N-1 electrons) and combined one electron state (N+1 electrons),

respectively; $\rho^{HOMO}(r)$ is the highest occupied molecular orbital (HOMO) of the molecule and $\rho^{LUMO}(r)$ is the lowest unoccupied molecular orbital of the molecule. The three Fukui functions in the equation, i.e., $f^{-}(r)$, $f^{+}(r)$, and $f^{0}(r)$, are used to predict electrophilic, nucleophilic, and radical reactions, respectively.

Calculation of dual descriptor. The dual descriptor (DD) is defined as:

$$f(r) = \left[\frac{\delta\eta}{\delta v(r)}\right]_N = \left[\frac{\partial f(r)}{\partial N}\right]_{v(r)}$$

Where η is the chemical hardness of the system. Through the finite difference method, the specific formula of the DD can be obtained:

$$f^{(2)}(r) = f^{+}(r) - f^{-}(r) \approx \rho^{LUMO}(r) - \rho^{HOMO}(r)$$

The contraction of a Fukui function to an atom then results in a condensed dual descriptor (CDD), which is defined as follows:

$$f_A^{(2)} = f_A^{+} - f_A^{-} = 2q_N^A - q_{N+1}^A - q_{N-1}^A$$

Where q^A is the charge of atom A in the molecule. It is worth noting that the atomic charge is not an observable quantity, therefore, the Hirshfeld method was used to calculate the atomic charge in this experiment.

Reaction order rate calculations. The FA reduction rate order was calculated by fitting the slope of the ln *j* (current density) against the ln $[HCOO^-]$ ($HCOO^-$ concentration) at different potentials. The calculation was based on the following equation:

$$2HCOO^- + 7H_2O + 8e^- \rightarrow CH_3CH_2OH + 10\ OH^- \quad c = k\,[HCOO^-]^a\,[H_2O]^b$$

where *c* is the reaction rate of formate, *k* is the reaction constant, a and b are the reaction order of formate and H_2O, respectively. $[HCOO^-]$ and $[H_2O]$ are the concentrations of formate and water. Since $[H_2O] >> [HCOO^-]$, $[H_2O]^b$ can be regarded as a constant (K). Therefore, the equation can be

rewritten as: ln c = K + a ln [$HCOO^-$].

Since the reaction rate c is proportional to j, the equation can be rewritten as: ln j = K + a ln [$HCOO^-$]. Accordingly, the reaction rate order can be calculated by fitting the slope of the ln j against the ln [$HCOO^-$].

Single-pass conversion efficiency (SPCE) calculation. The SPCE is calculated using the following formula: $SPCE = \frac{n_{consumed\ substrate}}{n_{initial\ substrate}} \times 100\%$, where n represents the amount of substrate [3].

Energy consumption calculation. The energy consumption of an AEM electrolyzer is calculated by the equation: Energy consumption $= \frac{I_{cell} \times U_{cell}}{m_{EtOH}} t$

Where I_{cell} is the delivered current (A), t is the operation time (h), m_{EtOH} is the mass of EtOH produced in a t duration, which can be calculated by Faraday's laws of electrolysis: $m_{EtOH} = \frac{I_{cell} \times t}{z * F} M_{EtOH}$

z is the number of electrons transferred to produce one EtOH molecule, M_{EtOH} relative molecular mass (46.07 g mol^{-1}).

Preparation of AEM electrolyzer. The AEM electrolyzer was composed of the cathode, anode, and commercial AEM membrane. The membrane was immersed in 1.0 M KOH solution for at least 24 h prior to being employed as an electrolyte. The cathode and anode were prepared by air-spraying catalyst ink onto the carbon paper (working electrode area: 1.0 cm × 1.0 cm). Subsequently, the cathode and anode were sandwiched with an anion exchange membrane and pressed to prepare an AEM water electrolyzer device. 1.0 M KOH solution was used as an anodic electrolyte, and 1.0 M KOH + 1.0 M FA solution was used as a cathodic electrolyte. The stability of the AEM electrolyzer was evaluated at 800 mA cm^{-2} for 120 h.

Assumptions for Techno-economic analysis. We conducted a techno-economic study to assess the feasibility of producing EtOH from FA using renewable electricity. The relevant parameters and

references are listed in **Table S11**. In this model, the plant-gate levelized cost under consideration included the input chemicals, electricity, catalyst, membrane, electrolyzer, installation, separation, maintenance, operation, and balance of the plant. The following is a list of assumptions used for the calculations.

1. The capacity of the plant is 1 ton of EtOH per day.

2. Installation cost is assumed to be 10% of the total capital cost.

3. Separation cost is considered to be 50% of the cost of electricity.

4. Maintenance cost is 10% of capital cost.

5. Operating costs are 10% of electricity costs and represent additional operating costs associated with plant operations, such as labor costs.

6. The balance of plant is 20% of the total capital cost.

7. The lifespan of the electrolyzer is estimated to be 30 years.

8. Assuming that the factory operates 24 hours per day, with a capacity factor of 0.9.

Cost components of techno-economic analysis.

1. Electrolyzer cost

$$Area\ of\ electrolyzer = \frac{Total\ current\ needed\ (A)}{Operating\ current\ density\ (A/m^2)}$$

$$Total\ current\ needed\ (A) = \frac{Plant\ capacity\ (ton/day) \times No.e\ transferred \times F\ constant\ (C/mol)}{Product\ molar\ mass\ (g/mol) \times 24\ (h/day) \times 3600(s/h) \times FE(\%)}$$

$$Electrolyzer\ cost\ (\$/ton) = \frac{Area\ of\ electrolyzer(m^2) \times Electrolyzer\ cost(\$/m^2)}{Capital\ factor \times Electrolyzer\ lifetime(year) \times 365(day/year) \times EtOH\ Production(ton/day)}$$

2. Electricity cost

$$Electricity\ consumption(kWh/h)$$

$$= \frac{Total\ current\ needed\ (A) \times Cell\ voltage(V) \times 24(h/day)}{Plant\ capacity\ (ton/day) \times 1000(W/kW)}$$

$$Electricity\ cost(\$/ton) = Electricity\ consumption\left(\frac{kWh}{ton}\right) \times Electricity\ cost(\$/kWh)$$

3. Installation cost $Installation\ cost = Capital\ cost \times 10\%$

4. Separation cost $Separation\ cost = Electricity\ cost \times 50\%$

5. Maintenance cost $Maintenance\ cost = Capital\ cost \times 10\%$

6. Operation cost $Operation\ cost = Electricity\ cost \times 10\%$

7. Balance of plant $Balance\ of\ plant = Capital\ cost \times 20\%$

Figures

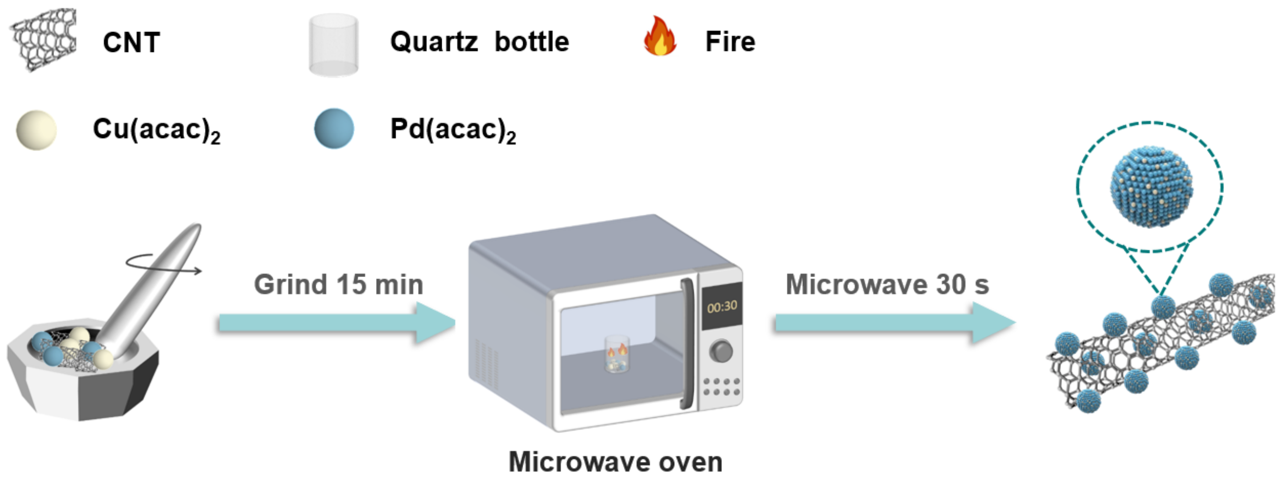

Fig. S1 Schematic illustration of the PdCu/CNT synthesis process via the solvent-free microwave method.

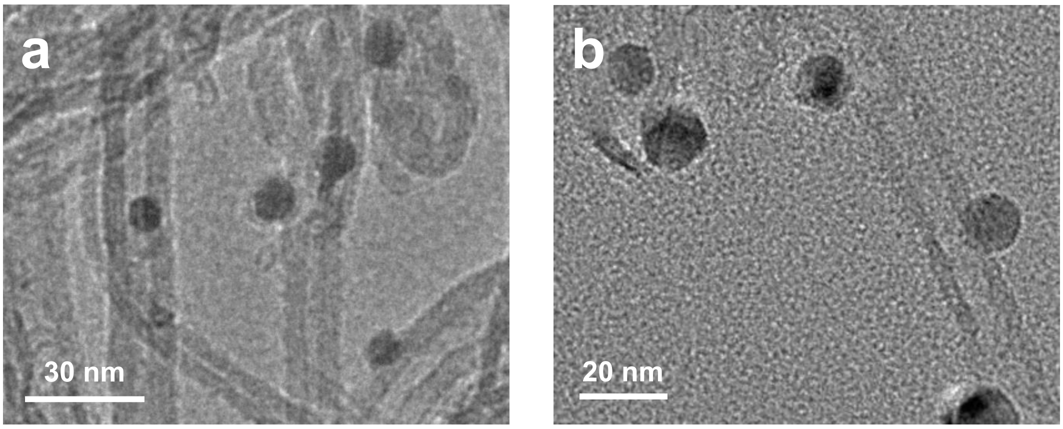

Fig. S2 TEM images of as-synthesized (a) Pd/CNT and (b) Cu/CNT.

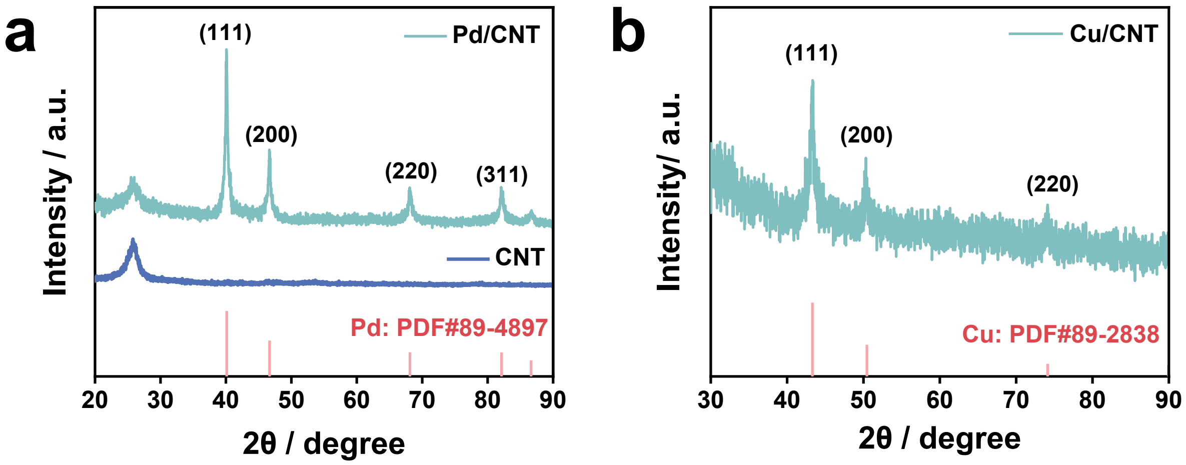

Fig. S3 XRD patterns of (a) Pd/CNT, CNT, and (b) Cu/CNT.

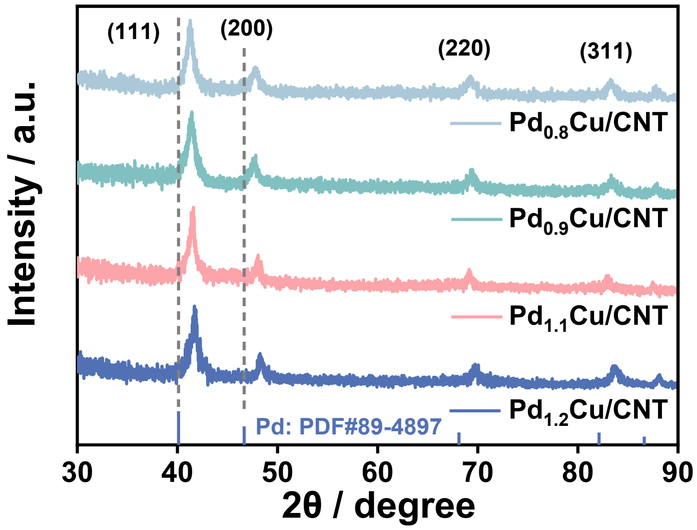

Fig. S4 XRD patterns of PdCu-based catalysts with different Pd/Cu ratios.

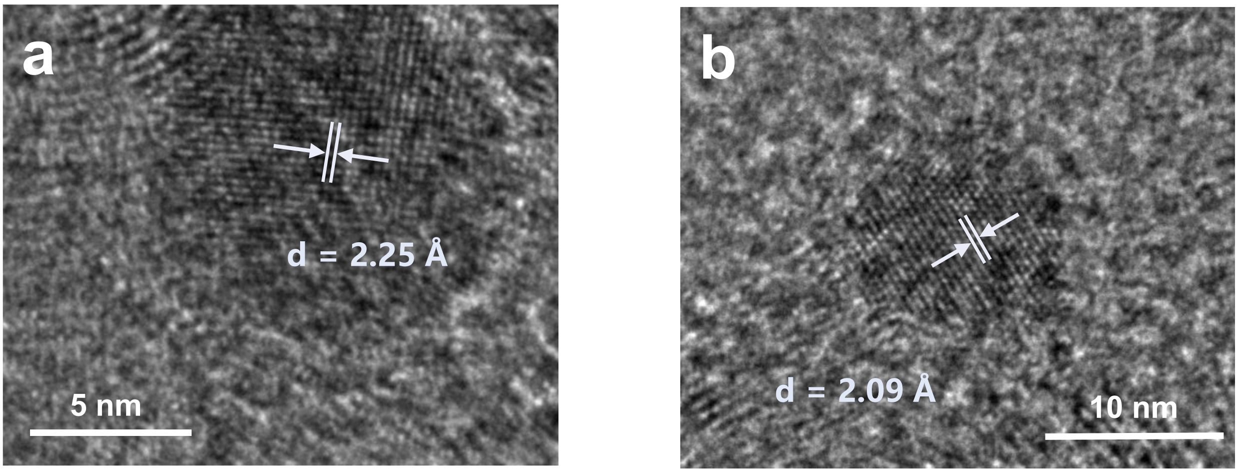

Fig. S5 HR-TEM images of (a) Pd/CNT and (b) Cu/CNT.

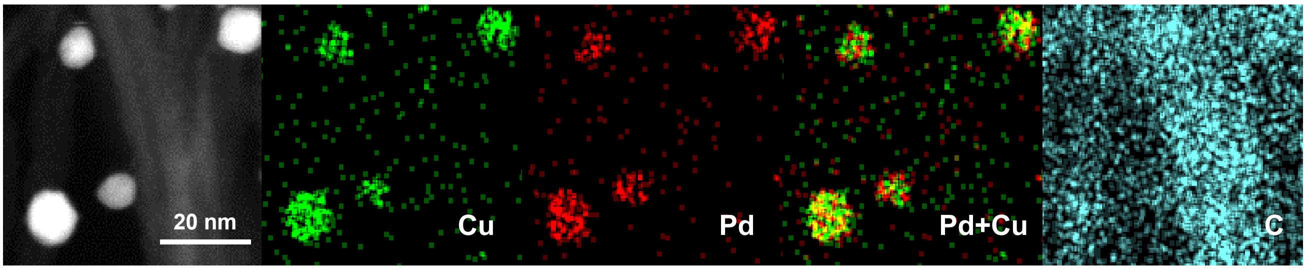

Fig. S6 HAADF-TEM image, and the corresponding EDS elemental mapping of PdCu/CNT.

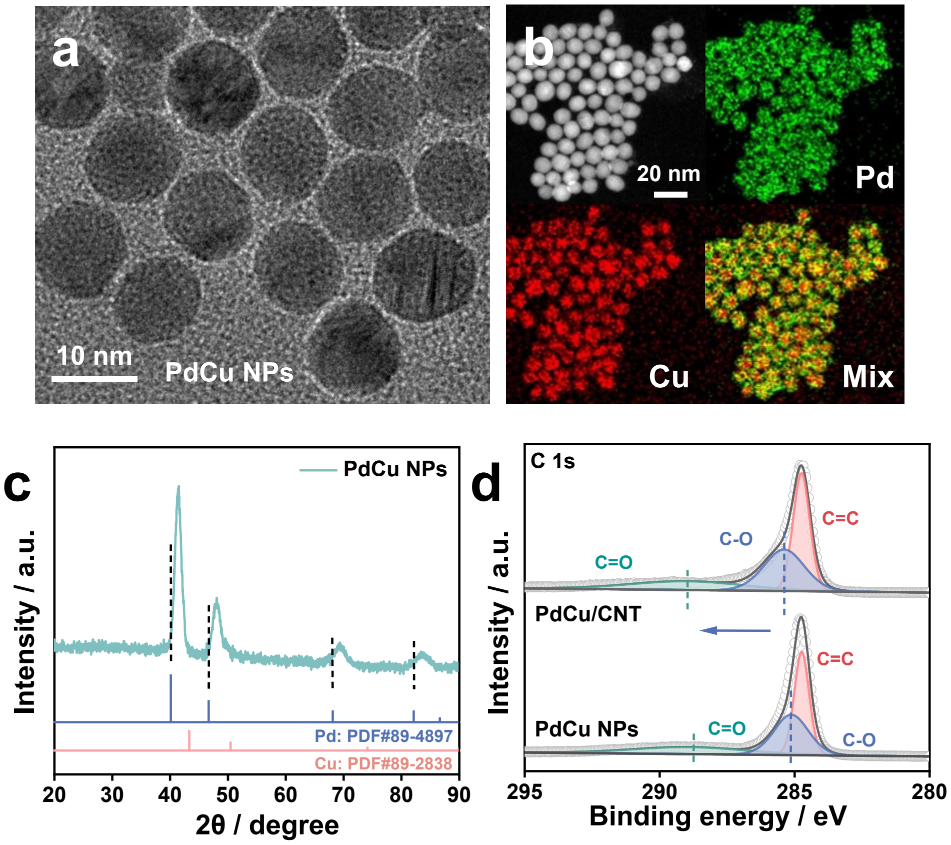

Fig. S7 (a) TEM, (b) HAADF-STEM, and the corresponding EDS-mapping images of PdCu NPs (without CNT carriers). (c) XRD pattern for PdCu NPs. (d) C 1s XPS spectra of PdCu/CNT and PdCu NPs.

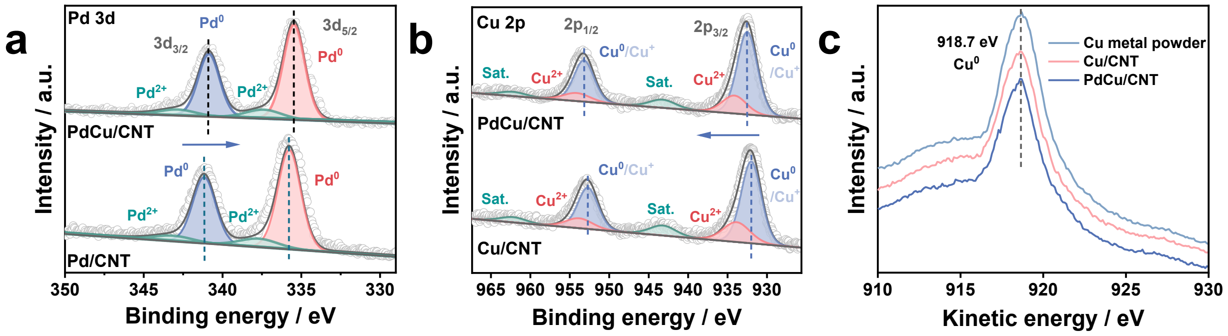

Fig. S8 (a) Pd 3d XPS spectra for PdCu/CNT and Pd/CNT. (b) Cu 2p XPS spectra for PdCu/CNT and Cu/CNT. (c) Cu LMM Auger spectra for PdCu/CNT, Cu/CNT, and Cu metal powder.

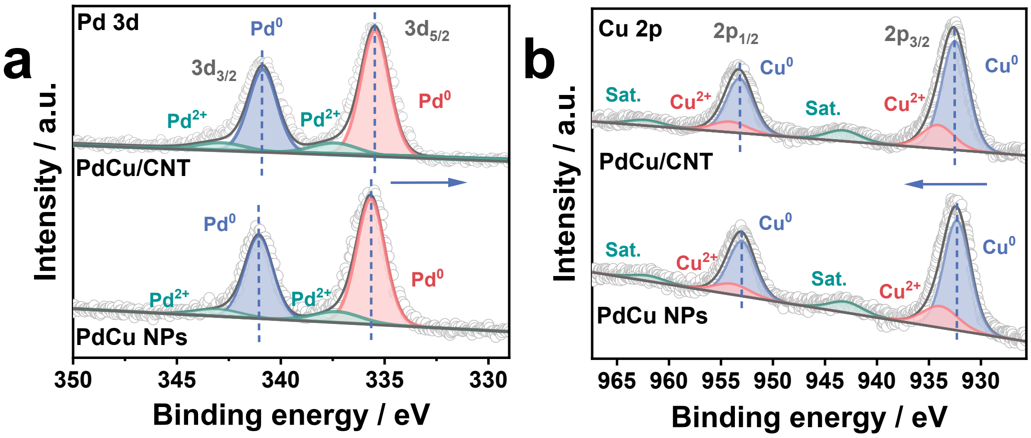

Fig. S9 (a) Pd 3d, and (b) Cu 2p XPS spectra of PdCu/CNT and PdCu NPs.

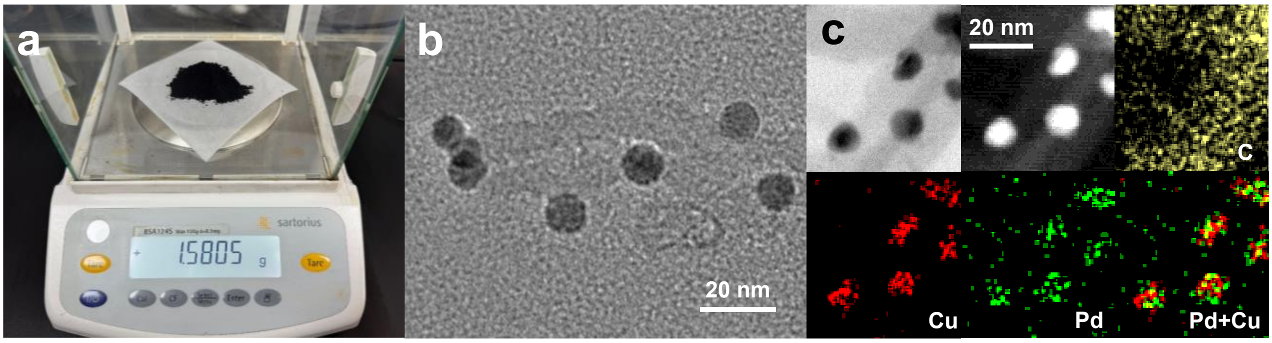

Fig. S10 Large-scale production of PdCu/CNT. (a) Photograph of large-scale synthesized catalysts. (b) TEM, (c) high-angle-annular bright- and dark-field STEMs, and the corresponding EDS-mapping images of PdCu/CNT (large-scale production).

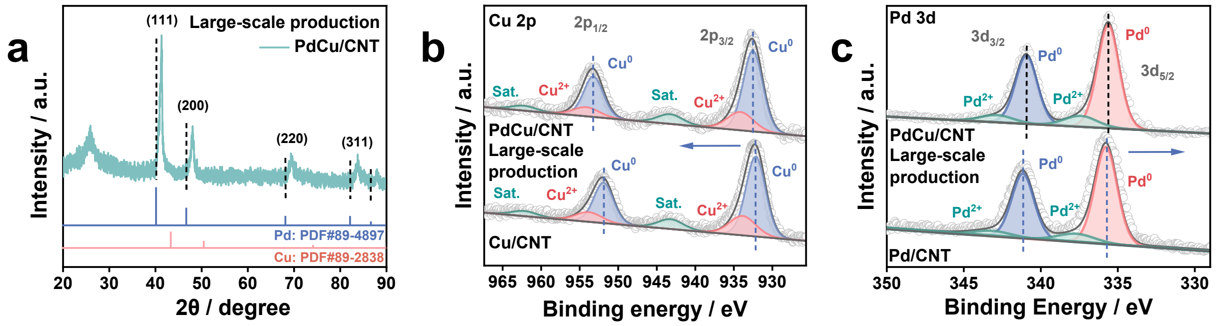

Fig. S11 Large-scale production of PdCu/CNT. (a) XRD pattern of the large-scale production of PdCu/CNT. XPS spectra in the (b) Cu 2p and (c) Pd 3d region of PdCu/CNT.

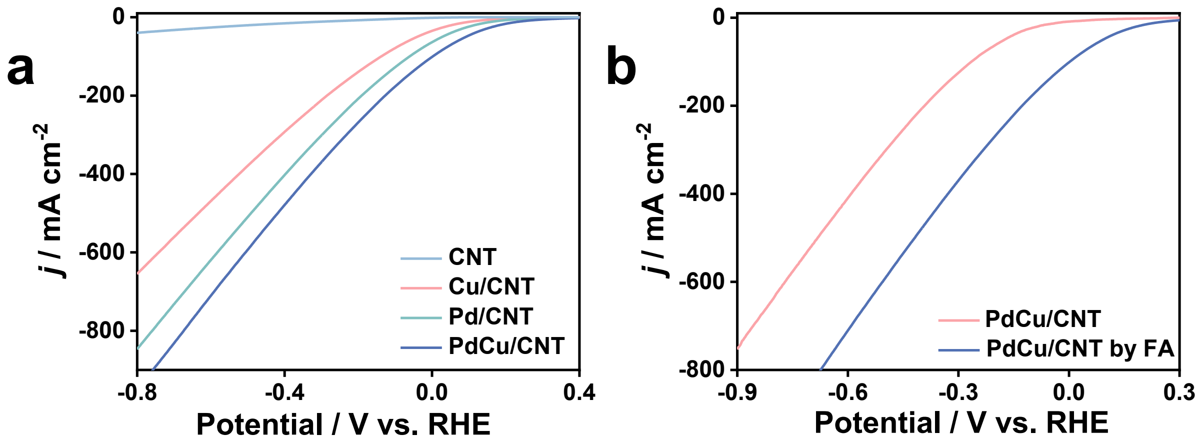

Fig. S12 (a) Comparison of polarization curves over PdCu/CNT, Pd/CNT, Cu/CNT, and CNT electrodes in 1.0 M KOH + 1.0 M FA electrolyte. (b) Polarization curves of PdCu/CNT in 1.0 M KOH and 1.0 M KOH + 1.0 M FA electrolyte.

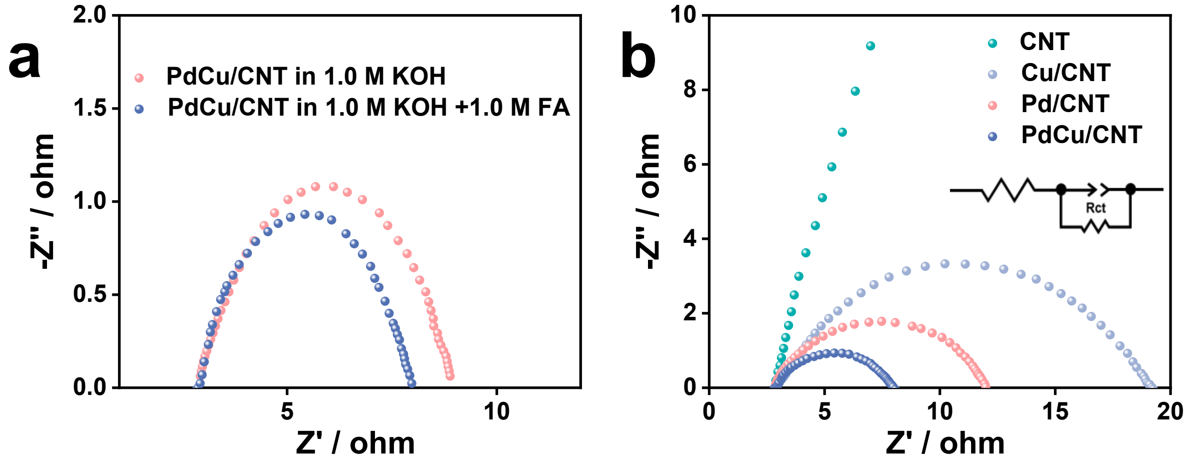

Fig. S13 (a) Comparison of EIS curves for PdCu/CNT in different electrolytes. (b) EIS curves for PdCu/CNT, Pd/CNT, Cu/CNT, and CNT in 1.0 M KOH + 1.0 M FA electrolyte.

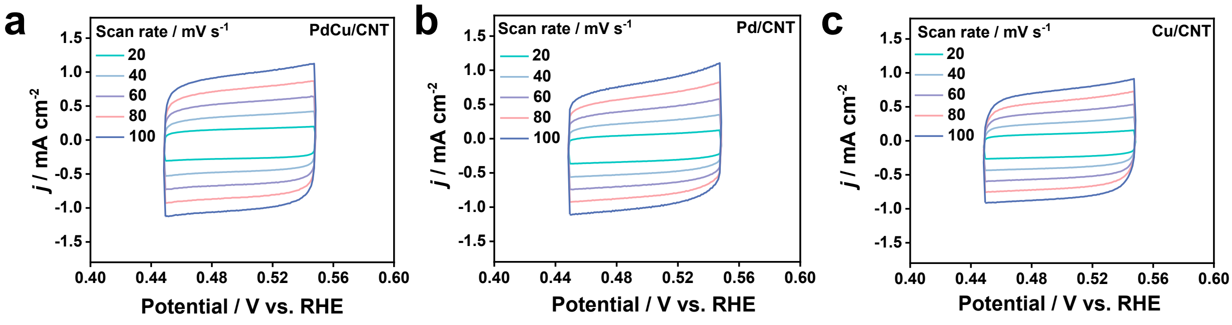

Fig. S14 CV curves for (a) PdCu/CNT, (b) Pd/CNT, and (c) Cu/CNT electrodes were collected in a non-Faradaic region with various scan rates ranging from 20 to 100 mV s^{-1} in 1.0 M KOH + 1.0 M FA electrolyte under Ar.

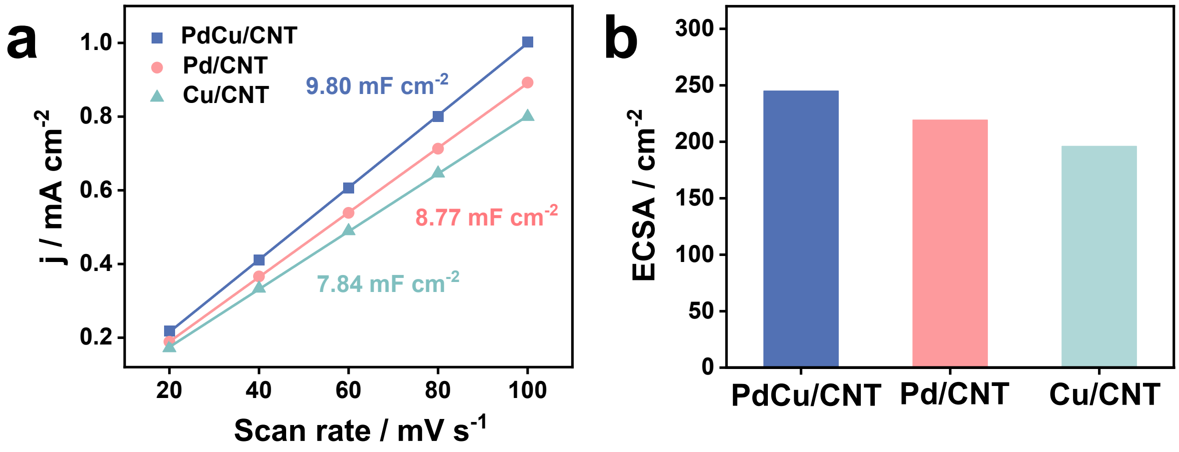

Fig. S15 The comparison of (a) C_{dl} and (b) ECSA for PdCu/CNT, Pd/CNT, and Cu/CNT.

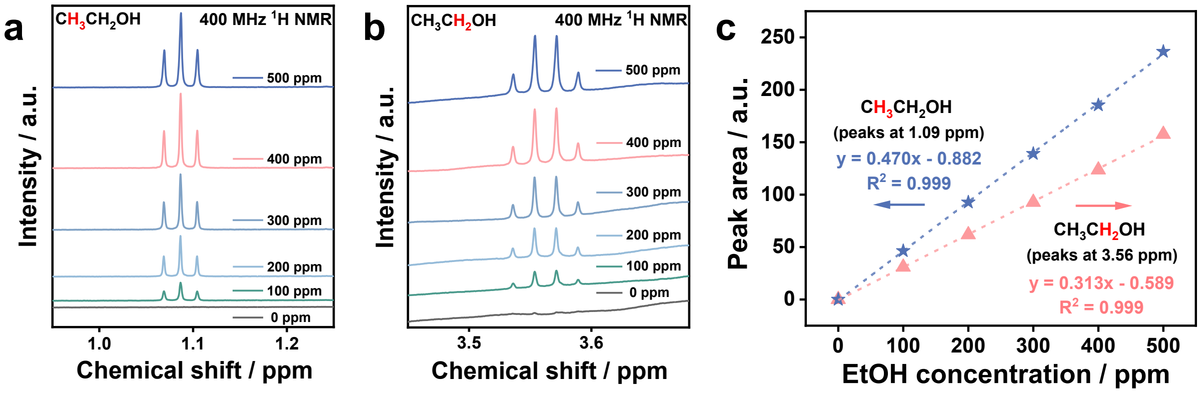

Fig. S16 (a-b) ^{1}H NMR spectra and (c) calibration curves for the quantification of EtOH in the NMR method. NMR spectra of EtOH with concentrations from 0 to 500 ppm in a 400 MHz ^{1}H NMR.

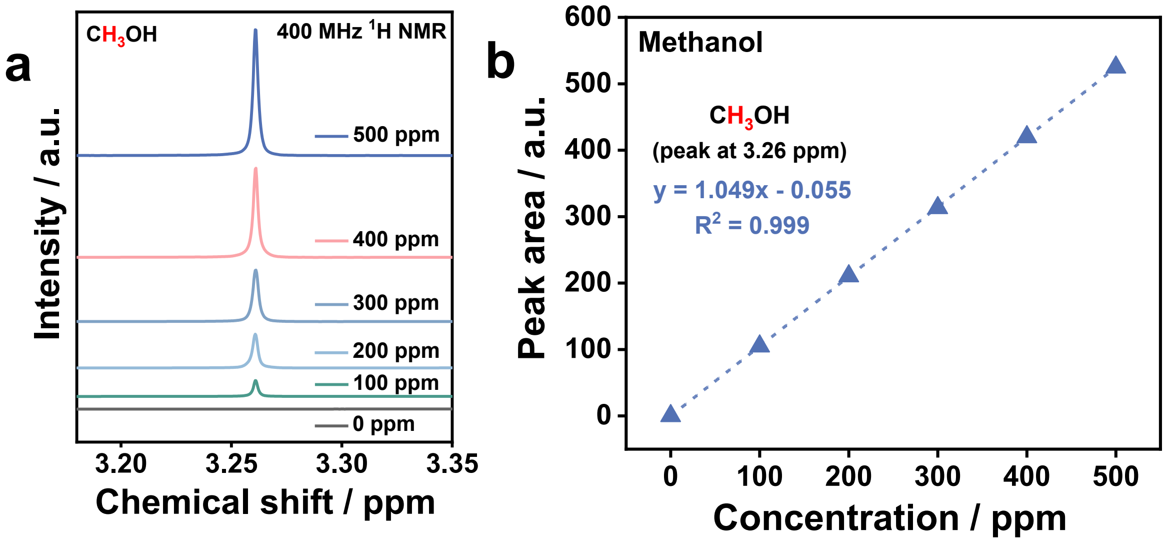

Fig. S17 Quantitative ^{1}H NMR analysis of methanol. (a) ^{1}H NMR spectra of methanol standard solutions at concentrations ranging from 0 to 500 ppm. (b) Corresponding calibration curve for methanol quantification.

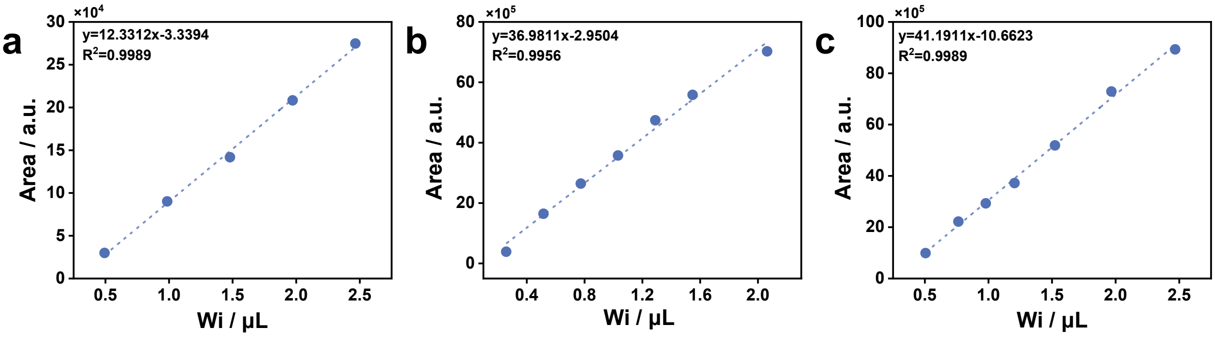

Fig. S18 Calibration curves for (a) H_2, (b) CH_4, and (c) C_2H_4 in the GC method.

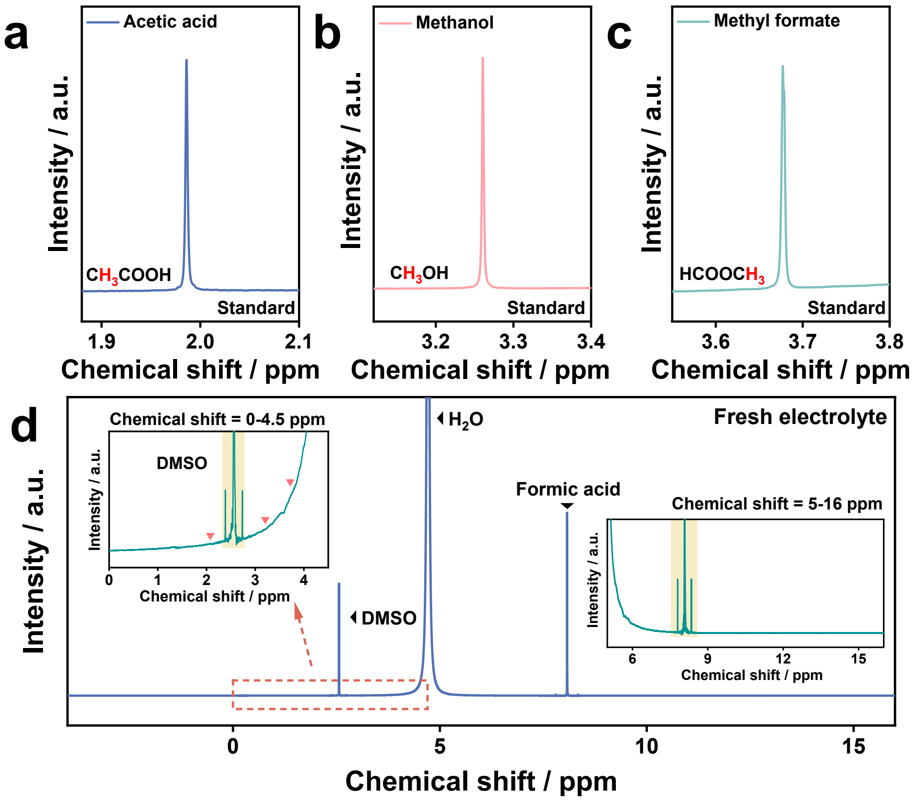

Fig. S19 ^{1}H NMR spectra of standard solutions of possible impurities (a) acetic acid, (b) methanol, and (c) methyl formate in commercial FA. (d) ^{1}H NMR spectrum of fresh electrolyte. The insets display expanded views of the chemical shift regions at 0-4.5 and 5-16 ppm. The red triangles in the 0-4.5 ppm region mark the locations of possible impurities, and no peaks of possible impurities are detected.

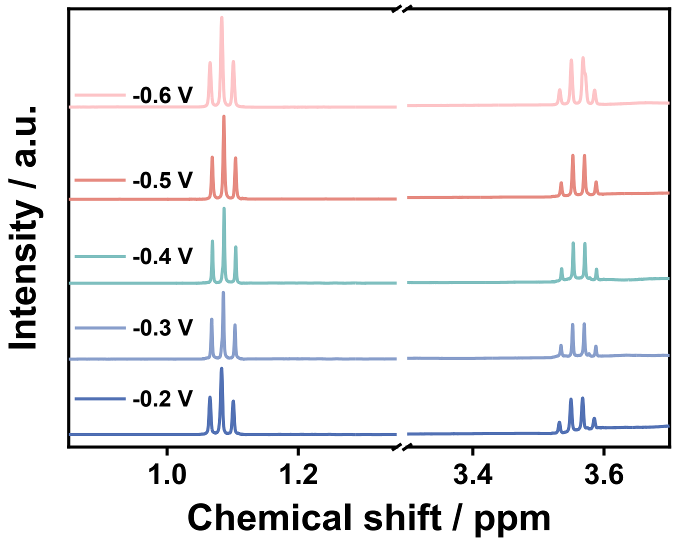

Fig. S20 ^{1}H NMR spectra acquired at various potentials on PdCu/CNT.

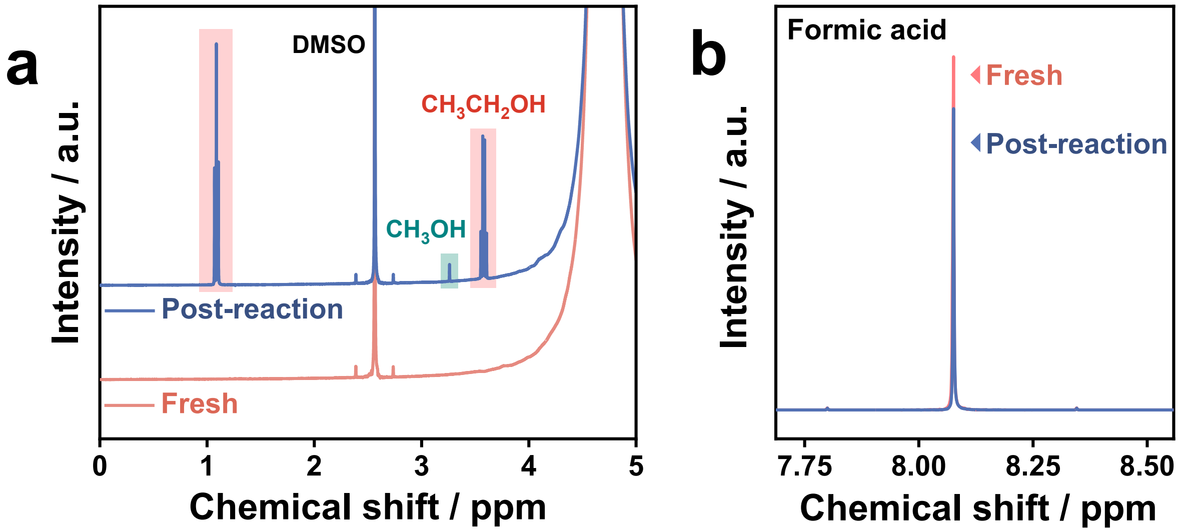

Fig. S21 (a-b) Comparison of ^{1}H NMR spectra of the electrolyte before and after the reaction.

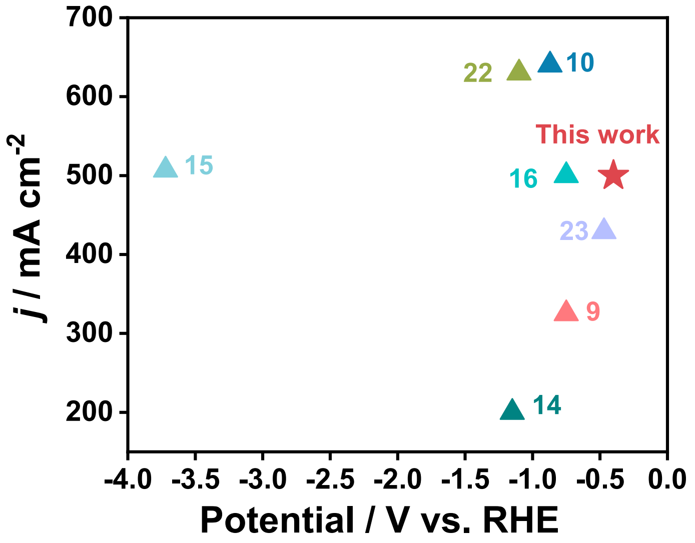

Fig. S22 Comparison of current densities (≥ 200 mA cm^{-2}, labeled in Fig. 1g) and potentials on PdCu/CNT with previous studies of EtOH generation from C_1 reactants. (See Table S2 for details).

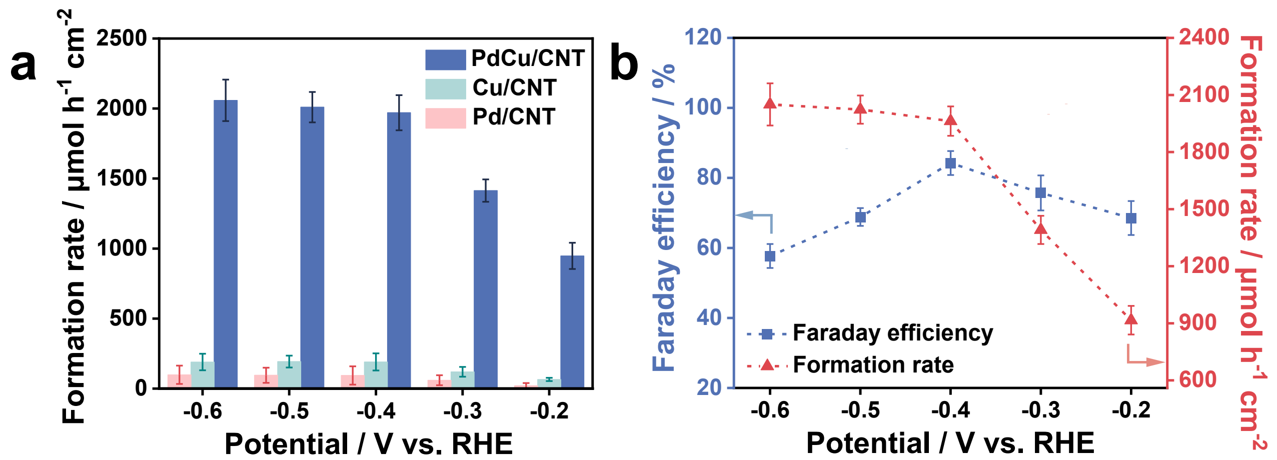

Fig. S23 (a) Formation rates of EtOH over PdCu/CNT, Cu/CNT, and Pd/CNT at different applied potentials. (b) FEs and formation rates of EtOH at different applied potentials for PdCu/CNT produced on a large scale.

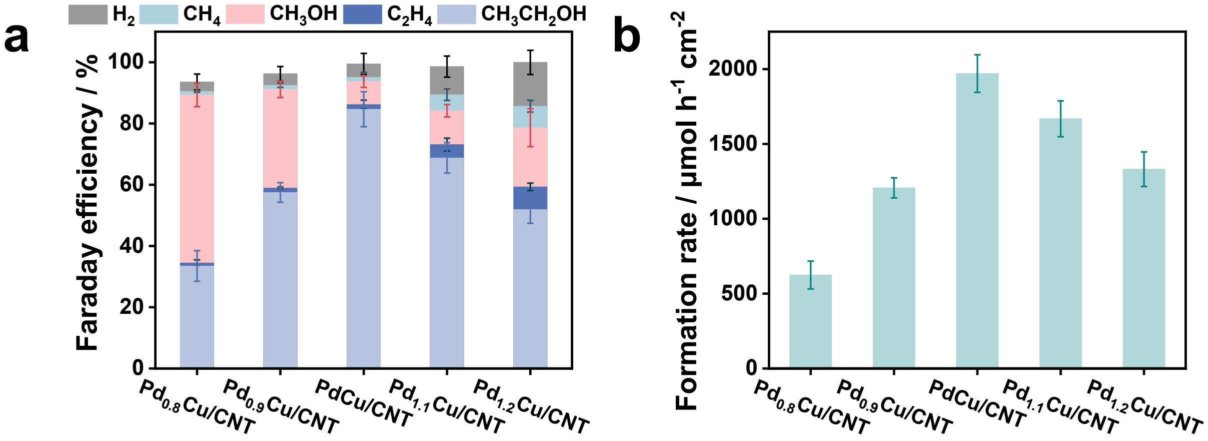

Fig. S24 (a)The product distribution and the corresponding FEs over various electrodes with different Pd/Cu ratios at a potential of -0.4 V vs. RHE with 2-h electrolysis. (b) EtOH formation rates on Pd_xCu/CNT at -0.4 V vs. RHE.

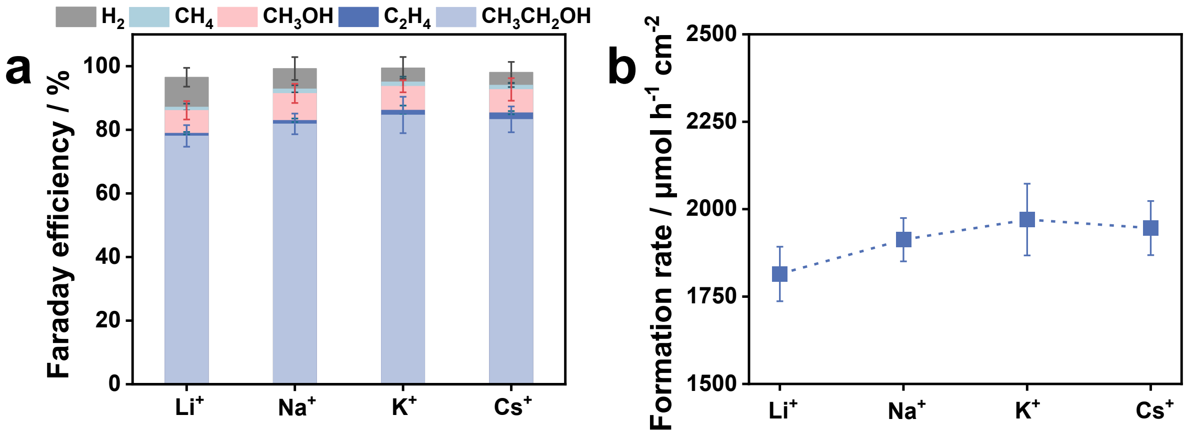

Fig. S25 Effect of alkali metal cations in MOH (M = Li^+, Na^+, K^+, and Cs^+) electrolytes on FRR at -0.4 V vs. RHE (1.0 M FA was added to all solutions). (a) FEs and product distribution in different alkali metal cation electrolytes. (b) Corresponding formation rates of EtOH on various electrolytes.

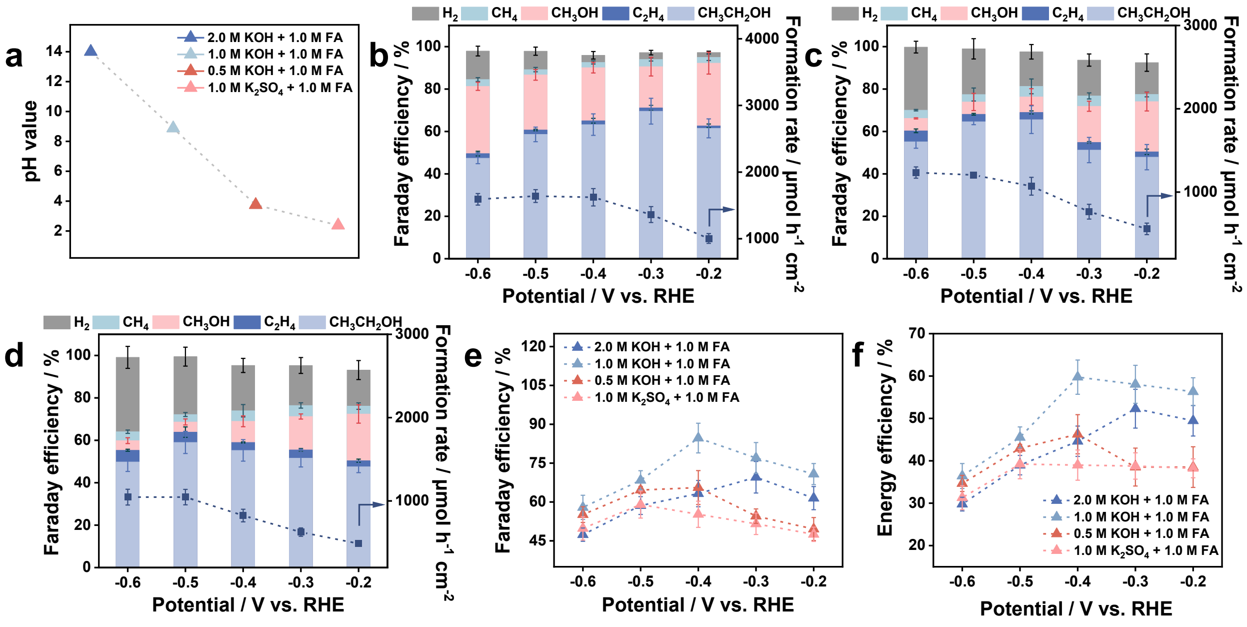

Fig. S26 (a) The pH values for different electrolytes. FEs and formation rates for PdCu/CNT in (b) 2.0 M KOH + 1.0 M FA, (c) 0.5 M KOH + 1.0 M FA, and (d) 1.0 M K_2SO_4 + 1.0 M FA electrolytes. Comparison of (e) FEs and (f) EEs of EtOH in KOH electrolytes with different concentrations on PdCu/CNT.

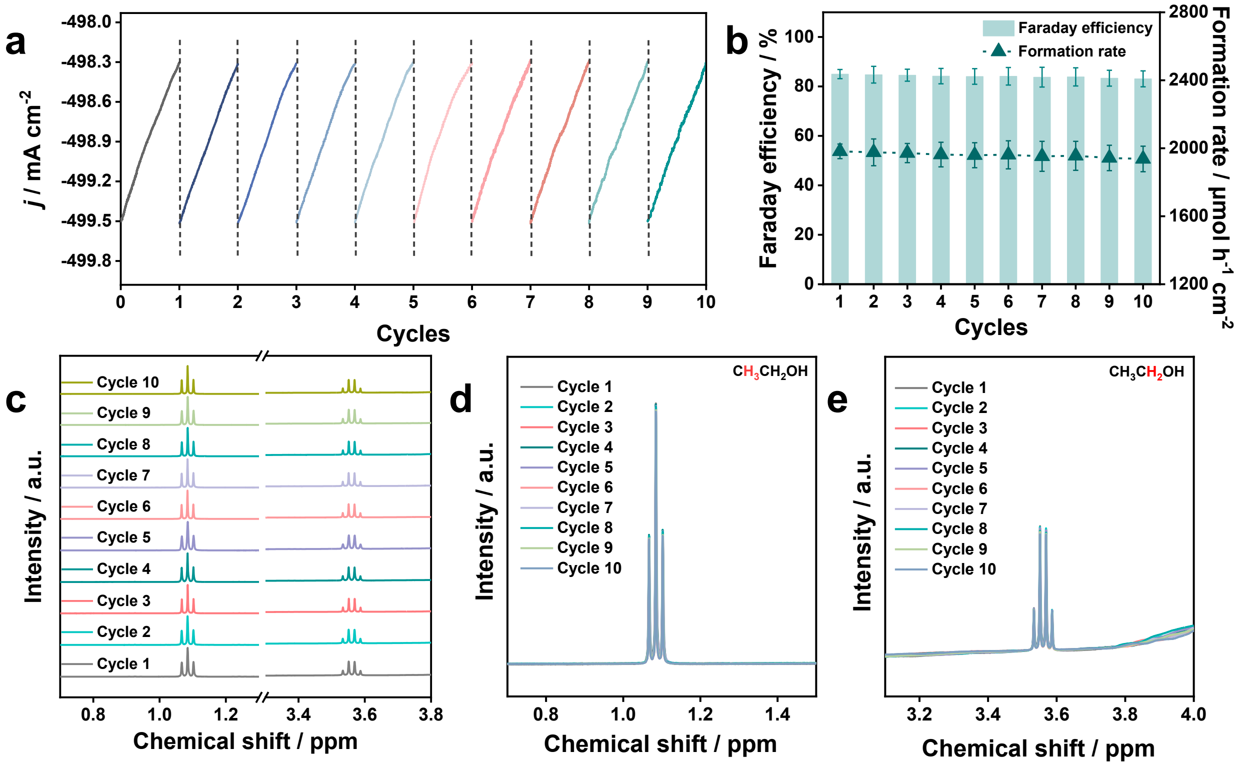

Fig. S27 (a) The stability test of FRR for PdCu/CNT under 10 cycles (2 hours per cycle). (b) FEs and formation rates of electroreductive coupling of FA to EtOH in a single electrolysis cycle. (c-e) ^{1}H NMR spectra of EtOH in each cycle.

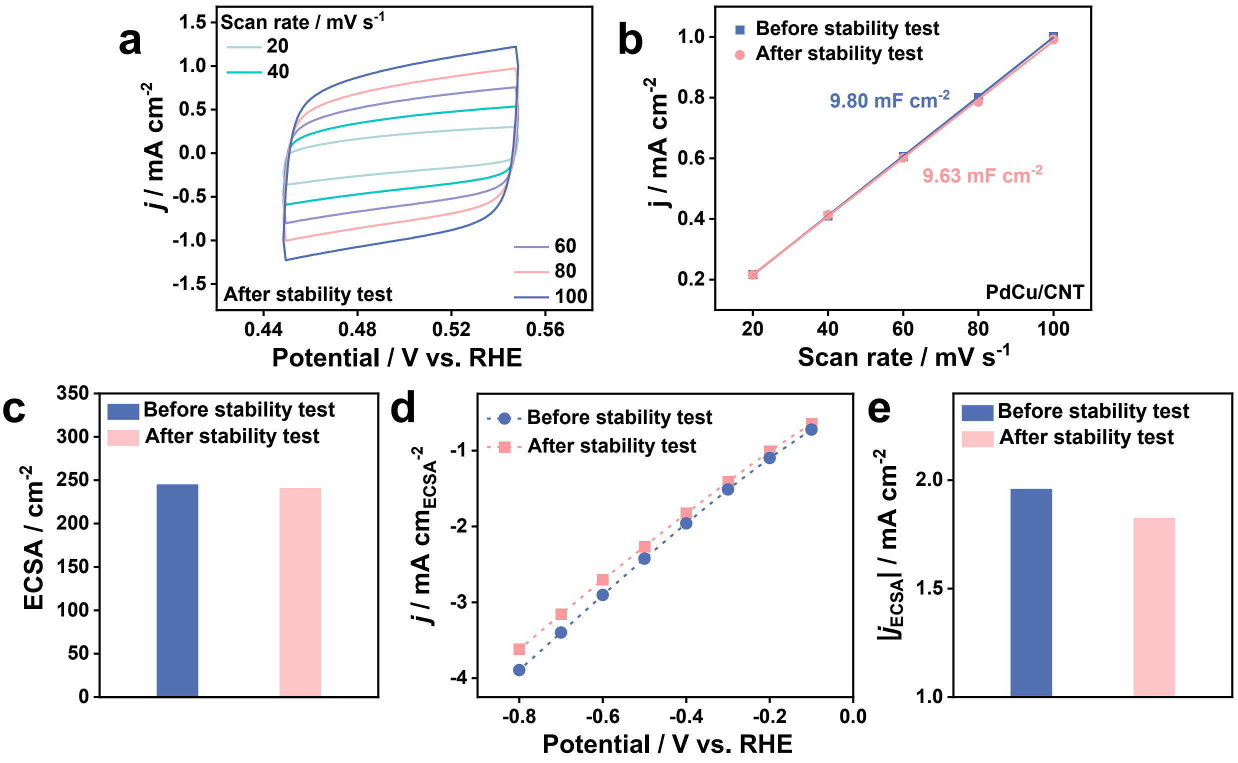

Fig. S28 (a) CV curves at different scan rates after stability test. (b) C_{dl} and (c) ECSA of PdCu/CNT prior to and post per stability test. (d) Specific activity of PdCu/CNT normalized by ECSA before and after stability test. (e) Absolute current density normalized by ECSA, $|j_{ECSA}|$ at -0.4 V vs. RHE.

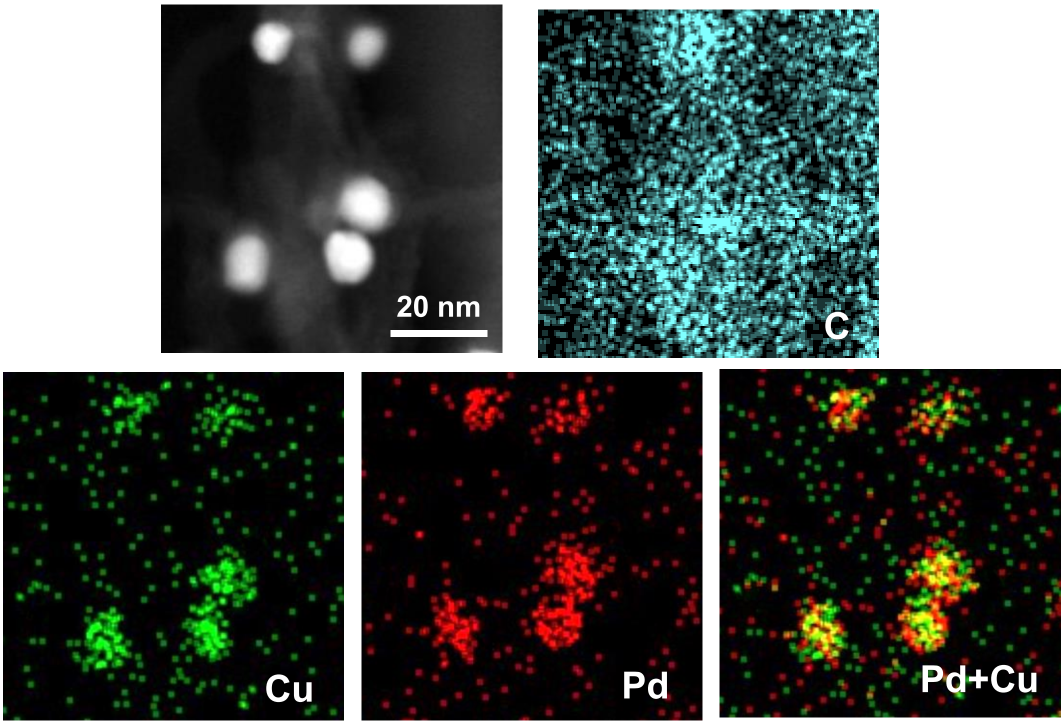

Fig. S29 HAADF-TEM image and the corresponding EDS elemental mapping of PdCu/CNT catalyst after stability test.

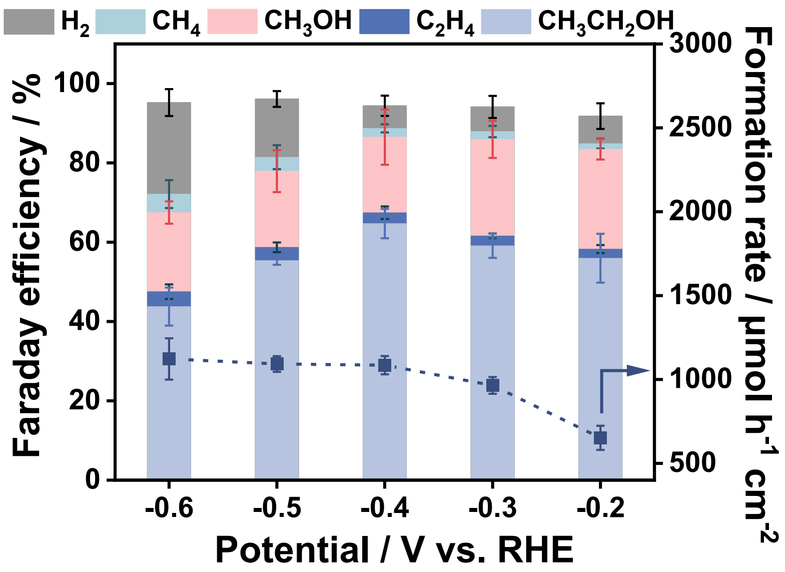

Fig. S30 Comparison of FEs and formation rates for PdCu alloy nanoparticles at various applied potentials.

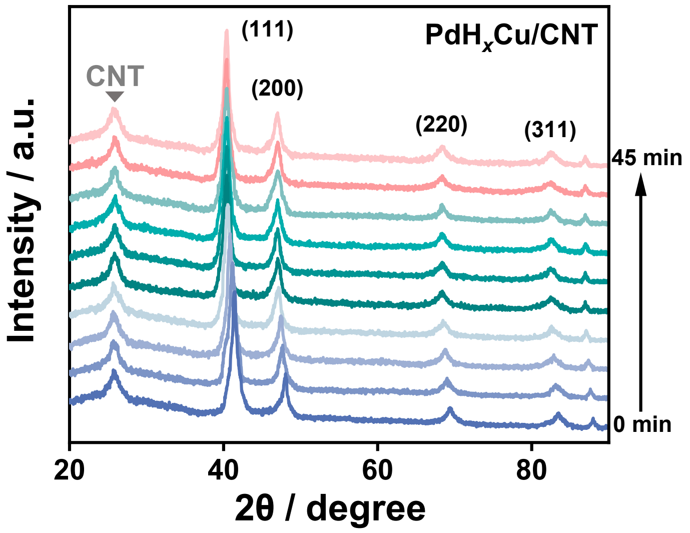

Fig. S31 *In situ* electrochemical XRD patterns of PdH$_x$Cu/CNT.

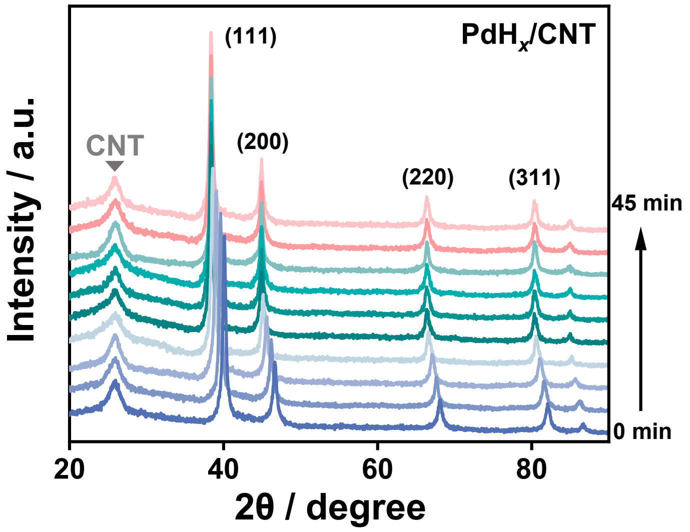

Fig. S32 *In situ* electrochemical XRD patterns of PdH$_x$/CNT.

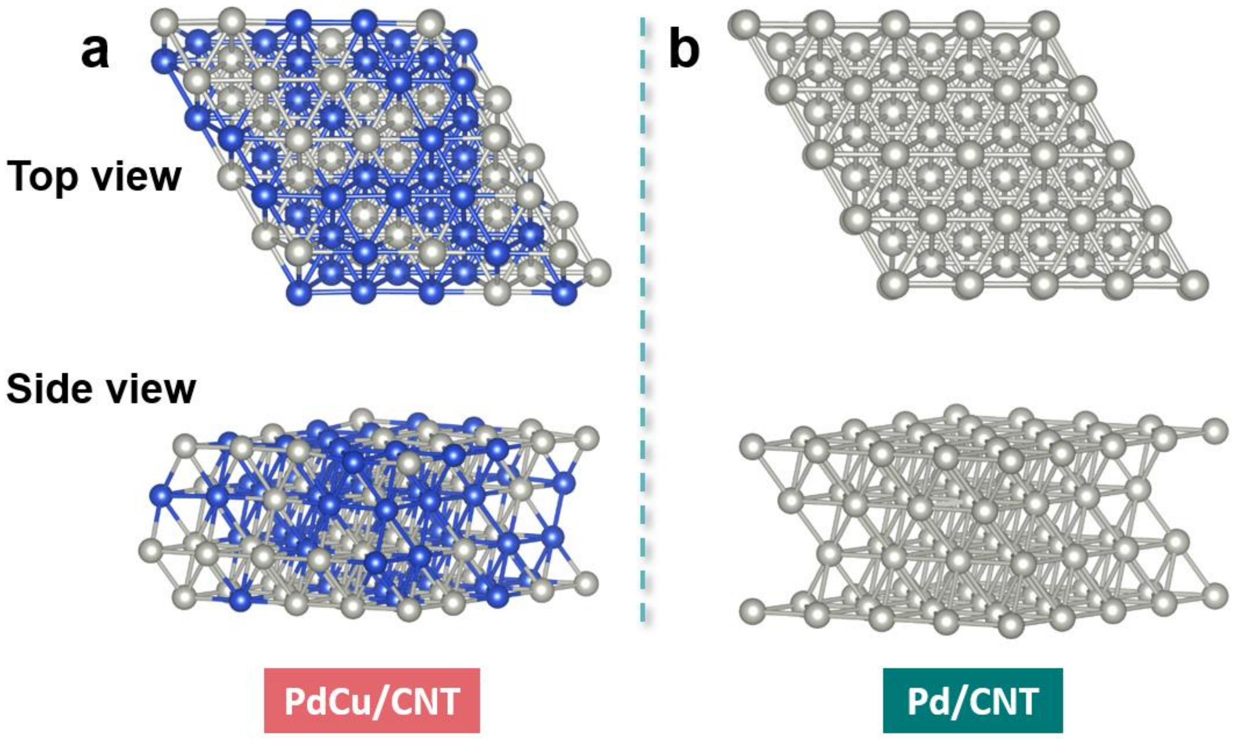

Fig. S33 Top and side views of (a) PdCu (111) and (b) Pd (111) models. Color code: blue: Cu; gray: Pd.

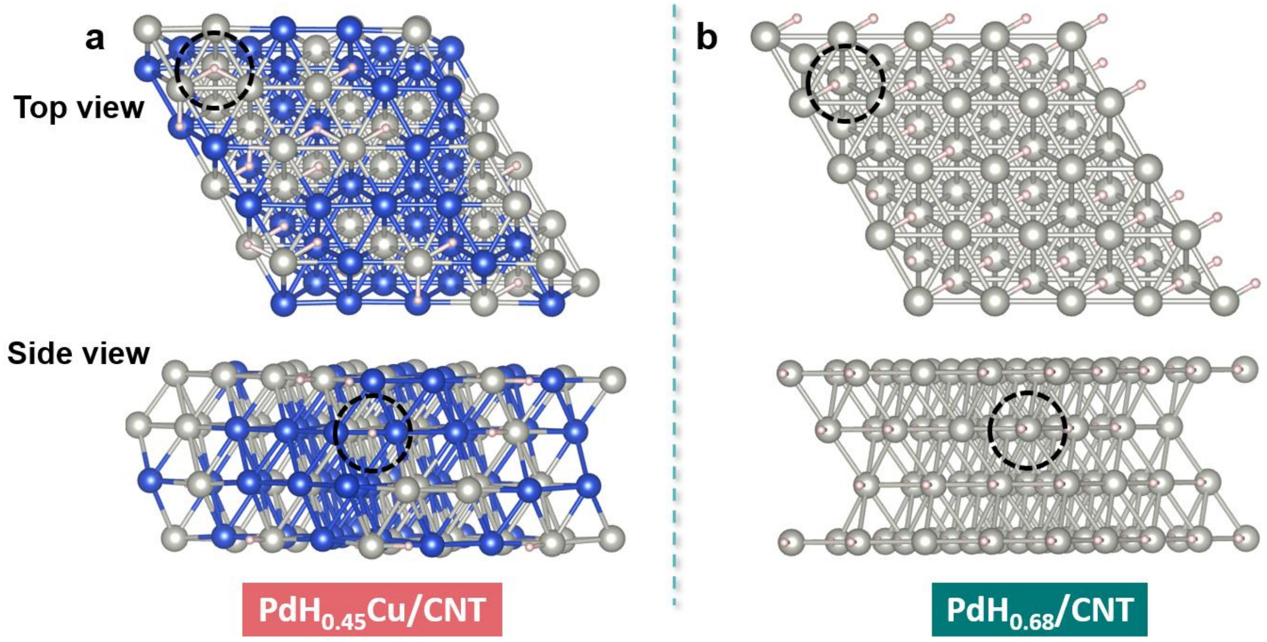

Fig. S34 Top and side views of (a) $PdH_{0.45}Cu$ (111) and (b) $PdH_{0.68}$ (111) models.

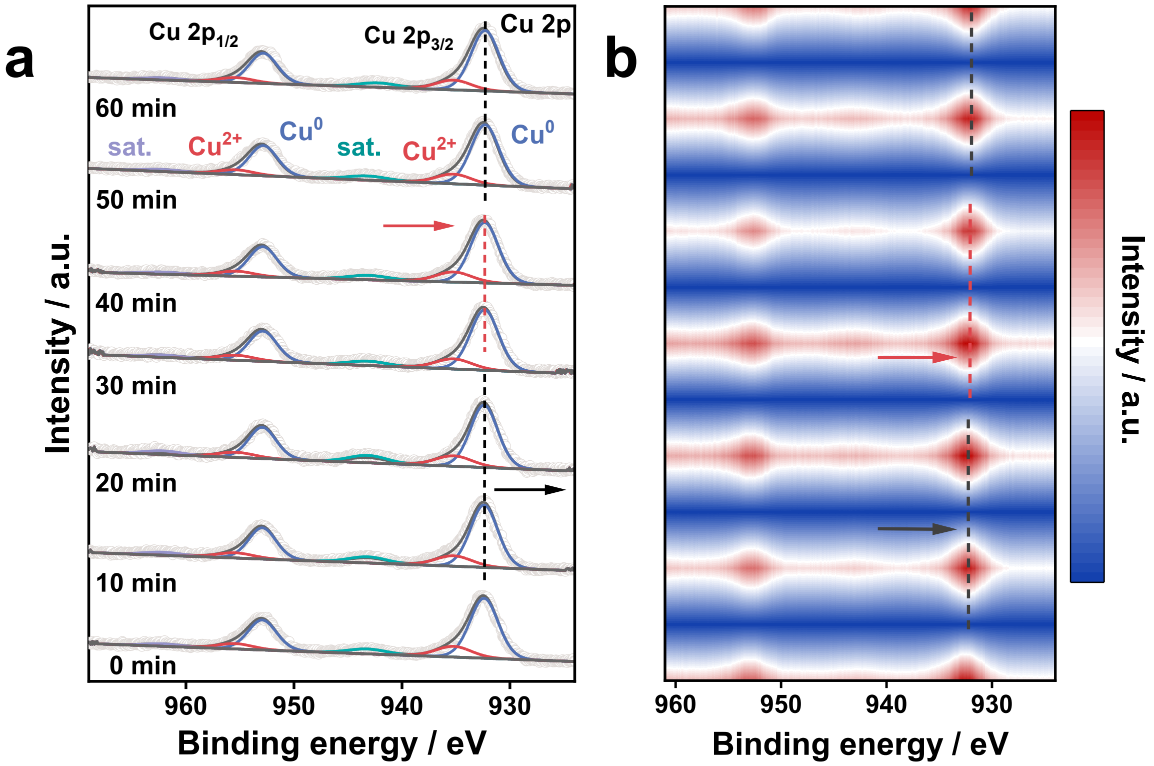

Fig. S35 (a) Time-dependent quasi-*in situ* XPS spectra and (b) the corresponding equi-contour plots for Cu 2p of PdH_xCu/CNT.

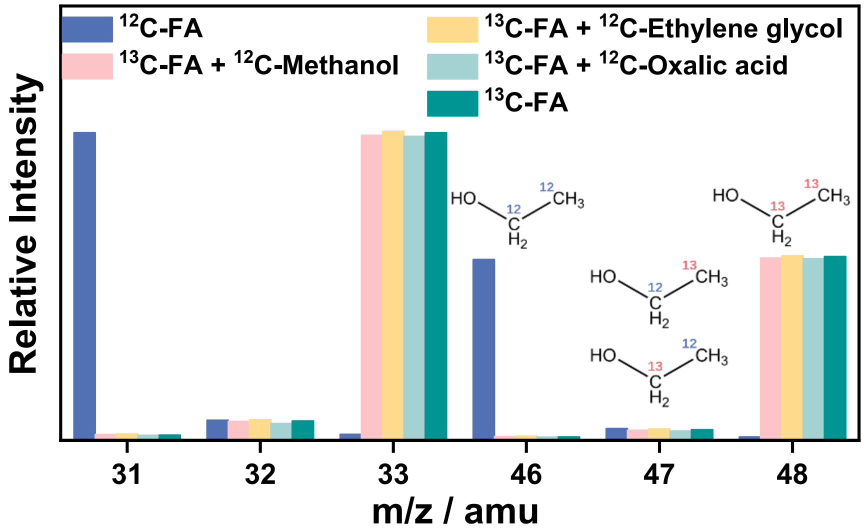

Fig. S36 Mass spectra of EtOH produced from FA self-coupling reaction using ^{12}C-FA, ^{13}C-FA + ^{12}C-methanol, ^{13}C-FA + ^{12}C-ethylene glycol, ^{13}C-FA + ^{12}C-oxalic acid, and ^{13}C-FA as feedstocks.

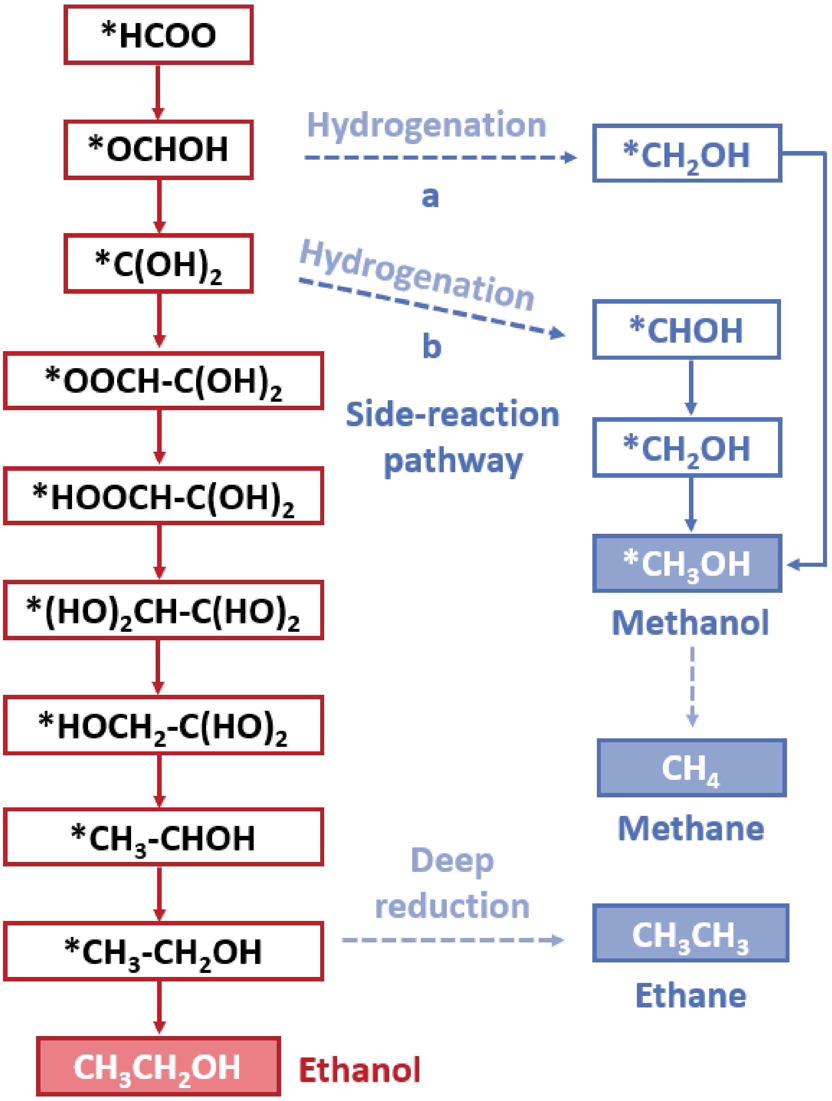

Fig. S37 Pathway of FA self-coupling reaction for the generation of EtOH (left red frame), and the main side-reaction pathways for the production of methanol, methane, and ethane (right blue frame).

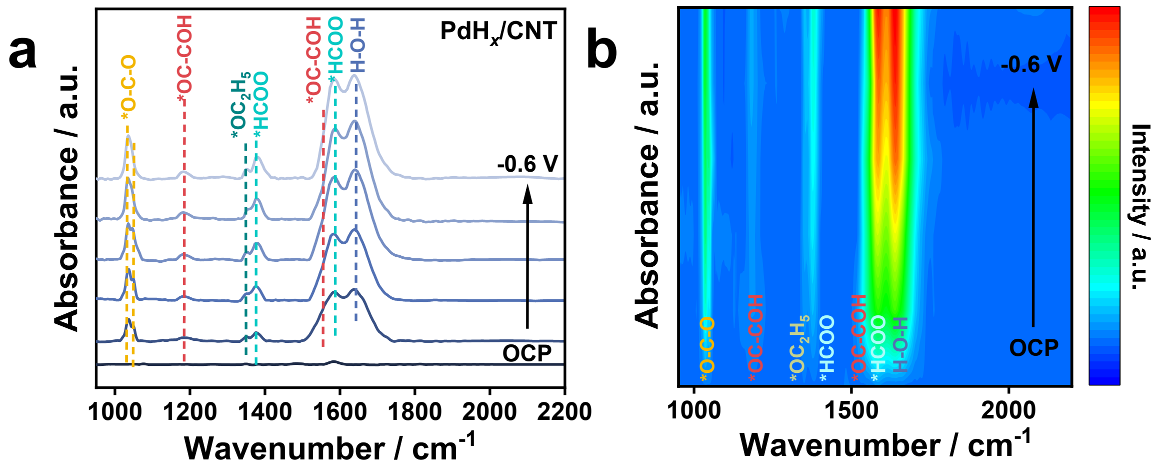

Fig. S38 (a) *In situ* FTIR spectra and (b) the corresponding two-dimensional spectra of PdH$_x$/CNT.

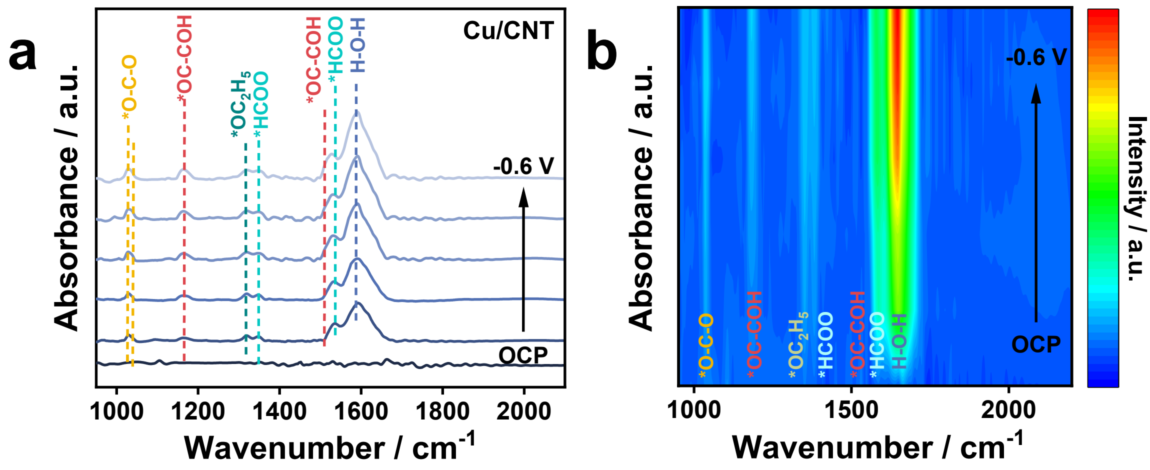

Fig. S39 (a) *In situ* FTIR spectra and (b) the corresponding equi-contour plots for Cu/CNT.

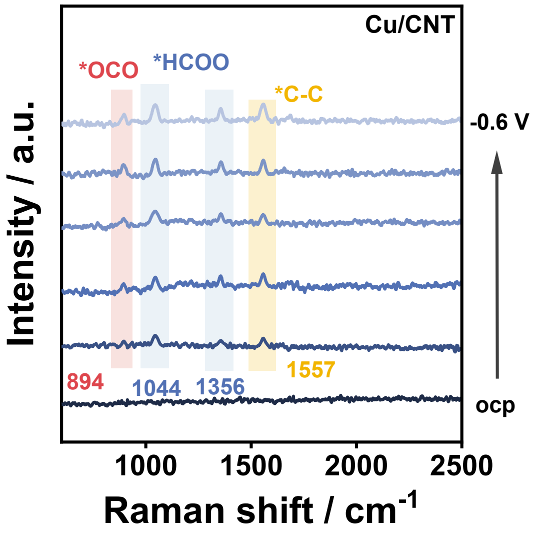

Fig. S40 *In situ* Raman spectra of Cu/CNT.

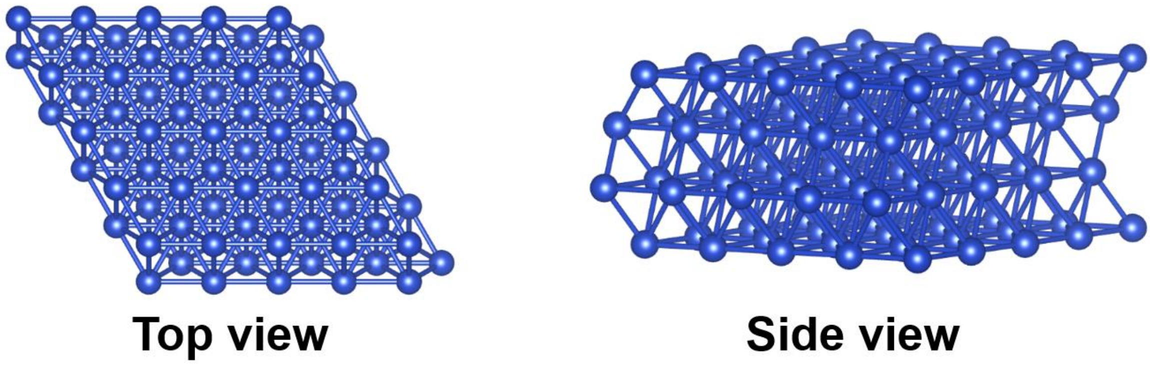

Fig. S41 Top and side views of Cu (111) model. Blue spheres represent Cu.

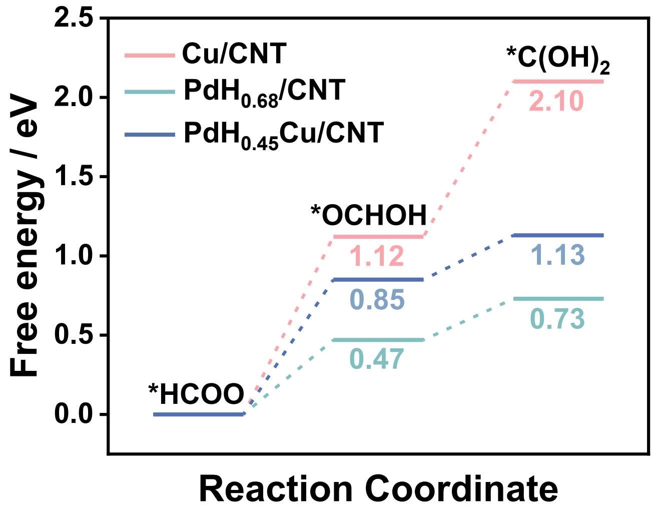

Fig. S42 Energy profiles for *HCOO, *OCHOH, and $*C(OH)_2$ adsorption on Cu/CNT, $PdH_{0.68}$/CNT, and $PdH_{0.45}Cu$/CNT.

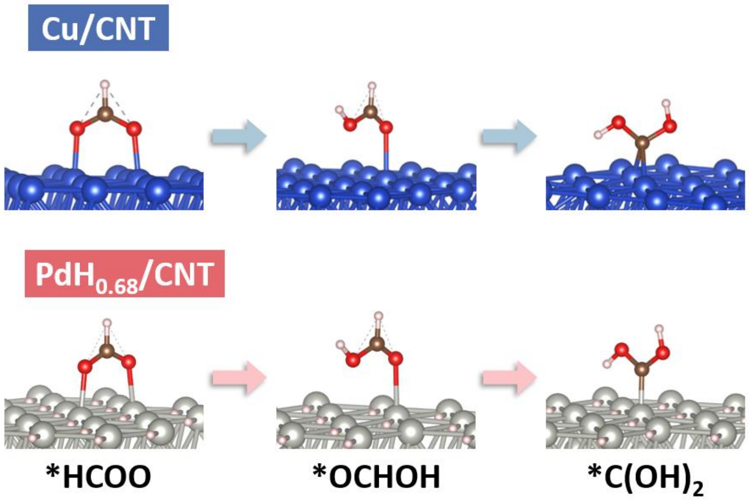

Fig. S43 Structures related to the adsorption of *HCOO, *HCOO, and $*C(OH)_2$ on Cu/CNT and $PdH_{0.68}$/CNT. Color code: brown: C; white: H; red: O; blue: Cu; gray: Pd (the color code is applied to all computational models in subsequent figures).

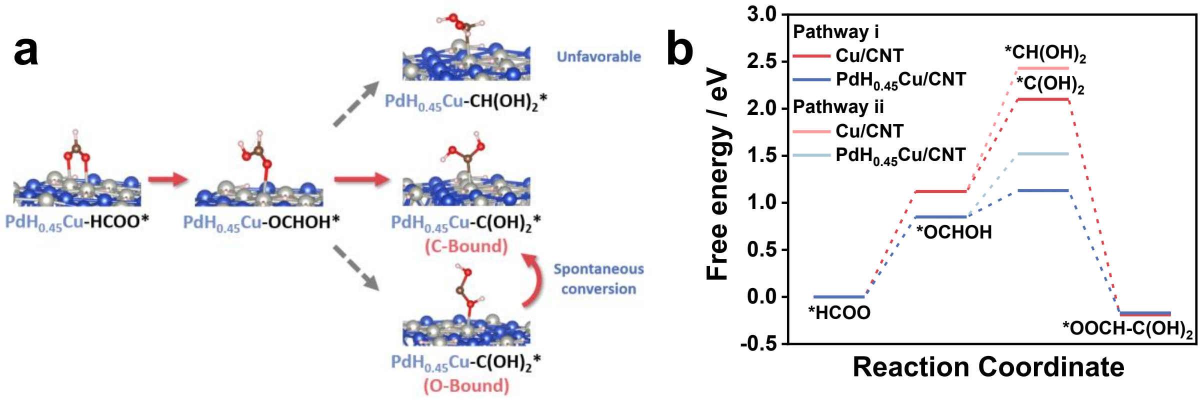

Fig. S44 (a) Optimized structure of intermediates involved in the *HCOO conversion process on $PdH_{0.45}Cu/CNT$. (b) Free energy diagram of the C-C coupling pathway (i) and $*CH(OH)_2$ formation pathways (ii) on $PdH_{0.45}Cu/CNT$ and Cu/CNT.

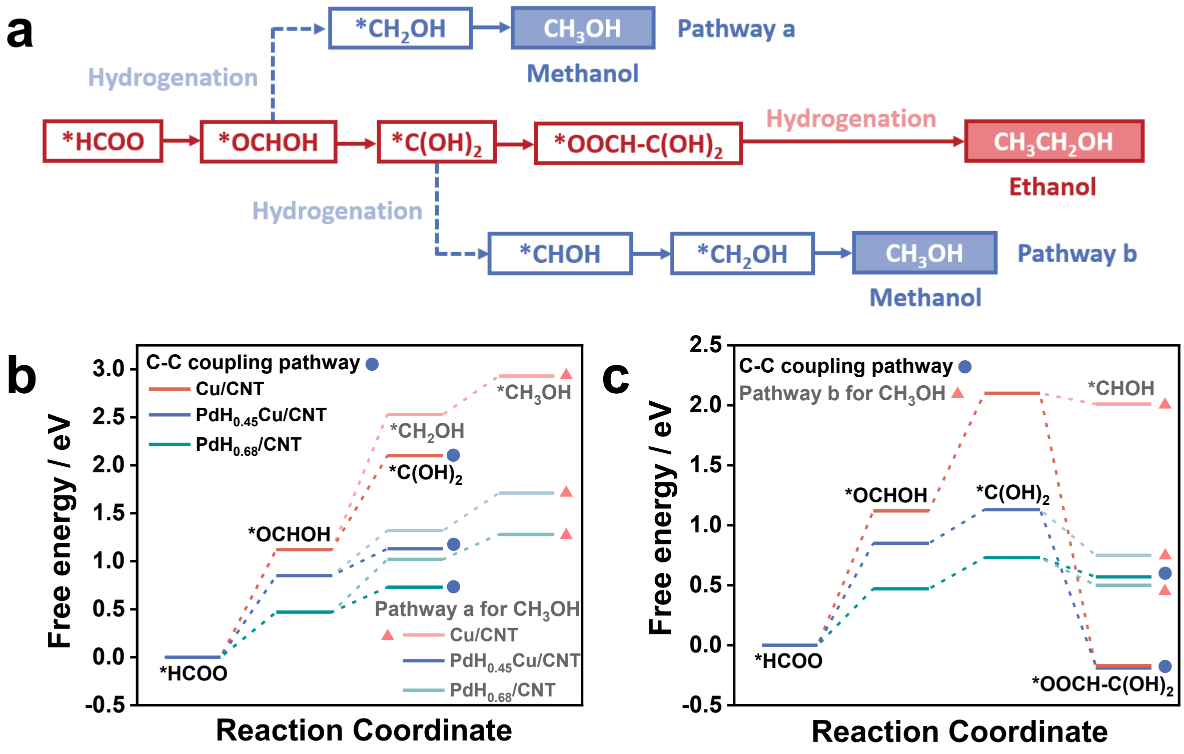

Fig. S45 (a) Schematic representation of the proposed reaction mechanism (based on Fig. S37). The red path represents the C-C coupling pathway, while the blue paths represent the two possible pathways, a and b, for methanol production. (b-c) Free energy diagram for methanol generation and C-C coupling reactions on Cu/CNT, $PdH_{0.68}/CNT$, and $PdH_{0.45}Cu/CNT$.

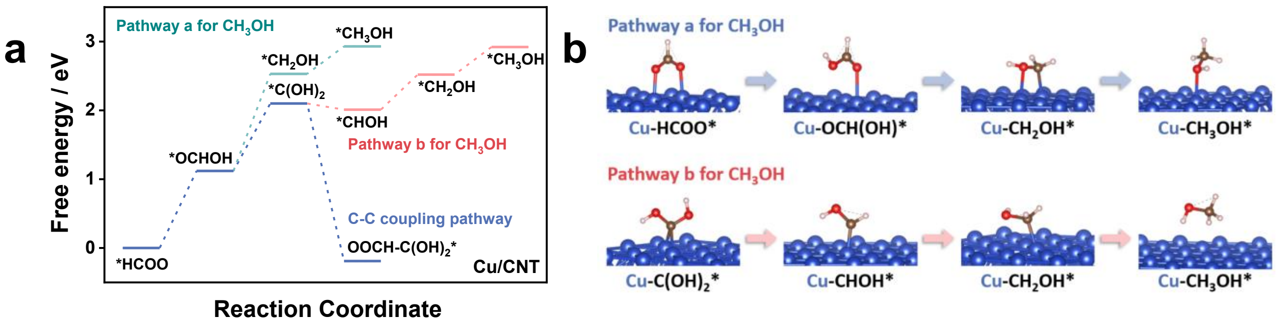

Fig. S46 (a) Energy profiles of the C-C coupling pathway and by-product CH_3OH formation pathways on Cu/CNT, and (b) the corresponding structures associated with the adsorption of all reaction intermediates in CH_3OH formation pathways.

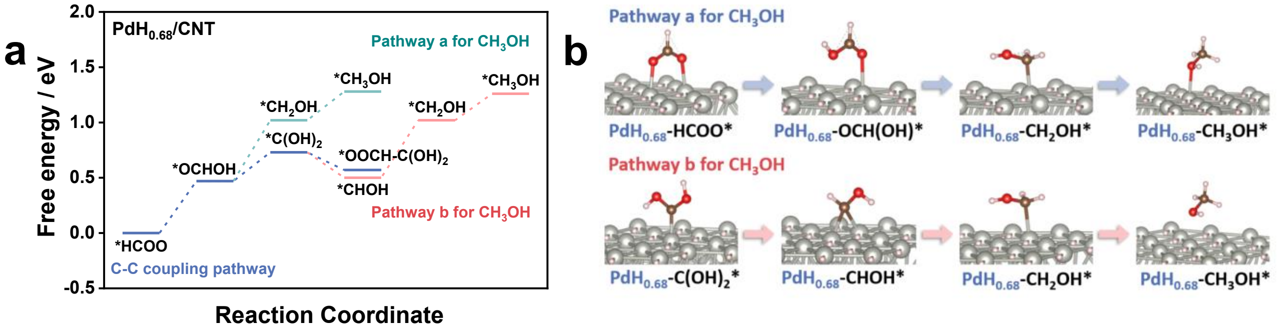

Fig. S47 (a) Free energy diagram of the C-C coupling pathway and by-product CH_3OH formation pathways (a and b) on $PdH_{0.68}$/CNT. (b) Optimized structures of all reaction intermediates involved in CH_3OH formation pathways on $PdH_{0.68}$/CNT.

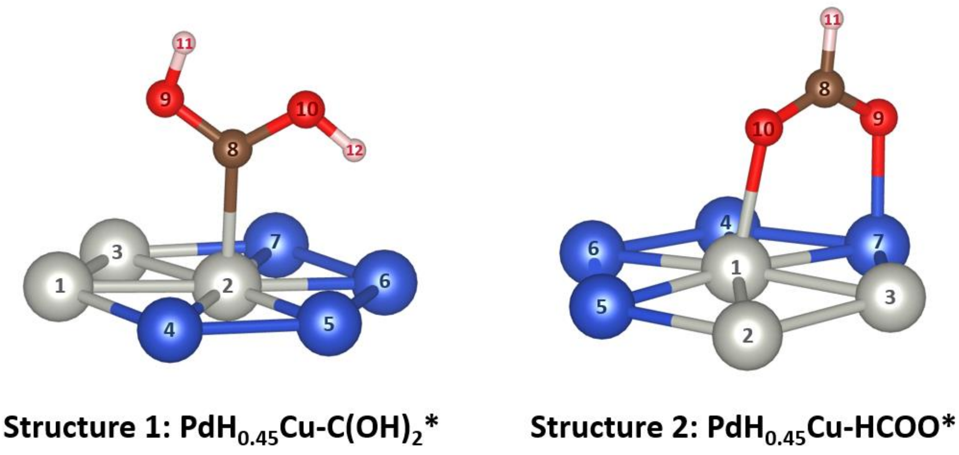

Fig. S48 Serial numbers corresponding to each atom in structures 1 and 2. Fukui function values for each atom in the two structures are shown in Table S7-8.

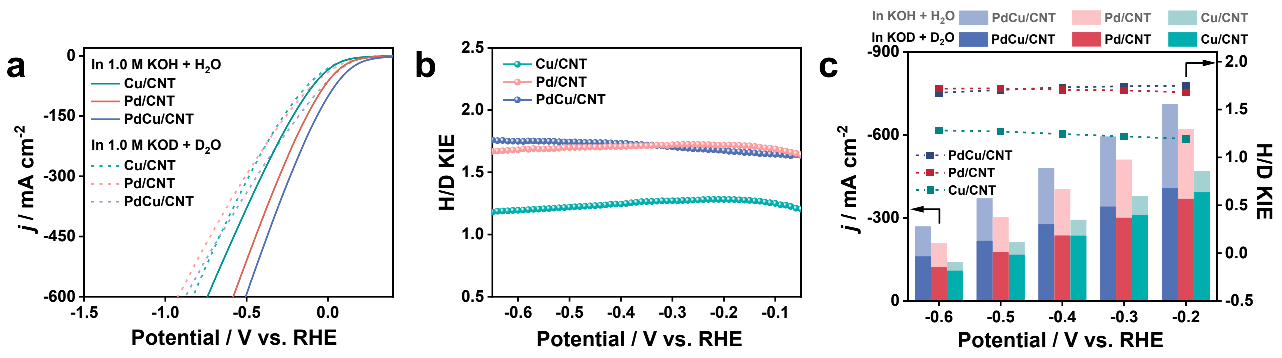

Fig. S49 (a) Polarization curves of Cu/CNT, Pd/CNT, and PdCu/CNT in KOH + H_2O and KOD + D_2O. (b) KIE plots of Cu/CNT, Pd/CNT, and PdCu/CNT. (c) Comparison of current density variations and KIE values for three catalysts at different potentials.

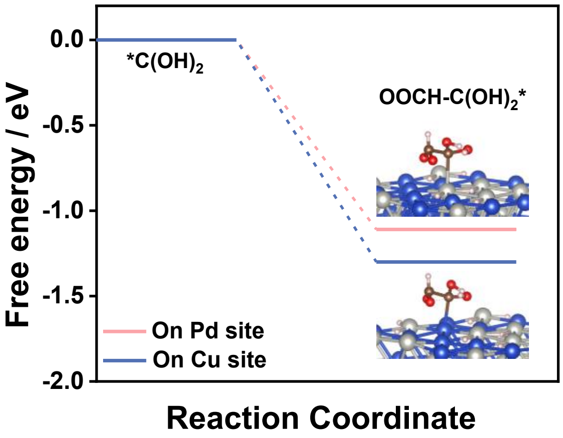

Fig. S50 Free energy diagrams for the C-C coupling step at Pd and Cu sites on $PdH_{0.45}Cu$/CNT.

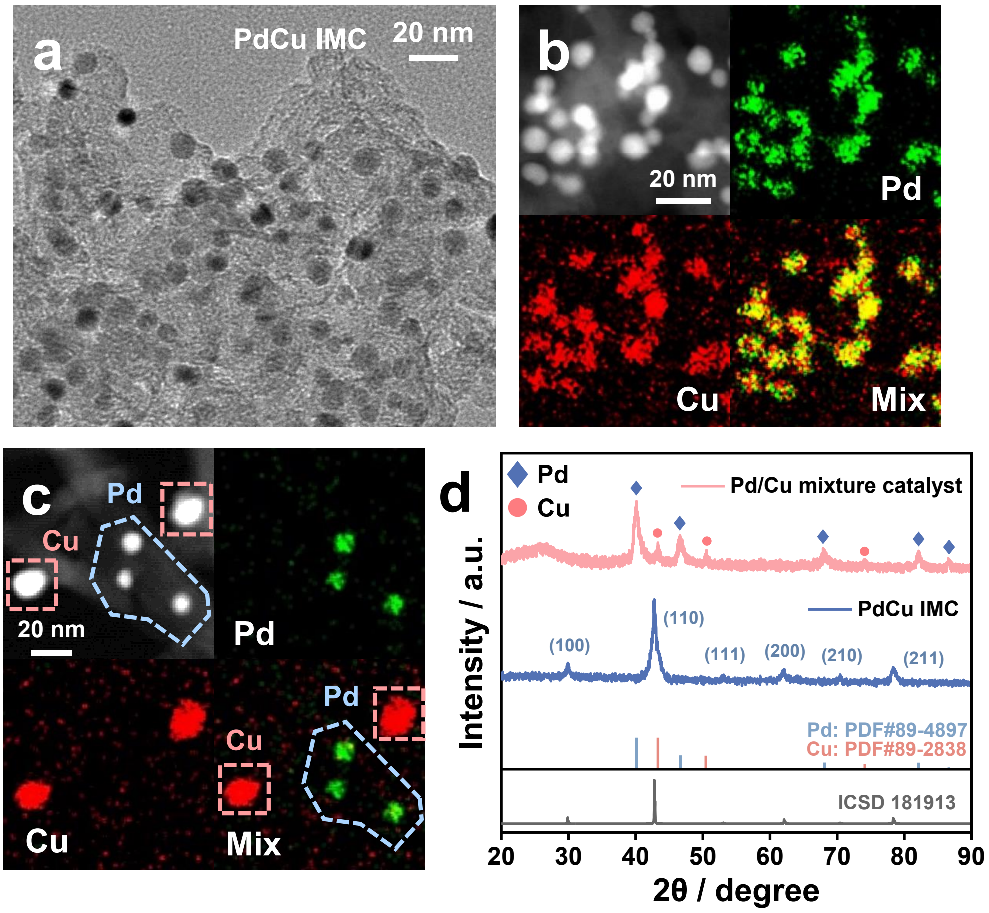

Fig. S51 (a) TEM image of PdCu IMC. HAADF-STEM and the corresponding EDS-mapping images of (b) PdCu IMC, and (c) co-anchored Pd/Cu mixture catalysts. (d) XRD patterns for various PdCu catalysts.

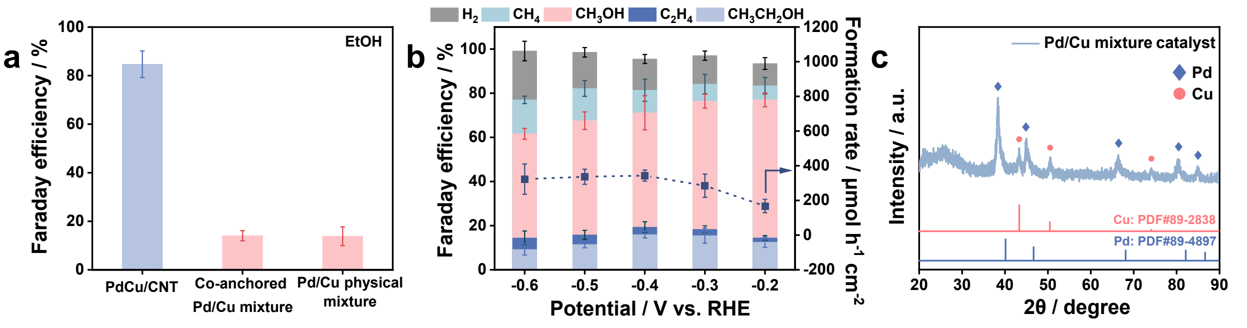

Fig. S52 (a) Comparison of FEs and product distributions for PdCu/CNT, co-anchored Pd/Cu mixture, and physical mixture of Pd/Cu catalysts at -0.4 V vs. RHE. (b) Comparison of FEs, product distributions, and formation rates for PdCu IMC at various applied potentials. (c) XRD pattern of the physical mixture of Pd/Cu catalyst after reaction.

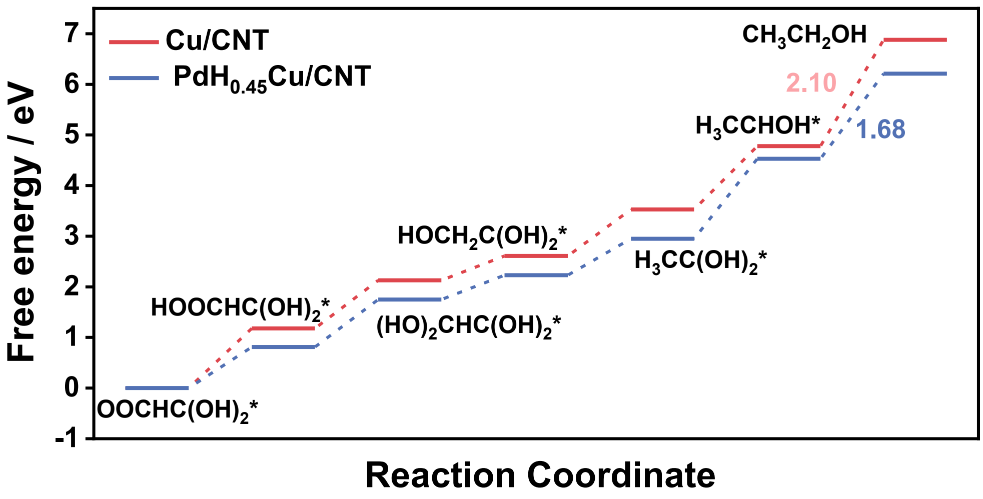

Fig. S53 Free energy diagram for hydrogenation steps on $PdH_{0.45}Cu$/CNT and Cu/CNT.

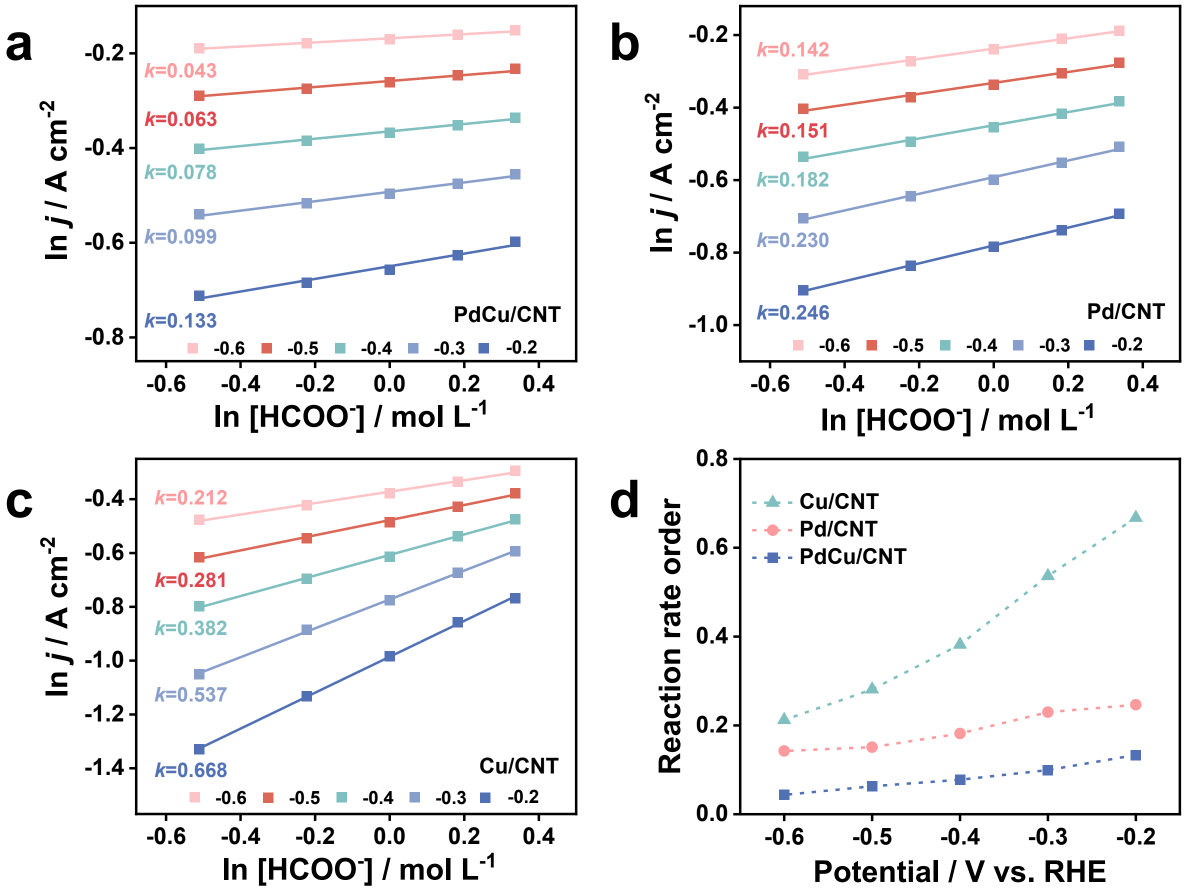

Fig. S54 Linear fit of ln *j* against ln [HCOO$^-$] of (a) PdCu/CNT, (b) Pd/CNT, and (c) Cu/CNT for FRR at different potentials, the slope of the fitted lines (*k*) represented the rate order of formate reduction. (d) Comparison of the reaction rate order of formate reduction of PdCu/CNT, Pd/CNT, and Cu/CNT.

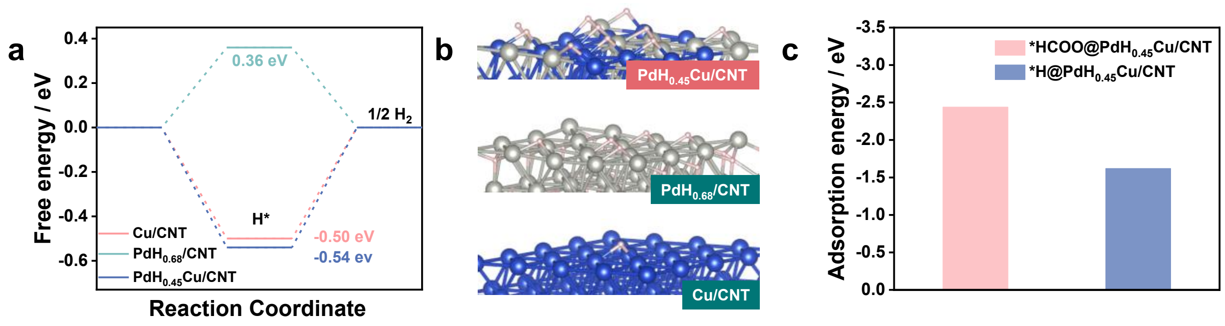

Fig. S55 (a) Free energy diagram of HER on $PdH_{0.45}Cu$/CNT, $PdH_{0.68}$/CNT, and Cu/CNT. (b) Schematic illustration of HER on different catalysts. (c) Adsorption energies of $PdH_{0.45}Cu$/CNT for *HCOO and *H.

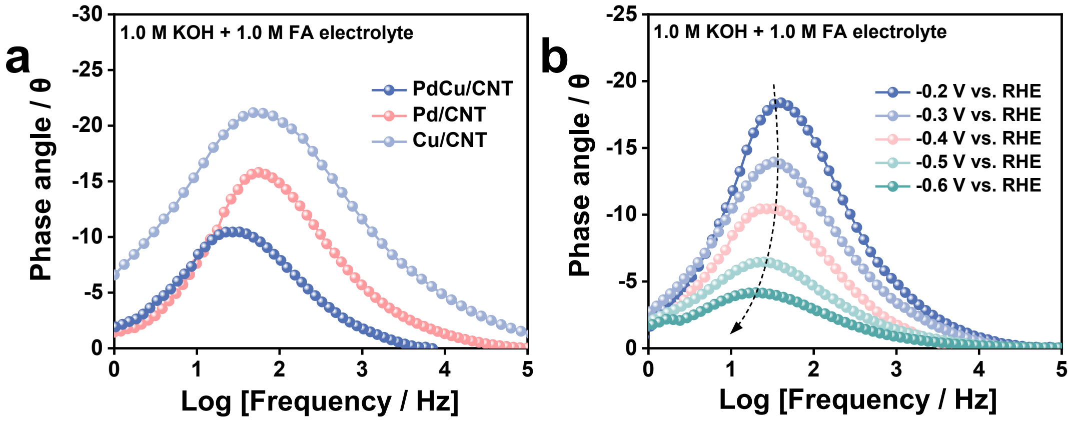

Fig. S56 (a) Bode phase plots PdCu/CNT, Pd/CNT, and Cu/CNT in 1.0 M KOH + 1.0 M FA electrolyte at -0.4 V vs. RHE. (b) Bode phase plots of PdCu/CNT at different applied potentials in 1.0 M KOH + 1.0 M FA electrolyte.

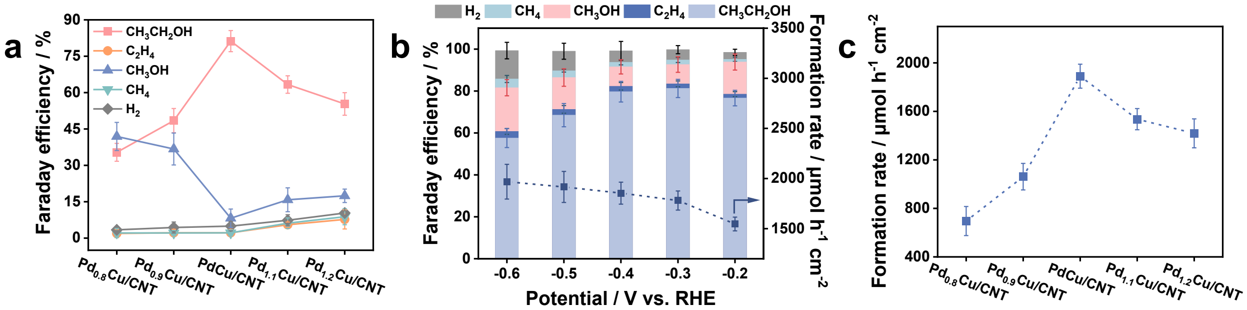

Fig. S57 (a) FEs of all products on Pd_xCu/CNT with different Pd/Cu ratios. (b) FEs and product distributions on PdCu/CNT at various applied potentials. (c) The formation rate of EtOH (from formaldehyde reduction) on PdCu/CNT at different potentials.

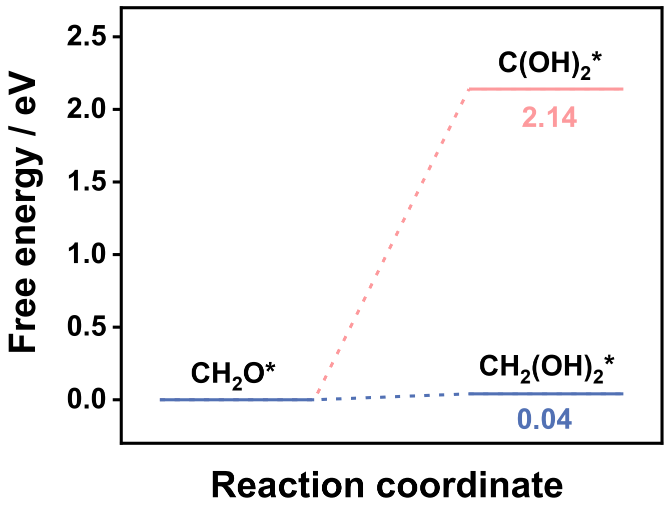

Fig. S58 Free energy diagram of adsorbed $*CH_2O$ conversion.

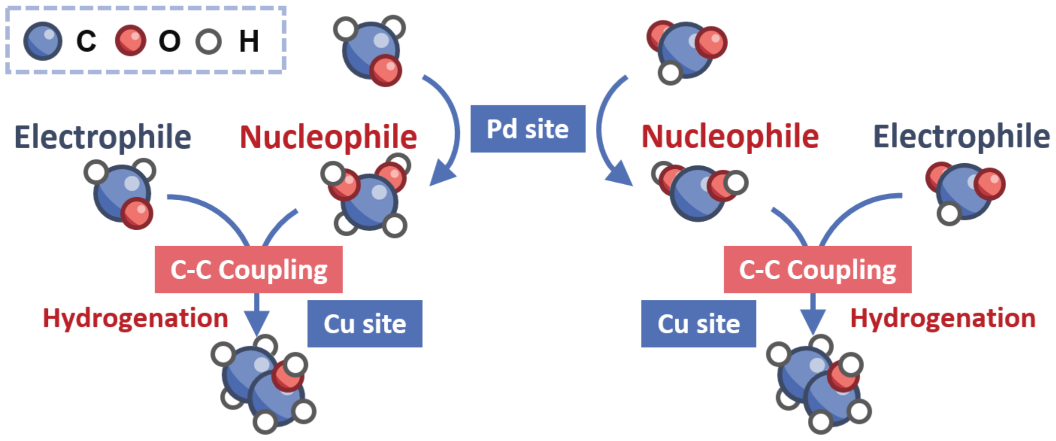

Fig. S59 Schematic illustration of the proposed reaction mechanism for formaldehyde (left) and formate (right) reduction reactions to generate EtOH.

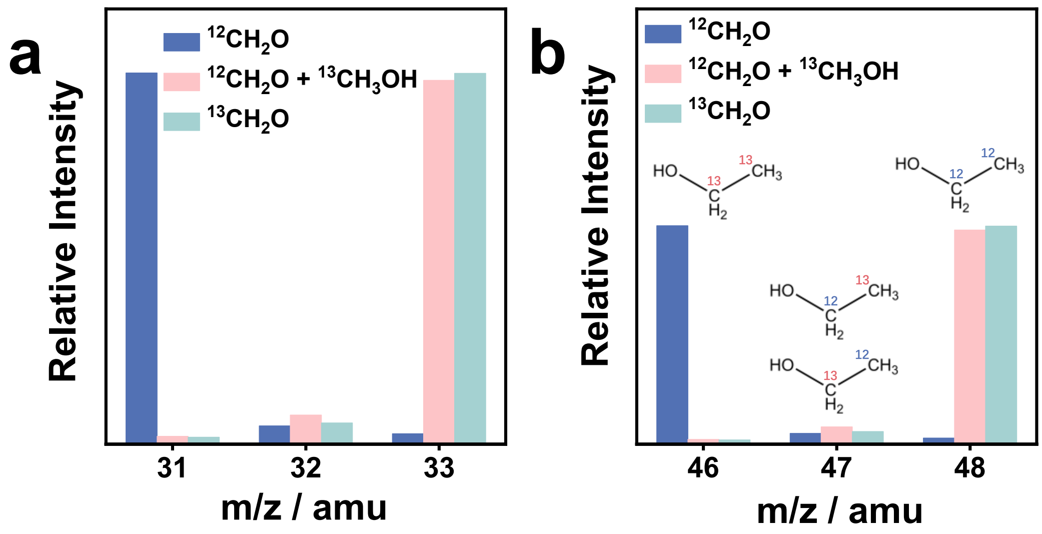

Fig. S60 Mass spectra of EtOH produced from formaldehyde self-coupling reaction using $^{12}CH_2O$, $^{13}CH_2O$ + $^{12}CH_3OH$, and $^{13}CH_2O$ as feedstocks.

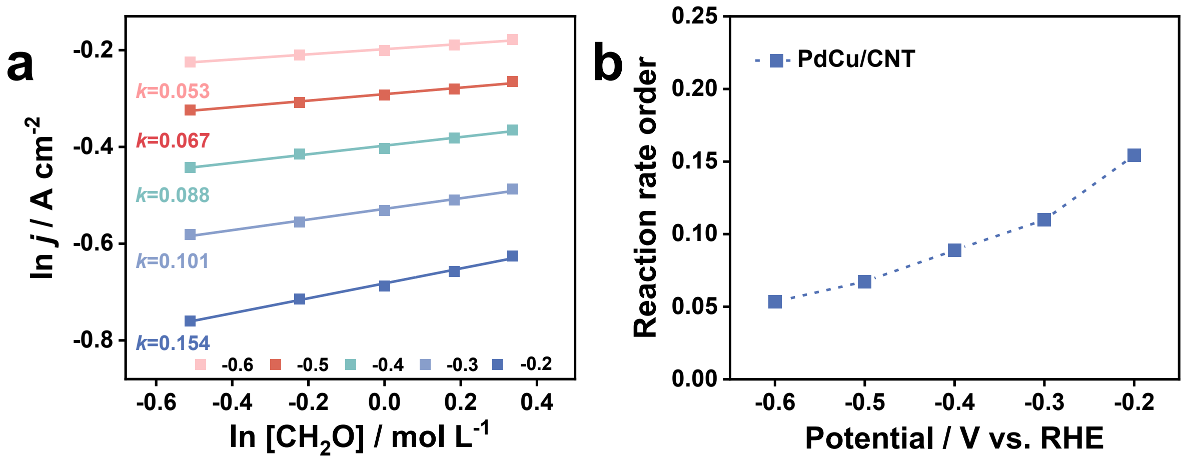

Fig. S61 (a) Linear fitting of ln [*j*] against ln [CH_2O] of PdCu/CNT at different potentials, with the slope (*k*) of the fitted line representing the rate order for the formaldehyde electroreduction. (b) Obtained rate orders for PdCu/CNT.

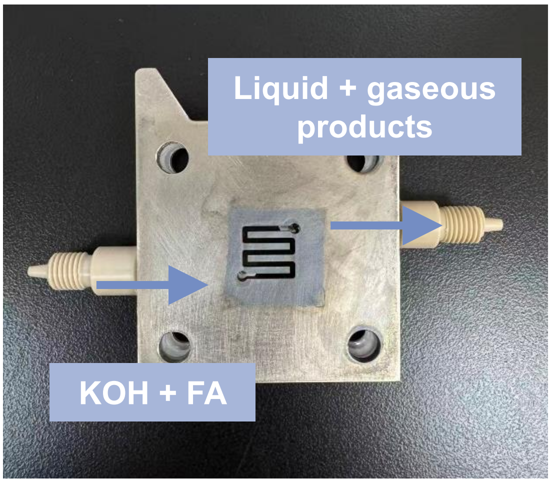

Fig. S62 Schematic illustration of a serpentine-type flow channel for the AEM electrolyzer.

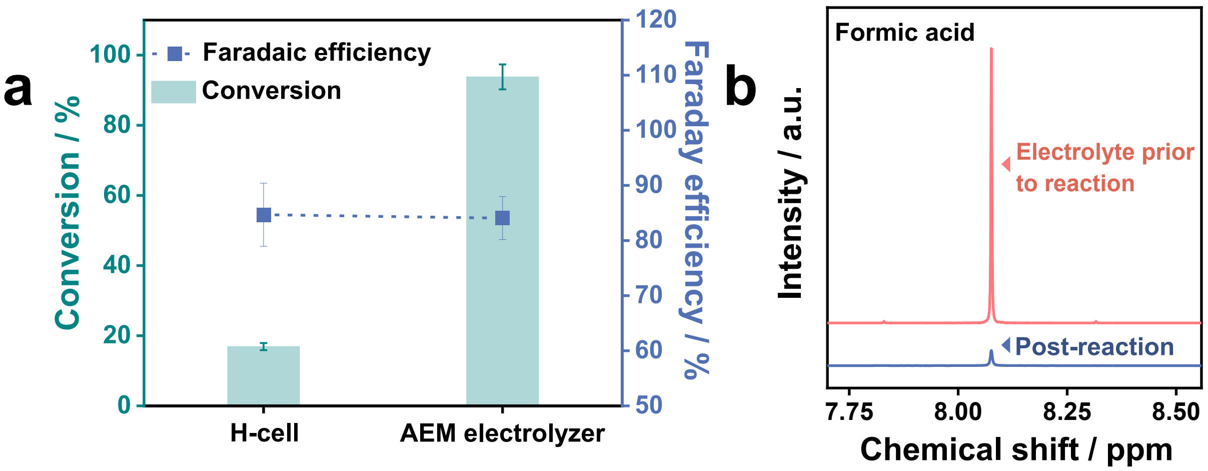

Fig. S63 (a) Comparison of conversion and FE at 500 mA cm^{-2} after 2 h of H-cell experiments and 12 h of reaction in the AEM electrolyzer. (b) ^1H NMR spectra of the electrolyte collected before and after the stability test in the AEM electrolyzer.

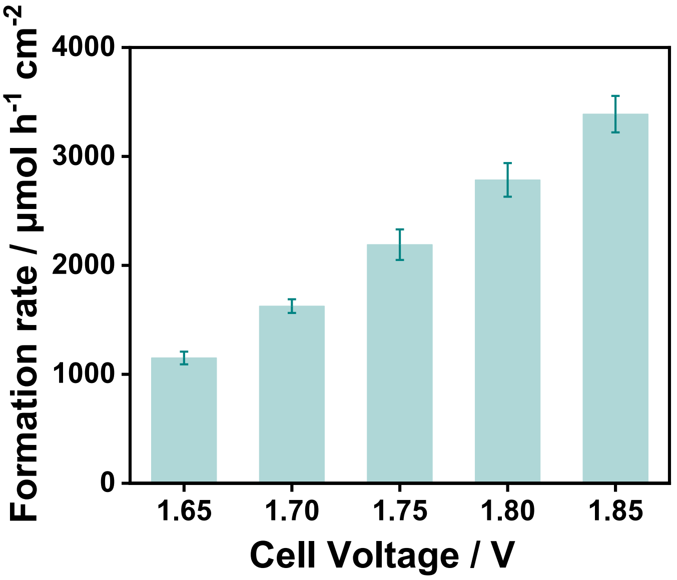

Fig. S64 Formation rate of EtOH at different cell voltages.

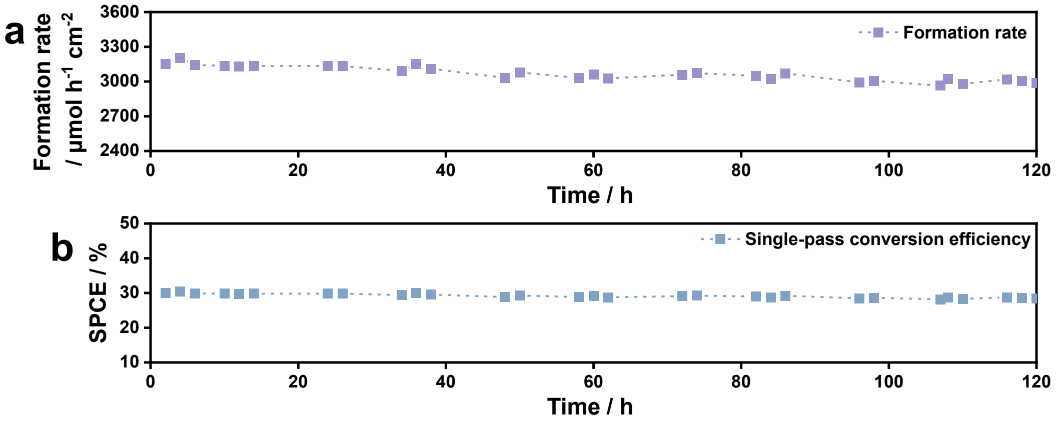

Fig. S65 (a) Formation rate and (b) SPCE during long-term EtOH electrosynthesis at 800 mA cm^{-2}.

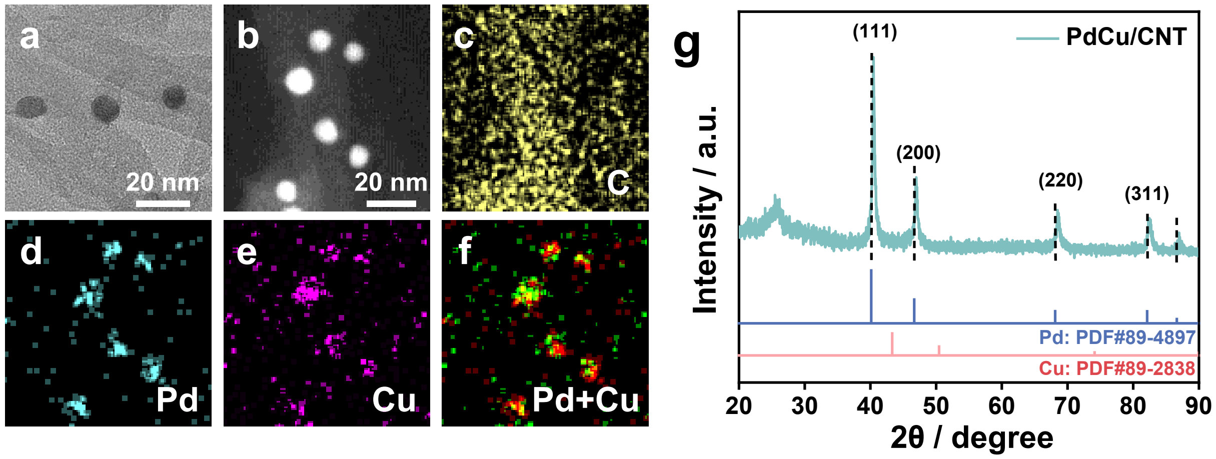

Fig. S66 (a) TEM, (b) HAADF-STEM and (c-f) corresponding EDS mapping images, as well as (g) XRD pattern for PdCu/CNT after stability test.

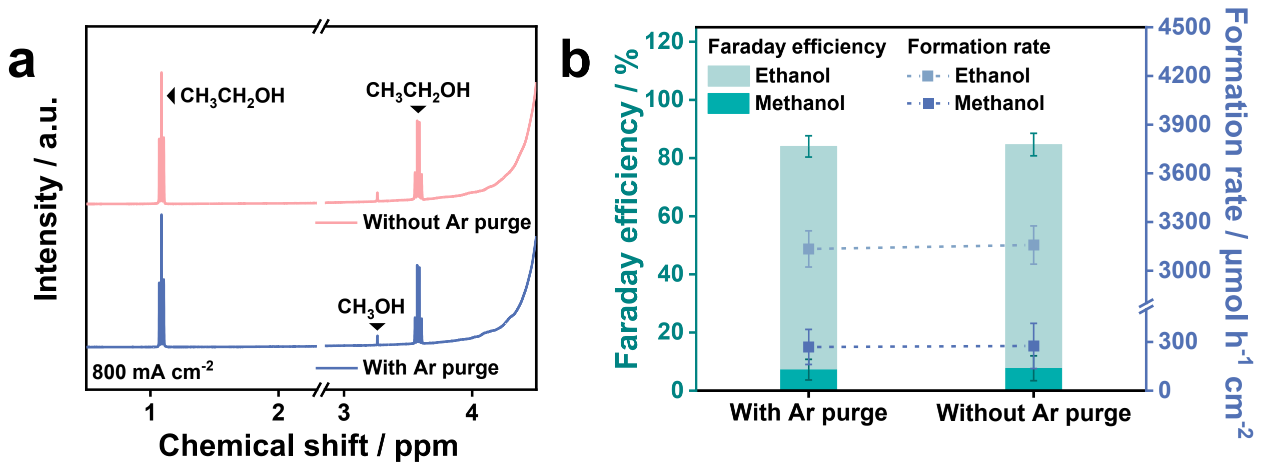

Fig. S67 (a) ^{1}H NMR spectra, as well as (b) FEs and formation rates calculated based on NMR results for PdCu/CNT during electrolysis with and without Ar purging.

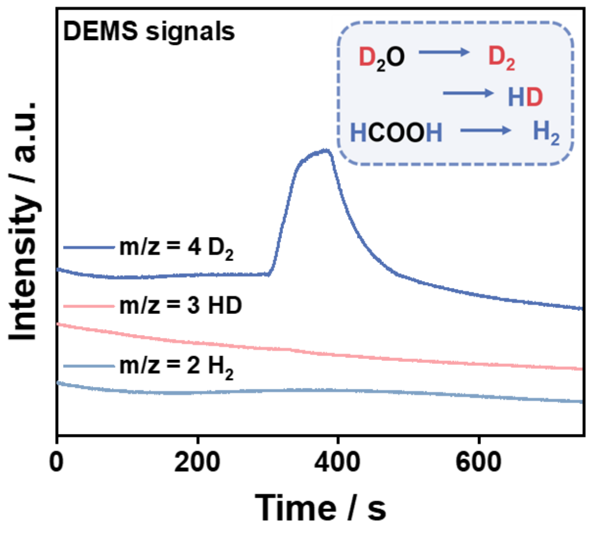

Fig. S68 Real-time mass signals at m/z = 2 (H_2), 3 (HD), and 4 (D_2) over PdCu/CNT in DEMS test.

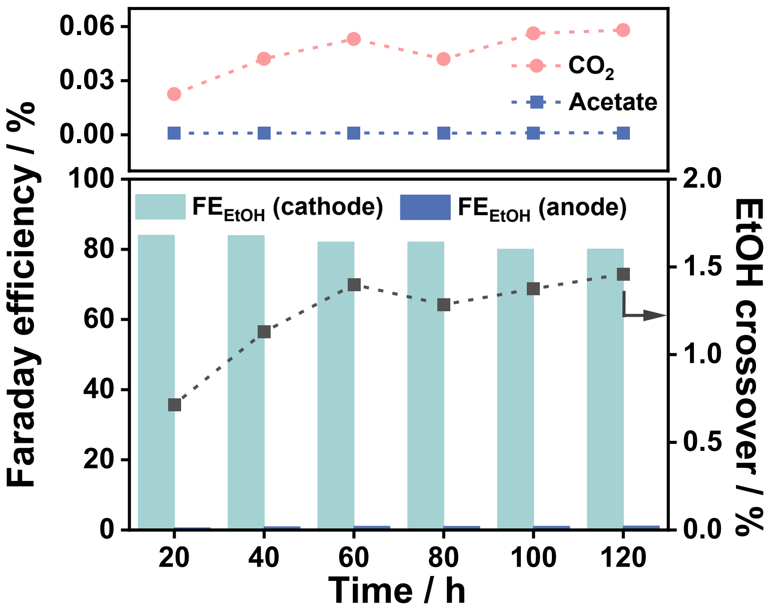

Fig. S69 FE of possible oxidized products from EtOH (top). FE distribution of EtOH at the cathode and anode, and crossover percentage of EtOH (bottom).

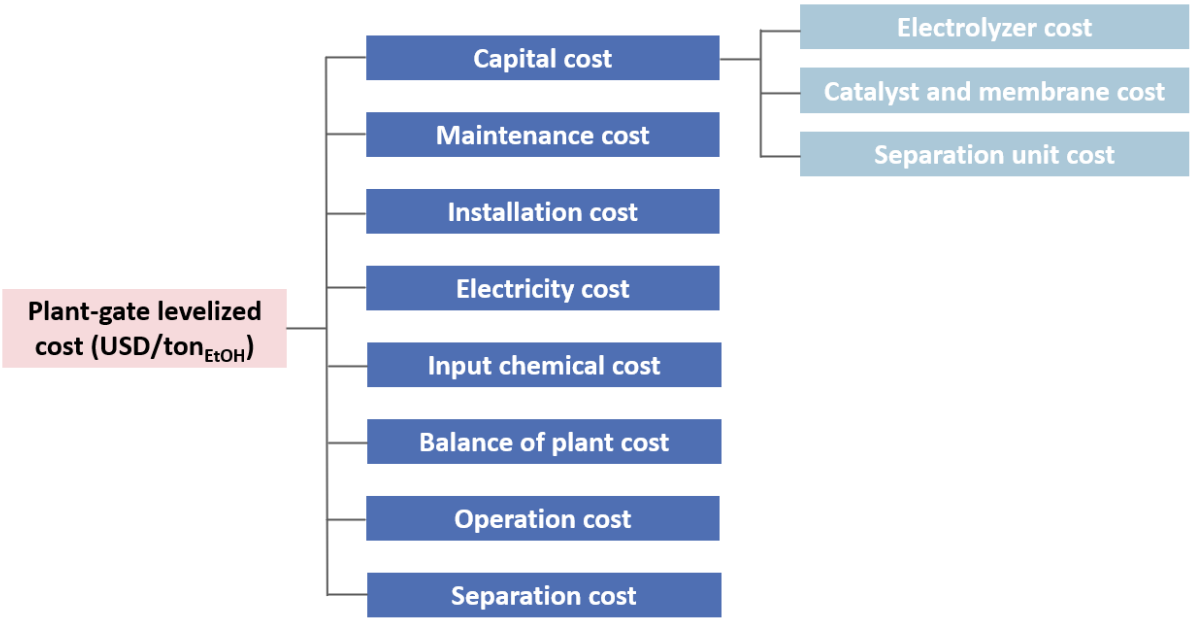

Fig. S70 Techno-economic analysis model for the EtOH electrosynthesis by FA electrocatalytic coupling reaction.

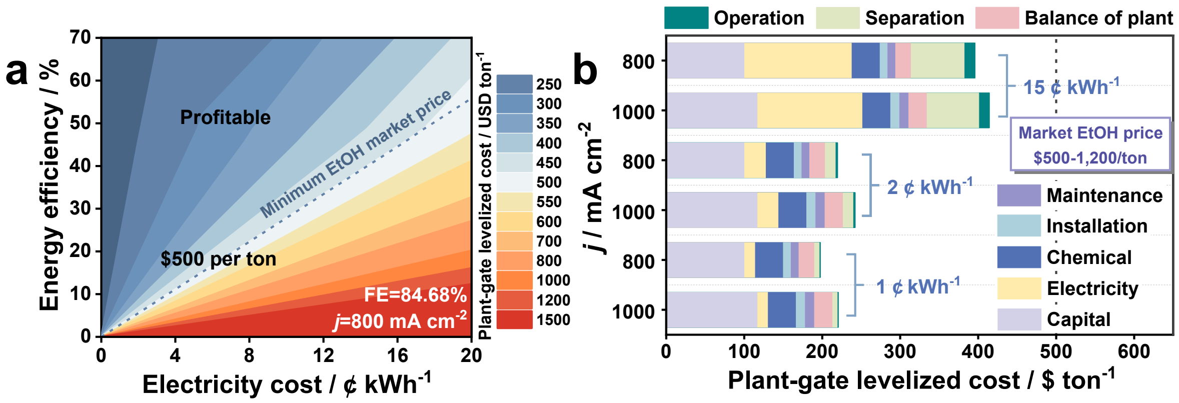

Fig. S71 (a) Plant-gate levelized cost as a function of electricity cost and energy efficiency. We assume an EtOH FE of 84.68% at current densities of 800 mA cm^{-2}. (b) Plant-gate levelized cost calculated from TEA with electricity cost of 1, 2, and 15 cents kWh^{-1} at current densities of 800 and 1,000 mA cm^{-2}.

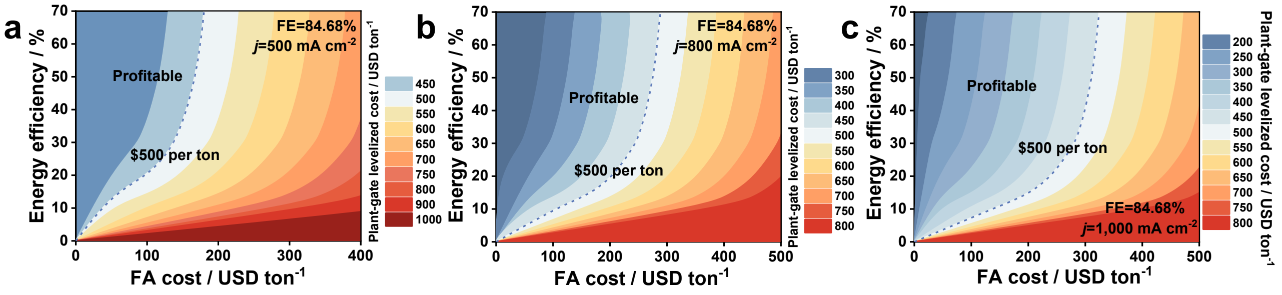

Fig. S72 (a-c) Plant-gate levelized cost as a function of FA cost and energy efficiency. We assume an FE of 84.68% for EtOH and current densities of 500, 800, and 1,000 mA cm^{-2}, respectively.

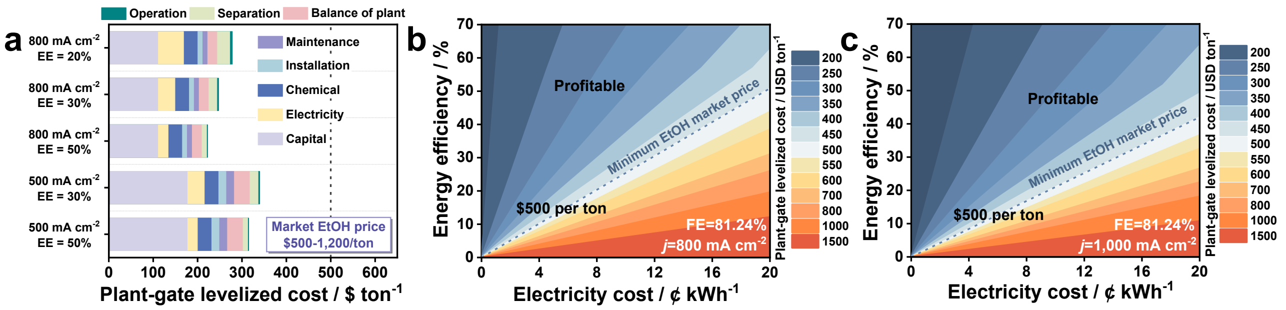

Fig. S73 TEA assessment for formaldehyde electroreduction. (a) TEA of formaldehyde electroreduction for EtOH production with different conditions. (b-c) Plant-gate levelized cost as a function of electricity cost and energy efficiency. We assume that at current densities of 800 and 1,000 mA cm^{-2}, the EtOH FE is 81.24%.

Table S1. ICP-AES characterized atomic ratios of PdCu.

Catalyst	Pd (atom%)	Cu (atom%)
$Pd_{0.8}Cu$/CNT	55.06	44.94
$Pd_{0.9}Cu_3$/CNT	51.87	48.13
**PdCu/CNT**	**50.76**	**49.23**
$Pd_{1.1}Cu$/CNT	47.82	52.18
$Pd_{1.2}Cu$/CNT	45.75	54.25

Table S2. Comparison of the electrocatalytic performance of related studies on the generation of EtOH from C_1 carbon sources. (The serial numbers of the No. columns correspond to Fig. 1g and Figure S22.)

No.	Catalyst	Electrolyte	Electrolyzer	C_1 feedstock	E / V vs. RHE	*j* / mA cm^{-2}	FE / %	Ref.
1	polycrystalline -Cu	0.1M KOH	H-cell	CO	-0.63	1.05	16.5	[4]
2	Cu Nanoflowers	0.1M KOH	H-cell	CO	-0.23	0.21	15.38	[5]
3	Cu particles/GDE	1.0 M KOH	H-cell	CO	-0.7	28	7.4	[6]
4	Cu_2O/Cu	2.0 M KOH	Flow cell	CO	-0.7	161	68.8	[7]
5	$Cu(OD)_{0.8}Ag_{0.2}$	1.0 M KOH	H-cell	CO	-2.98	150	20	[8]
6	Cu(B)-2	K_2HPO_4	H-cell	CO_2	-1.1	70	28	[9]
7	CuN_3	0.1 M	H-cell	CO_2	-0.95	14	66	[10]

		$KHCO_3$						
8	Cu(Ag-20)$_{20}$	0.1 M $KHCO_3$	H-cell	CO_2	-1.1	4.1	16.5	[11]
9	Cu/BaO*x*	1.0 M KOH	Flow cell	CO_2	-0.75	325	43	[12]
10	dCu$_2$O/Ag$_{2.3\%}$	1.0 M KOH	Flow cell	CO_2	-0.87	640	40.8	[13]
11	Hex-2Cu-O	1.0 M KOH	Flow cell	CO_2	-1.2	9.4	32.5	[14]
12	Cu$_{oh}$−Ag	0.1 M $KHCO_3$	H-cell	CO_2	-1.4	2.5	23.1	[15]
	NiO/Ni	0.1 M NaOH	H-cell	CH_4	1.4	3.0	89	[16]
	$Fe_3Ni_7(OH)_x$	0.1 M NaOH	H-cell	CH_4	1.46	NA	87	[17]
13	Cu$_2$-CuN$_3$	0.1 M $KHCO_3$	H-cell	CO_2	-1.1	20.6	51	[18]
14	$CuAl_2O_4$/CuO	1.0 M KOH	Flow cell	CO_2	-1.15	200	42	[19]
15	Cu-GB	0.1 M KOH	H-cell	CO_2	-3.72	507	26.8	[20]
16	Cu/Au	1.0 M KOH	Flow cell	CO_2	-0.75	500	60	[21]
17	TWN-Cu$_{13.35}$-600-SACs	CO_2 saturated 0.5 M $CsHCO_3$	H-cell	CO_2	-1.1	35	81.9	[22]
18	SnS_2 /Sn$_1$ -O$_3$G	0.5 M $KHCO_3$	H-cell	CO_2	0.9	17.8	82.5	[23]
19	Fe-tetraphenyl porphyrin /Ni	0.5 M $KHCO_3$	Flow cell	CO_2	-0.3	31	71.2	[24]

20	CuOCu-N_4	1.0 M KOH	Flow cell	CO_2	-0.3	10.8	56.3	[25]
21	Ag_1Cu nanowires	1.0 M KOH	Flow cell	CO_2	-1.0	173	56.3	[26]
22	Cu nanosheets/Ag	0.1 M KOH	Flow cell	CO_2	-1.1	630	57.5	[27]
23	Cu-TAPT	1.0 M KOH	Flow cell	CO_2	-0.47	429	57.3	[28]
24	O-$CuI_{1.3}$	1.0 M KOH + 3 M formaldehyde	H-cell	CO_2 and formaldehyde	-1.12	8.8	32.4	[29]
	PdCu/CNT	1.0 M KOH + 1.0 M FA	H-cell	Formic acid	-0.4	500	84.68	This work

Table S3. Lattice parameters of Pd-based catalysts.

**Samples**	**α / Å**
Pd/CNT	3.89
PdCu/CNT	3.75
$PdH_{0.45}Cu$/CNT	3.86
$PdH_{0.68}$/CNT	4.07

Table S4. Ion m/z used for EtOH identification.

**Compound**	**Characteristic fragments (m/z)**	**Molecular ions (m/z)**
CH_3CH_2OH	31	46
$^{13}CH_3CH_2OH$ or $CH_3{}^{13}CH_2OH$	32	47

$^{13}CH_3{}^{13}CH_2OH$	33	48

Table S5. *In situ* FTIR detection of intermediates and corresponding band positions for FRR and formaldehyde reduction reaction.

Assignment	Band center	Ref.
H-O-H bending vibration of H_2O	~1639 cm^{-1}	[30]
C-O symmetric stretching of *HCOO	~1378 cm^{-1}	[31, 32]
C-O asymmetric stretching of *HCOO	~1585 cm^{-1}	
*OCCOH of *HOOC-$C(OH)_2$	~1565 and ~1182 cm^{-1}	[13]
*OC_2H_5	~1349 cm^{-1}	[13]
O-C-O asymmetric stretching of *$C(OH)_2$	~1047 cm^{-1}	[33, 34]
O-C-O symmetric stretching of *$C(OH)_2$	~1035 cm^{-1}	
*CHO	~1715 cm^{-1}	[35, 36]
stretching vibration region of *CO (not observed)	~2000-2100 cm^{-1}	[31]

Table S6. *In situ* Raman spectroscopy band assignment for FRR.

Assignment	Raman shift	Ref.
OCO stretching of *$C(OH)_2$	~894 cm^{-1}	[37]
*HCOO asymmetric stretching	~1044 cm^{-1}	[30]
*HCOO symmetric stretching	~1356cm^{-1}	[38]
C-C stretching	~1557 cm^{-1}	[39, 40]
*CO vibration range (not observed)	~1900-2150 cm^{-1}	[41]

Table S7. Fukui function values for structure 1 ($PdH_{0.45}Cu$-*$C(OH)_2$).

Atom	$\rho_{(N)}$	$\rho_{(N+1)}$	$\rho_{(N-1)}$	f^-	f^+	f^0	CDD
1(Pd1)	-0.0083	-0.1841	0.1225	0.1308	0.1758	0.1533	0.045
2(Pd2)	0.2054	0.2014	0.2692	0.0638	0.004	0.0339	-0.0598
3(Pd3)	-0.0285	-0.1393	0.1067	0.1352	0.1108	0.123	-0.0243
4(Cu1)	-0.0679	-0.2439	0.0914	0.1593	0.1759	0.1676	0.0166
5(Cu2)	-0.0577	-0.1648	0.0509	0.1087	0.1071	0.1079	-0.0016
6(Cu3)	-0.0703	-0.2125	0.0346	0.1048	0.1422	0.1235	0.0374
7(Cu4)	-0.0733	-0.2833	0.052	0.1253	0.21	0.1676	0.0847
**8(C1)**	**0.0806**	**0.0736**	**0.1319**	**0.0513**	**0.007**	**0.0291**	**-0.0442**
9(O1)	-0.142	-0.1541	-0.1135	0.0286	0.012	0.0203	-0.0165
10(O2)	-0.1566	-0.1755	-0.1123	0.0443	0.019	0.0316	-0.0253
11(H1)	0.183	0.1634	0.2146	0.0316	0.0196	0.0256	-0.012
12(H2)	0.1356	0.1201	0.1519	0.0163	0.0156	0.0159	-0.0008

Table S8. Fukui function values for structure 2 ($PdH_{0.45}Cu$-*HCOO).

Atom	$\rho_{(N)}$	$\rho_{(N+1)}$	$\rho_{(N-1)}$	f^-	f^+	f^0	CDD
1(Pd1)	0.2378	0.1663	0.2425	0.0047	0.0715	0.0381	0.0668
2(Pd2)	0.1553	0.0358	0.271	0.1157	0.1195	0.1176	0.0038
3(Pd3)	-0.0433	-0.1519	0.0975	0.1408	0.1086	0.1247	-0.0321
4(Cu1)	-0.0268	-0.1609	0.1213	0.1481	0.1341	0.1411	-0.014
5(Cu2)	-0.041	-0.1549	0.0808	0.1218	0.1139	0.1178	-0.0079

6(Cu3)	-0.0748	-0.2111	0.1085	0.1833	0.1363	0.1598	-0.0469
7(Cu4)	-0.0547	-0.1896	0.1407	0.1953	0.1349	0.1651	-0.0604
**8(C1)**	**0.0914**	**0.0355**	**0.1038**	**0.0124**	**0.0559**	**0.0341**	**0.0435**
9(O1)	-0.273	-0.3183	-0.2339	0.039	0.0454	0.0422	0.0064
10(O2)	-0.1567	-0.1897	-0.1388	0.0178	0.033	0.0254	0.0152
11(H1)	0.1859	0.1402	0.2068	0.0209	0.0458	0.0333	0.0249

Table S9. Fukui function values for $PdH_{0.45}Cu$-*CH_2O (electrophilicity).

Atom	$\rho_{(N)}$	$\rho_{(N+1)}$	$\rho_{(N-1)}$	f^-	f^+	f^0	CDD
**1(C)**	**0.1313**	**-0.2687**	**0.3616**	**0.2303**	**0.4**	**0.3151**	**0.1697**
2(H)	0.0423	-0.115	0.2103	0.168	0.1573	0.1627	-0.0107
3(H)	0.0423	-0.115	0.2103	0.168	0.1573	0.1627	-0.0106
4(O)	-0.216	-0.5013	0.2177	0.4337	0.2853	0.3595	-0.1484

Table S10. Fukui function values for $PdH_{0.45}Cu$-*$CH_2(OH)_2$ (nucleophilicity).

Atom	$\rho_{(N)}$	$\rho_{(N+1)}$	$\rho_{(N-1)}$	f^-	f^+	f^0	CDD
**1(C)**	**0.089**	**-0.0026**	**0.1979**	**0.1089**	**0.0915**	**0.1002**	**-0.0174**
2(H)	0.0157	-0.1074	0.1539	0.1382	0.1232	0.1307	-0.015
3(H)	0.0157	-0.1076	0.1538	0.1381	0.1233	0.1307	-0.0148
4(O)	-0.2238	-0.3069	0.0027	0.2265	0.0832	0.1548	-0.1434
5(H)	0.1636	-0.0843	0.2445	0.0809	0.2478	0.1644	0.167
6(O)	-0.2238	-0.3069	0.0027	0.2265	0.0832	0.1548	-0.1434

7(H)	0.1636	-0.0843	0.2445	0.0809	0.2478	0.1644	0.167

Table S11. The main parameters of electrochemical EtOH production.

**Input Costs**	**Value**	**Ref.**
KOH ($/ton)	1,280	[42]
H_2O ($/ton)	1.18	[43]
Target product EtOH ($/ton)	500-1,200	Information is sourced from multiple channels, including professional websites, industry associations, government agencies, and suppliers.
Renewable electricity (¢/kWh)	2	[44]

**Plant Parameters**	**Value**	**Ref.**
Electrolyzer ($/m^2)	10,000	[45]
Catalysts and membrane ($/m^2)	3,000	

References

1 McCrory C C L, Jung S, Peters J C *et al.* Benchmarking heterogeneous electrocatalysts for the oxygen evolution reaction. *J Am Chem Soc* 2013; **135**: 16977−16987.

2 Fan J, Wu J, Cui X *et al.* Hydrogen stabilized RhPdH 2D bimetallene nanosheets for efficient alkaline hydrogen evolution. *J Am Chem Soc* 2020; **142**: 3645−3651.

3 Liu J, Chen D, Tang T *et al.* Green chemical process for continuous production of high-purity 2,5-furandicarboxylic acid in anion exchange membrane flow electrolyzer. *Nat Commun* 2026; DOI: 10.1038/s41467-026-68894-3.

4 Wang L, Nitopi S A, Bertheussen E *et al.* Electrochemical carbon monoxide reduction on polycrystalline copper: effects of potential, pressure, and pH on selectivity toward multicarbon and

oxygenated products. *ACS Catal* 2018; **8**: 7445−7454.

5 Wang L, Nitopi S, Wong A B *et al.* Electrochemically converting carbon monoxide to liquid fuels by directing selectivity with electrode surface area. *Nat Catal* 2019; **2**: 702–708.

6 Chen R, Su H-Y, Liu D *et al.* Highly selective production of ethylene by the electroreduction of carbon monoxide. *Angew Chem Int Ed* 2020; **59**: 154–160.

7 Ma G, Syzgantseva O A, Huang Y *et al.* A hydrophobic Cu/Cu_2O sheet catalyst for selective electroreduction of CO to ethanol. *Nat Commun* 2023; **14**: 501.

8 Li J, Xiong H, Liu X *et al.* Weak CO binding sites induced by Cu-Ag interfaces promote CO electroreduction to multi-carbon liquid products. *Nat Commun* 2023; **14**: 698.

9 Zhou Y, Che F, Liu M *et al.* Dopant-induced electron localization drives CO_2 reduction to C_2 hydrocarbons. *Nat Chem* 2018; **10**: 974–980.

10 Liang Z-Q, Zhuang T-T, Seifitokaldani A *et al.* Copper-on-nitride enhances the stable electrosynthesis of multi-carbon products from CO_2. *Nat Commun* 2018; **9**: 3828.

11 Ting L R L, Piqué O, Lim S Y *et al.* Enhancing CO_2 electroreduction to ethanol on copper-silver composites by opening an alternative catalytic pathway. *ACS Catal* 2020; **10**: 4059−4069.

12 Xu A, Hung S-F, Cao A *et al.* Copper/alkaline earth metal oxide interfaces for electrochemical CO_2-to-alcohol conversion by selective hydrogenation. *Nat Catal* 2022; **5**: 1081–1088.

13 Wang P, Yang H, Tang C *et al.* Boosting electrocatalytic CO_2-to-ethanol production via asymmetric C-C coupling. *Nat Commun* 2022; **13**: 3754.

14 Yang B, Chen L, Xue S *et al.* Electrocatalytic CO_2 reduction to alcohols by modulating the molecular geometry and Cu coordination in bicentric copper complexes. *Nat Commun* 2022; **13**: 5122.

15 Iyengar P, Kolb M J, Pankhurst J R *et al.* Elucidating the facet-dependent selectivity for CO_2 electroreduction to ethanol of Cu-Ag tandem catalysts. *ACS Catal* 2021; 4456−4463.

16 Song Y, Zhao Y, Nan G *et al.* Electrocatalytic oxidation of methane to ethanol via NiO/Ni interface. *Appl Catal B Environ* 2020; **270**: 118888.

17 Li J, Yao L, Wu D *et al.* Electrocatalytic methane oxidation to ethanol on iron-nickel hydroxide nanosheets. *Appl Catal B Environ* 2022; **316**: 121657.

18 Su X, Jiang Z, Zhou J *et al.* Complementary operando spectroscopy identification of in-situ generated metastable charge-asymmetry Cu_2-CuN_3 clusters for CO_2 reduction to ethanol. *Nat Commun* 2022; **13**: 1322.

19 Zhang T, Yuan B, Wang W *et al.* Tailoring *H intermediate coverage on the $CuAl_2O_4$/CuO catalyst for enhanced electrocatalytic CO_2 reduction to ethanol. *Angew Chem Int Ed* 2023; **62**: e202302096.

20 Cheng D, Zhang G, Li L *et al.* Guiding catalytic CO_2 reduction to ethanol with copper grain boundaries. *Chem Sci* 2023; **14**: 7966–7972.

21 Kuang S, Su Y, Li M *et al.* Asymmetrical electrohydrogenation of CO_2 to ethanol with copper-gold heterojunctions. *Proc Natl Acad Sci USA* 2023; **120**: e2214175120.

22 Xia W, Xie Y, Jia S *et al.* Adjacent copper single atoms promote C-C coupling in electrochemical CO_2 reduction for the efficient conversion of ethanol. *J Am Chem Soc* 2023; **145**: 17253−17264.

23 Ding J, Yang H B, Ma X-L *et al.* A tin-based tandem electrocatalyst for CO_2 reduction to ethanol with 80% selectivity. *Nat Energy* 2023; **8**: 1386–1394.

24 Abdinejad M, Farzi A, Möller-Gulland R *et al.* Eliminating redox-mediated electron transfer mechanisms on a supported molecular catalyst enables CO_2 conversion to ethanol. *Nat Catal* 2024; **7**: 1109–1119.

25 Xu F, Feng B, Shen Z *et al.* Oxygen-bridged Cu binuclear sites for efficient electrocatalytic CO_2 reduction to ethanol at ultralow overpotential. *J Am Chem Soc* 2024; **146**: 9365−9374.

26 Wang S, Li F, Zhao J *et al.* Manipulating C-C coupling pathway in electrochemical CO_2 reduction for selective ethylene and ethanol production over single-atom alloy catalyst. *Nat Commun* 2024; **15**: 10247.

27 Luan P, Dong X, Liu L *et al.* Selective electrosynthesis of ethanol via asymmetric C-C coupling in tandem CO_2 reduction. *ACS Catal* 2024; **14**: 8776−8785.

28 Chen X, Jia S, Zhai J *et al.* Multivalent Cu sites synergistically adjust carbonaceous intermediates adsorption for electrocatalytic ethanol production. *Nat Commun* 2024; **15**: 7691.

29 Li Z, Wu J. Co-electrolysis of CO_2 and formaldehyde to ethanol: An inspiring asymmetric C-C coupling pathway. *Chem Catal* 2022; **2**: 2808-2810.

30 Yu J, Zhang P, Li L *et al.* Electroreductive coupling of benzaldehyde by balancing the formation and dimerization of the ketyl intermediate. *Nat Commun* 2022; **13**: 7909.

31 Li S, Feng L, Wang H *et al.* Atomically intimate assembly of dual metal-oxide interfaces for tandem conversion of syngas to ethanol. *Nat Nanotechnol* 2025; **20**: 255–264.

32 Yi J-D, Xie R, Xie Z-L *et al.* Highly selective CO_2 electroreduction to CH_4 by in situ generated Cu_2O single-type sites on a conductive MOF: Stabilizing key intermediates with hydrogen bonding. *Angew Chem Int Ed* 2020; **59**: 23641–23648.

33 Chen Y-F, Chu L-K. Infrared characterization of isotopic analogues of methanediol in aqueous solution. *J Phys Chem A* 2022; **126**: 5302−5309.

34 Chen P-R, Chu L-K. Infrared characterization of hydrated products of glyoxal in aqueous solution. *Spectrochim Acta A Mol Biomol Spectrosc* 2024; **306**: 123571.

35 Zhu S, Jiang B, Cai W-B *et al.* Direct observation on reaction intermediates and the role of bicarbonate anions in CO_2 electrochemical reduction reaction on Cu surfaces. *J Am Chem Soc* 2017; **139**: 15664–15667.

36 Wang C, Zou X, Liu H *et al.* A highly efficient catalyst of palygorskite-supported manganese oxide for formaldehyde oxidation at ambient and low temperature: Performance, mechanism and reaction kinetics. *Appl Surf Sci* 2019; **486**: 420−430.

37 Lebrun N, Dhamelincourt P, Focsa C *et al.* Raman analysis of formaldehyde aqueous solutions as a function of concentration. *J Raman Spectrosc* 2003; **34**: 459–464.

38 Zhao L, Lv Z, Shi Y *et al.* Simultaneous generation of furfuryl alcohol, formate, and H_2 by co-electrolysis of furfuryl and HCHO over bifunctional CuAg bimetallic electrocatalysts at ultra-low voltage. *Energy Environ Sci* 2024; **17**: 770–779.

39 Ma X, Xu L, Liu S *et al.* Electrochemical C-C coupling between CO_2 and formaldehyde into ethanol. *Chem Catal* 2022; **2**: 3207–3224.

40 Zhao Y, Zhang X-G, Bodappa N *et al.* Elucidating electrochemical CO_2 reduction reaction processes on Cu(hkl) single-crystal surfaces by in situ Raman spectroscopy. *Energy Environ Sci* 2022; **15**: 3968–3977.

41 Herzog A, Lopez Luna M, Jeon H S *et al.* Operando Raman spectroscopy uncovers hydroxide

and CO species enhance ethanol selectivity during pulsed CO_2 electroreduction. *Nat Commun* 2024; **15**: 3986.

42 Ye N, Wang K, Tan Y *et al.* Industrial-level CO_2 to formate conversion on turing-structured electrocatalysts. *Nat Synth* 2025; **4**: 799–807.

43 Pribyl-Kranewitter B, Beard A, Gîjiu C L *et al.* Influence of low-temperature electrolyser design on economic and environmental potential of CO and HCOOH production: A techno-economic assessment. *Renew Sust Energ Rev* 2022; **154**: 111807.

44 Wang J, Bui H T D, Hu H *et al.* Industrial-current ammonia synthesis by polarized cuprous cyanamide coupled to valorization of glycerol at 4,000 mA cm^{-2}. *Adv Mater* 2025; **37**: 2418451.

45 Lee B-H, Shin H, Rasouli A S *et al.* Supramolecular tuning of supported metal phthalocyanine catalysts for hydrogen peroxide electrosynthesis. *Nat Catal* 2023; **6**: 234–243.
